# Discovery of guanidinium salt incorporating cinnamic acid skeleton as new antibacterial agents against Gram-positive bacteria

**DOI:** 10.3389/fmicb.2026.1906096

**Published:** 2026-07-15

**Authors:** Fen Zhou, Ping Zhao, Mengqi Liu, Hongxin Li, Yinhu Wang, Liang Liang

**Affiliations:** 1 Department of Pharmacy, Liaocheng People’s Hospital, Liaocheng, Shandong, China; 2 School of Pharmaceutical Sciences and Food Engineering, Liaocheng University, Liaocheng, China

**Keywords:** antimicrobial peptides, cinnamic acid, guanidinium, membrane-targeting, multidrug-resistant bacteria

## Abstract

The global infection rates caused by multidrug-resistant bacteria continue to rise, while the pipeline for novel antibiotics is increasingly drying up, highlighting the urgent need to develop new antimicrobial agents. Drawing inspiration from the amphiphilic structure of cationic antimicrobial peptides (AMPs), a collection of amphiphilic guanidinium salts incorporating cinnamic acid skeleton was designed and prepared. Bioactivity screening revealed that compound **10c** exhibited excellent inhibitory effects against Gram-positive bacteria, with MIC values ranging from 1 to 2 μg/mL, comparable to the clinically used drug vancomycin. Further evaluation demonstrated that **10c** possessed negligible hemolytic activity, infrequent resistance acquisition, low cytotoxicity, fast bactericidal action, and good plasma stability, indicating strong potential for further development. Additionally, **10c** not only effectively prevented biofilm formation but also significantly disrupted pre-formed biofilms. Mechanistic studies revealed that **10c** achieved selective membrane targeting by specifically interacting with phosphatidylglycerol present in the bacterial cell membrane. This interaction triggered membrane depolarization, increased membrane permeability, leading to elevated intracellular ROS levels and escape of cellular contents, ultimately accelerating bacterial death. More importantly, **10c** significantly reduced bacterial burden and mitigated tissue inflammation, outperforming vancomycin in a murine skin abscess model. In summary, these results indicated that compound **10c** was a membrane-active antimicrobial candidate with promising potential for further development.

## 1 Introduction

The widespread and frequently inappropriate use of antibiotics has precipitated a global health crisis characterized by the rapid proliferation and dissemination of multidrug-resistant bacterial pathogens (Süssmuth et al., 2025). This phenomenon has critically eroded the effectiveness of conventional antimicrobial therapies, leading to a concerning escalation in treatment failures and mortality rates associated with bacterial infections (Fernow et al., 2025). Current estimates underscore the magnitude of this threat, with antimicrobial resistance (AMR) directly responsible for approximately 1.27 million annual deaths globally and implicated in an additional 3.68 million fatalities (Okeke et al., 2024; Wahid et al., 2024). In response, the World Health Organization has designated a group of high-priority pathogens requiring urgent attention and innovative countermeasures. This “critical priority” list includes carbapenem-resistant Enterobacteriaceae (CRE), methicillin-resistant *S. aureus* (MRSA), vancomycin-resistant *Enterococci* (VRE), as well as drug-resistant *P. aeruginosa* and *A. baumannii* (Liu et al., 2020; Antimicrobial Resistance Collaborators, 2022). These organisms persistently drive significant morbidity and mortality in both healthcare-associated and community settings. However, in stark contrast to this severe epidemiological reality, the global pipeline for developing novel antimicrobial drugs is increasingly depleted (Maharramov et al., 2025). The past 30 years have witnessed a pronounced scarcity in the introduction of antibiotics with fundamentally new mechanisms of action, creating a dangerous therapeutic void (Ho et al., 2025). Consequently, the imperative to discover and develop new antimicrobial agents endowed with novel mechanism has never been more pressing, representing a paramount challenge for contemporary biomedical research.

Antimicrobial peptides (AMPs), serving as crucial effector components within the innate immune defense system, are broadly distributed across diverse organisms ranging from plants and insects to mammals, forming the primary chemical barrier against pathogenic invasion (Oliveira Júnior et al., 2025). These short cationic peptides, typically composed of 12 to 50 amino acids, not only exhibit potent activity against bacteria and fungi but also demonstrate cytotoxicity toward certain tumor cells (Dwivedi et al., 2024). Consequently, they are regarded as a highly promising class of novel therapeutic candidates for combating multidrug-resistant infections. Their antimicrobial action primarily stems from the direct physical disruption of microbial cell membranes (Ji S. et al., 2024). Their cationic nature enables them to electrostatically target and bind to bacterial membranes, which are rich in anionic phospholipids and lipopolysaccharides (Ouyang et al., 2025). Subsequently, their hydrophobic regions promote insertion into the lipid bilayer, disrupting its native organization (Alzain et al., 2025). This perturbation leads to loss of membrane integrity, collapse of ion gradients, leakage of intracellular contents, and ultimately bacterial cell death (Yang et al., 2025). This membrane-targeting mechanism offers two distinct advantages: first, AMPs are less prone to inducing high-level conventional resistance, as bacteria cannot easily alter the fundamental composition and surface charge of their membranes through simple genetic mutations; second, this mechanism confers selectivity between microbial and host cells. The outer leaflet of mammalian cell membranes is predominantly composed of electrically neutral phospholipids (e.g., phosphatidylcholine) and contains cholesterol, which stabilizes membrane structure, rendering them relatively insensitive to such electrostatically driven membrane disruption and thereby providing a potential therapeutic window (Szymczak et al., 2025; Dubey et al., 2026).

Despite their unique mechanism, the clinical translation of natural AMPs faces several challenges (Zhang et al., 2025). They are susceptible to rapid proteolytic degradation *in vivo*, resulting in poor systemic stability and extremely short half-lives (Yang et al., 2024b). The therapeutic window between effective antimicrobial concentrations and those inducing hemolytic or cytotoxic effects is often narrow (Xie et al., 2020). Furthermore, their complex structures lead to high costs for large-scale synthesis (Yang R. et al., 2024). To overcome these limitations, the field of medicinal chemistry is actively advancing the development of small-molecule antimicrobial peptide mimetics (SMAMPs) (Yang et al., 2024a). Through rational design, this methodology deconstructs the core pharmacophoric elements of AMPs, which are defined by the spatially precise organization of cationic and hydrophobic domains (Hedges et al., 2025). These optimized structural motifs are then integrated into novel molecular frameworks engineered to be synthetically tractable, metabolically stable, and endowed with superior drug-like characteristics (Xu et al., 2026). Current research efforts encompass several strategic directions: the design of foldamers and oligomers (Choi et al., 2009) based on aromatic or rigid units (e.g., phenylene ethynylenes (Ishitsuka et al., 2006), β-peptoids (Hansen et al., 2010)) to mimic the amphipathic topology of natural peptides; the development of advanced peptidomimetics with enhanced enzymatic stability via cyclization (Ji X. et al., 2024), incorporation of non-natural amino acids (Zhong et al., 2025), or backbone modifications (Wang et al., 2021); and the construction of small-molecule amphiphilic compounds derived entirely from non-peptide templates, such as structurally optimized aminoglycoside derivatives (Herzog et al., 2012), synthetic dendritic polymers (Tew et al., 2002), or specific scaffolds derived from natural products (Kong et al., 2023). The common goal of these efforts is to retain or even improve broad-spectrum activity and selectivity while significantly enhancing metabolic stability and safety. Encouragingly, four small-molecule mimetics (LTX-109 (Isaksson et al., 2011), CSA-13 (Chmielewska et al., 2020), XF-73 (Rhys-Williams et al., 2023), and PMX-30063 (Bassetti et al., 2020)) have advanced to the clinical trial stage, highlighting the feasibility of this approach in developing new antibacterial drugs.

**Figure 1:**
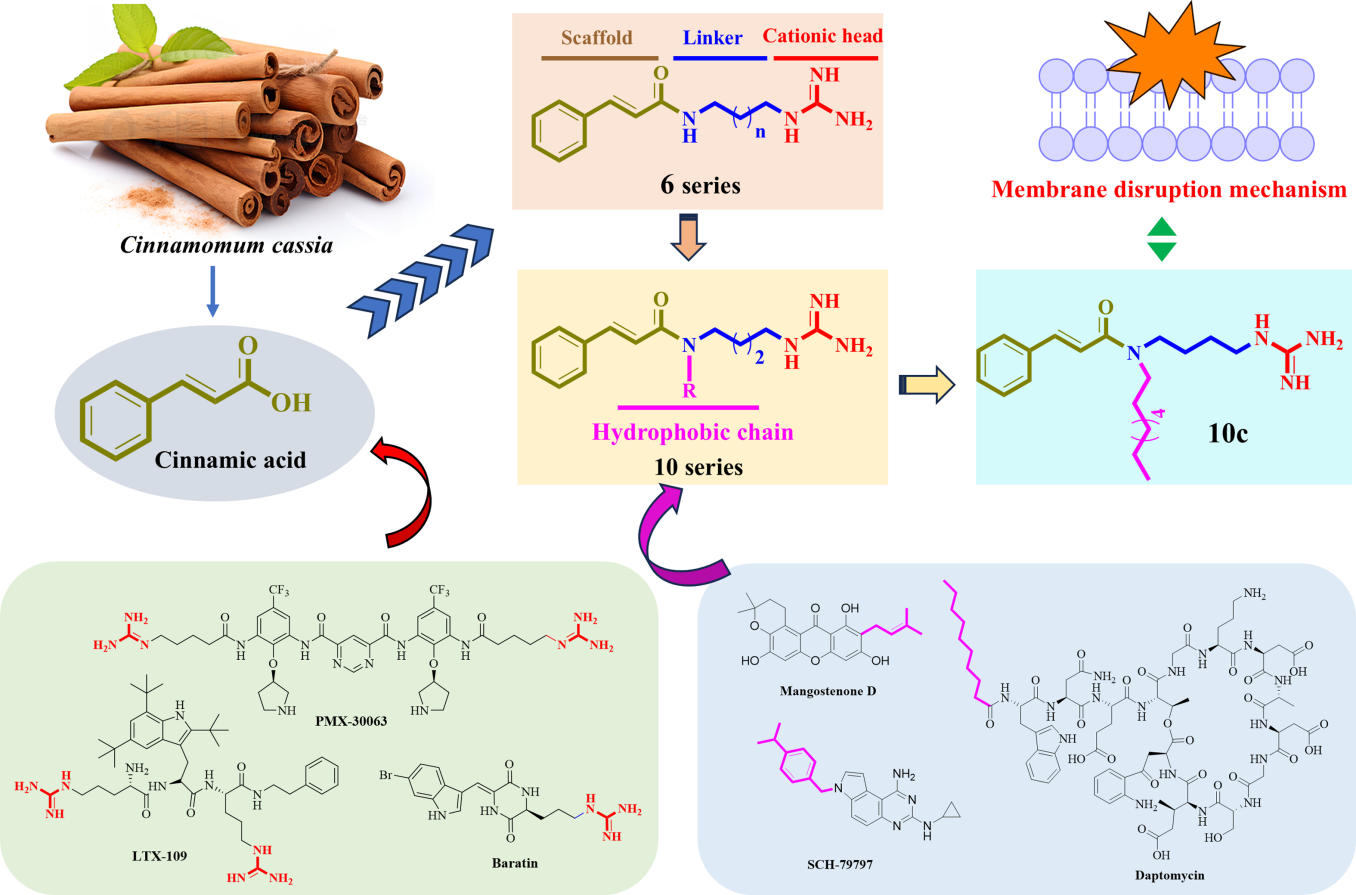
Design of cinnamic acid-guanidinium derivatives with membrane-active capability.

The structural diversity of natural products served as a primary source for the discovery of drugs possessing either novel chemical structures or unique mechanisms of action (Yang et al., 2023; Wang et al., 2024; Li et al., 2019). Cinnamic acid, a natural compound isolated from the dried bark of *Cinnamomum cassia*, displayed a broad range of pharmacological properties, such as anticancer, antimicrobial, anti-inflammatory, and metabolic modulation (Ruwizhi and Aderibigbe, 2020; Tian et al., 2025). In addition, its phenylpropenoic scaffold was recognized as a privileged scaffold in drug discovery, featuring in clinical agents such as flunarizine and ozagrel. Motivated by reports of its intrinsic, albeit moderate activity against pathogens such as *S. aureus*, *P. aeruginosa*, and *E. coli*, we hypothesized that the cinnamic acid scaffold could be strategically engineered to enhance antimicrobial potency. Inspired by the strong electrostatic attraction between the cationic guanidyl head of SMAMPs (LTX-109, PMX-30063, and Baratin (Labriere et al., 2020)) and the electronegative bacterial membrane. Our design rationale centered on transforming cinnamic acid scaffold into synthetic amphiphiles (6 series) by conjugating cationic guanidino moiety for electrostatic targeting with hydrophobic cinnamamide segment to promote lipid bilayer insertion. However, the results showed that these derivatives exhibited only moderate or low antibacterial activity. The weak activity of these compounds might be attributed to their inadequate overall hydrophobicity, which prevented the hydrophobic domain from effectively penetrating the bacterial membrane lipid bilayer. Therefore, increasing the hydrophobicity while carefully modulating the amphiphilic balance was crucial for achieving improved antibacterial efficacy. Extensive evidence supported that the introduction of hydrophobic tail such as alkyl, unsaturated alkyl, or aromatic group into molecular scaffold significantly facilitated membrane disruption. This principle was well exemplified by the natural product mangostenone D (Koh et al., 2013), the quinazoline derivative SCH-79797 (Martin et al., 2020), and the clinically approved antibiotic daptomycin (Jaber and Beahm, 2023). All of these agents displayed potent antibacterial activity, largely owing to their distinctive hydrophobic structural features. Drawing on these observations, we further constructed a library of cinnamic acid-guanidinium derivatives (10 series) by incorporating diverse hydrophobic chains (alkyl, unsaturated alkyl, and aromatic groups), thereby emulating the structural amphiphilicity of AMPs (Figure 1). These mimics were subsequently assessed against a range of bacterial species, and compound **10c** emerged as an optimized lead, demonstrating potent bactericidal effects coupled with minimal hemolytic and cytotoxic liabilities. Its promising profile was substantiated through comprehensive mechanistic studies, including time-kill kinetics, resistance propensity assessment, biofilm eradication assays, and investigations into its membrane-disruptive mode of action.

## 2 Materials and methods

### 2.1 Chemistry

All commercially available reagents, reference compounds, and fluorescent dyes were obtained from authorized suppliers and used without further purification. Reference antibiotics (vancomycin, norfloxacin, colistin) and melittin were purchased from Shanghai Aladdin Biochemical Technology Co., Ltd. (Shanghai, China). PI, DAPI, DiSC_3_(5), and DCFH-DA were obtained from Sigma-Aldrich (St. Louis, MO, United States). All other general reagents and solvents of analytical grade were procured from commercial suppliers and used as received. All reactions were routinely monitored by thin-layer chromatography performed on pre-coated silica gel GF254 plates (Qingdao Haiyang Chemical Co., Qingdao, China). High-resolution mass spectra (HRMS) were acquired on a Thermo DFS mass spectrometer (Thermo Fisher Scientific, Waltham, MA, United States). NMR spectra (^1^H and ^13^C) were recorded on a Bruker Avance 500 MHz spectrometer (Bruker Corporation, Bremerhaven, Germany), with chemical shifts referenced to the residual solvent peaks of CDCl_3_, DMSO-d_6_, or CD_3_OD.

#### 2.1.1 Synthesis for compounds **2a**–**2c**

In a standard experimental protocol, the respective diamino substrates **1a–1c** (11.34 mmol) were combined with DIPEA (2.93 g, 22.68 mmol) in dichloromethane. 1,3-di-Boc-2-methylisothiourea (2.64 g, 9.07 mmol) was added, and the resulting suspension was stirred at room temperature for 3 h. Upon completion as indicated by TLC monitoring, the reaction mixture was subsequently diluted with water and extracted with dichloromethane. The combined organic layers were concentrated in vacuo, and the crude product was subjected to purification via flash column chromatography. This procedure afforded the target compounds **2a–2c** as yellow solid (48–65%).

##### 2.1.1.1 1-(3-aminopropyl)-tert-butyl(diaminomethylene)carbamate (**2a**)

Yellow solid, yield 65%. ^1^H NMR (500 MHz, CDCl_3_) *δ* 11.48 (s, 1H), 8.92 (s, 1H), 3.35 (t, *J* = 5.8 Hz, 4H), 1.89 (p, *J* = 5.9 Hz, 2H), 1.50–1.42 (m, 18H). ^13^C NMR (125 MHz, CDCl_3_) *δ* 163.55, 158.91, 156.68, 83.08, 79.34, 38.69, 32.40, 28.57, 28.22, 28.01.

##### 2.1.1.2 1-(4-aminobutyl)-tert-butyl(diaminomethylene)carbamate (**2b**)

Yellow solid, yield 61%. ^1^H NMR (500 MHz, CDCl_3_) *δ* 11.50 (s, 1H), 8.35 (s, 1H), 3.43 (td, *J* = 7.3, 4.8 Hz, 2H), 2.75 (t, *J* = 6.9 Hz, 2H), 1.79–1.61 (m, 4H), 1.50 (dd, *J* = 4.4, 2.1 Hz, 18H). ^13^C NMR (125 MHz, CDCl_3_) *δ* 163.57, 156.13, 153.29, 83.05, 79.24, 41.64, 40.63, 30.59, 28.29, 28.06, 26.38.

##### 2.1.1.3 1-(5-aminopentyl)-tert-butyl(diaminomethylene)carbamate (**2c**)

Yellow solid, yield 48%. ^1^H NMR (500 MHz, CDCl_3_) *δ* 11.51 (s, 1H), 8.51–8.24 (m, 1H), 3.51–3.39 (m, 2H), 2.71 (td, *J* = 7.0, 2.8 Hz, 2H), 1.60 (pd, *J* = 7.2, 2.7 Hz, 4H), 1.50 (p, *J* = 5.4 Hz, 18H), 1.40 (tdd, *J* = 9.2, 7.5, 6.9, 3.4 Hz, 2H). ^13^C NMR (125 MHz, CDCl_3_) *δ* 163.57, 156.09, 153.27, 82.97, 79.14, 41.84, 40.76, 33.09, 33.06, 28.82, 28.25, 28.02, 24.08.

#### 2.1.2 Synthesis for cinnamoyl chloride (**4**)

Cinnamic acid **3** (1.00 g, 6.75 mmol) was charged into a round-bottom flask and treated with thionyl chloride (4 mL) in the presence of a catalytic amount of DMF (2 drops). The resulting mixture was stirred at 70 °C for 2 h. After the reaction reached completion, the volatiles were evaporated under reduced pressure, and the obtained crude residue was utilized directly in the subsequent step without further purification.

#### 2.1.3 General procedure for the preparation of compounds **5a–5c**

To a stirred solution of intermediates **2a–2c** (5.00 mmol) in CH_2_Cl_2_ (20 mL) was added triethylamine (1.01 g, 10.00 mmol) at 0 °C, and cinnamoyl chloride (0.83 g, 5.00 mmol) was added in a dropwise fashion. The reaction mixture was allowed to warm to room temperature and stir for an additional 10 h. The solution was removed in vacuo, and the residual oil was isolated via flash column chromatography on silica gel (eluent: ethyl acetate/petroleum ether) to yield compound **5a–5c** as a yellow oil in yields ranging from 53 to 86%.

##### 2.1.3.1 N-(3-tert-butyl(diaminomethylene)carbamate-propyl)cinnamamide (**5a**)

Light yellow oil, yield 77%. ^1^H NMR (500 MHz, CDCl_3_) *δ* 7.64 (d, J = 15.7 Hz, 1H), 7.53–7.46 (m, 2H), 7.32 (tt, *J* = 4.0, 2.2 Hz, 3H), 6.54 (d, *J* = 15.7 Hz, 1H), 3.60–3.33 (m, 4H), 1.65 (m, 2H), 1.52–1.40 (m, 18H), 1.24 (d, J = 8.3 Hz, 2H). ^13^C NMR (125 MHz, CDCl_3_) *δ* 166.22, 153.15, 140.50, 135.30, 129.40, 128.73, 127.93, 121.76, 38.40, 35.46, 30.03, 28.40, 28.31, 28.13, 27.97, 19.05.

##### 2.1.3.2 N-(4-tert-butyl(diaminomethylene)carbamate-butyl)cinnamamide (**5b**)

Light yellow oil, yield 86%. ^1^H NMR (500 MHz, CDCl_3_) *δ* 11.50 (s, 1H), 8.45 (t, *J* = 5.8 Hz, 1H), 7.63 (d, *J* = 15.7 Hz, 1H), 7.52–7.47 (m, 2H), 7.37–7.31 (m, 3H), 6.96 (t, 1H), 6.55 (d, 1H), 3.45 (dq, 4H), 1.70–1.60 (m, 4H), 1.48 (s, 18H). ^13^C NMR (125 MHz, CDCl_3_) *δ* 166.10, 163.31, 156.45, 153.24, 140.37, 135.07, 129.46, 128.75, 127.78, 121.35, 83.34, 79.58, 40.33, 39.52, 28.28, 28.08, 27.33, 25.67.

##### 2.1.3.3 N-(5-tert-butyl(diaminomethylene)carbamate-pentyl)cinnamamide (**5c**)

Light yellow oil, yield 53%. ^1^H NMR (500 MHz, DMSO-d_6_) *δ* 11.52 (s, 1H), 8.29 (q, *J* = 5.5 Hz, 1H), 8.12 (q, *J* = 5.3 Hz, 1H), 7.56 (dt, *J* = 6.1, 1.4 Hz, 2H), 7.44–7.39 (m, 3H), 7.39–7.36 (m, 1H), 6.63 (d, *J* = 15.8 Hz, 1H), 3.29 (dq, 2H), 3.19 (q, *J* = 6.6 Hz, 2H), 1.57–1.48 (m, 4H), 1.47 (d, *J* = 4.5 Hz, 9H), 1.40 (s, 9H), 1.32 (td, *J* = 8.4, 3.6 Hz, 2H). ^13^C NMR (125 MHz, DMSO-d_6_)) *δ* 165.26, 163.60, 155.70, 152.59, 138.83, 135.45, 129.78, 129.35, 127.90, 122.80, 83.30, 78.53, 39.03, 31.76, 29.24, 28.73, 28.55, 28.45, 28.12, 28.05, 24.24.

#### 2.1.4 General procedure for the preparation of compounds **6a–6c**

Compounds **5a–5c** (2.00 mmol) were individually subjected to deprotection using trifluoroacetic acid (4 mL) in dichloromethane (4 mL) at ambient temperature. Following completion of the reaction, the solution was removed under reduced pressure. The resulting crude residues were triturated with diethyl ether, and the solids were collected by filtration to afford the desired products **6a–6c** in isolated yields ranging from 63 to 82%.

##### 2.1.4.1 Amino((3-cinnamamidopropyl)amino)methaniminium (**6a**)

Yellow solid, yield 63%. ^1^H NMR (500 MHz, CD_3_OD) *δ* 7.56–7.47 (m, 3H), 7.38–7.33 (m, 3H), 6.59 (d, *J* = 15.7 Hz, 1H), 3.35 (t, *J* = 6.8 Hz, 2H), 3.22 (t, *J* = 6.9 Hz, 2H), 1.81 (p, *J* = 6.9 Hz, 2H). ^13^C NMR (125 MHz, CD_3_OD) *δ* 167.66, 157.42, 140.61, 134.87, 129.59, 128.65, 127.52, 120.31, 38.66, 38.05, 36.29, 28.65, 19.61. HRMS (ESI) C_13_H_19_N_4_O [M + H]^+^ calcd = 247.1554; found [M + H]^+^ = 247.1559.

##### 2.1.4.2 Amino((4-cinnamamidobutyl)amino)methaniminium (**6b**)

Yellow solid, yield 69%. ^1^H NMR (500 MHz, CD_3_OD) *δ* 7.58–7.51 (m, 3H), 7.41–7.33 (m, 3H), 6.62 (d, *J* = 15.8 Hz, 1H), 3.35 (td, *J* = 6.4, 5.3, 2.9 Hz, 2H), 3.22 (td, *J* = 6.4, 5.4, 3.3 Hz, 2H), 1.64 (h, *J* = 3.7 Hz, 4H). ^13^C NMR (125 MHz, CD_3_OD) *δ* 167.40, 157.26, 140.40, 134.84, 129.48, 128.58, 127.43, 120.37, 40.79, 40.66, 38.40, 26.35, 25.78. HRMS (ESI) C_14_H_21_N_4_O [M + H]^+^ calcd = 261.1710; found [M + H]^+^ = 261.1718.

##### 2.1.4.3 Amino((5-cinnamamidopentyl)amino)methaniminium (**6c**)

Yellow solid, yield 82%. ^1^H NMR (500 MHz, CD_3_OD) *δ* 7.61–7.49 (m, 3H), 7.42–7.33 (m, 3H), 6.62 (d, *J* = 15.7 Hz, 1H), 3.33 (d, *J* = 7.1 Hz, 2H), 3.18 (t, *J* = 7.1 Hz, 2H), 1.67–1.57 (m, 4H), 1.48–1.40 (m, 2H). ^13^C NMR (125 MHz, CD_3_OD) *δ* 167.28, 157.32, 157.27, 140.25, 134.87, 129.45, 128.58, 127.41, 120.49, 41.09, 40.96, 38.87, 38.78, 28.69, 28.60, 28.57, 28.09, 23.60, 23.39. HRMS (ESI) C_15_H_23_N_4_O [M + H]^+^ calcd = 275.1867; found [M + H]^+^ = 275.1874.

#### 2.1.5 General procedure for the preparation of compounds **8a–8h**

Intermediate **2b** (0.11 g, 0.36 mmol), aldehydes **7a–7h** (0.34 mmol) and Et₃N (72.72 mg, 0.72 mmol) were dissolved in methanol (10 mL) and stirred at 0 °C for 4 h to allow imine formation. Then NaBH_4_ (25.70 mg, 0.68 mmol) was added portionwise, and the resulting mixture was allowed to warm to room temperature and stirred for an additional 12–18 h. Upon completion (monitored by TLC), the reaction mixture was concentrated in vacuo, and the residue was extracted with ethyl acetate. The organic phase was washed sequentially with dilute aqueous NaHCO_3_ and brine, dried over anhydrous Na_2_SO_4_, filtered, and evaporated to afford crude intermediates **8a–8h**, which were carried forward without further purification.

#### 2.1.6 General procedure for the preparation of compounds **9a–9h**

Cinnamic acid (1.00 g, 6.75 mmol) was combined with HATU (2.57 g, 6.75 mmol) in anhydrous acetonitrile (20 mL) and stirred at 0 °C for 20 min to effect pre-activation of the carboxyl moiety. DIPEA (1.74 g, 13.50 mmol) and the corresponding amines **8a–8h** (6.75 mmol) were subsequently added, and the reaction mixture was allowed to stir for an additional 5–10 h. Upon confirmation of complete consumption of the starting material by TLC, the solvent was removed. The resulting residue was extracted with ethyl acetate and water. The organic phase was washed sequentially with dilute aqueous HCl and brine, dried over anhydrous Na_2_SO_4_, concentrated in vacuo, and subsequent purification by flash chromatography (dichloromethane/methanol) yielded compounds **9a–9h**, yield 43–77%.

##### 2.1.6.1 N-(4-tert-butyl(diaminomethylene)carbamate-butyl)-N-butylcinnamamide (**9a**)

Yellow oil, yield 52%. ^1^H NMR (500 MHz, CDCl_3_) *δ* 11.50 (s, 1H), 8.37 (d, *J* = 13.4 Hz, 1H), 7.70 (dd, *J* = 15.3, 3.4 Hz, 1H), 7.56–7.48 (m, 2H), 7.41–7.32 (m, 3H), 6.82 (dd, *J* = 15.3, 5.5 Hz, 1H), 3.55–3.35 (m, 6H), 1.62 (ddt, *J* = 23.4, 16.0, 7.8 Hz, 6H), 1.53–1.45 (m, 16H), 1.37 (dq, *J* = 14.8, 7.5 Hz, 2H), 0.96 (dt, *J* = 14.9, 7.4 Hz, 3H). ^13^C NMR (125 MHz, CDCl_3_) *δ* 166.23, 163.54, 156.15, 153.28, 142.46, 135.48, 129.50, 128.91, 128.79, 127.77, 117.64, 117.50, 83.12, 79.41, 53.44, 48.03, 46.41, 40.68, 31.97, 30.09, 28.31, 28.08, 26.67, 25.35, 20.11, 13.86.

##### 2.1.6.2 N-(4-tert-butyl(diaminomethylene)carbamate-butyl)-N-hexylcinnamamide (**9b**)

Yellow oil, yield 77%. ^1^H NMR (500 MHz, CDCl_3_) *δ* 11.50 (s, 1H), 8.36 (dt, *J* = 20.8, 4.2 Hz, 1H), 7.70 (dd, *J* = 15.4, 3.9 Hz, 1H), 7.56–7.47 (m, 2H), 7.37 (qd, *J* = 6.8, 3.2 Hz, 3H), 6.82 (dd, *J* = 15.4, 5.1 Hz, 1H), 3.53–3.33 (m, 6H), 1.71–1.60 (m, 6H), 1.52–1.45 (m, 18H), 1.35–1.29 (m, 6H), 0.90 (q, *J* = 5.4 Hz, 3H). ^13^C NMR (125 MHz, CDCl_3_) *δ* 166.17, 163.65, 156.18, 142.58, 142.39, 135.50, 129.49, 128.79, 127.77, 117.68, 117.52, 83.07, 79.38, 79.25, 48.26, 47.69, 46.97, 46.40, 40.67, 40.20, 31.67, 31.52, 29.84, 28.32, 28.09, 28.03, 27.04, 26.69, 26.56, 26.45, 25.38, 22.61, 14.02.

##### 2.1.6.3 N-(4-tert-butyl(diaminomethylene)carbamate-butyl)-N-octylcinnamamide (**9c**)

Yellow oil, yield 47%. ^1^H NMR (500 MHz, CDCl_3_) *δ* 11.51 (s, 1H), 8.40 (s, 1H), 7.70–7.64 (m, 1H), 7.54–7.50 (m, 2H), 7.40–7.33 (m, 3H), 6.88–6.74 (m, 1H), 3.54–3.36 (m, 6H), 1.63 (d, *J* = 7.2 Hz, 4H), 1.54–1.44 (m, 18H), 1.34–1.26 (m, 12H), 0.89–0.86 (m, 3H). ^13^C NMR (125 MHz, CDCl_3_) *δ* 166.25, 151.78, 151.44, 142.45, 135.48, 129.50, 129.27, 128.79, 127.78, 120.88, 117.65, 117.49, 110.90, 48.25, 47.70, 47.00, 46.37, 31.79, 29.84, 29.45, 29.30, 29.24, 28.28, 28.08, 27.13, 26.87, 26.68, 25.34, 22.63, 14.11.

##### 2.1.6.4 N-decyl-N-(4-tert-butyl(diaminomethylene)carbamate-butyl)cinnamamide (**9d**)

Yellow oil, yield 62%. ^1^H NMR (500 MHz, CDCl_3_) *δ* 7.71 (d, J = 3.8 Hz, 1H), 7.55–7.49 (m, 2H), 7.41–7.32 (m, 3H), 6.82 (dd, *J* = 15.4, 4.7 Hz, 1H), 3.51–3.35 (m, 6H), 1.72–1.58 (m, 6H), 1.47 (d, *J* = 17.3 Hz, 18H), 1.34–1.25 (m, 14H), 0.88 (d, *J* = 6.7 Hz, 3H). ^13^C NMR (125 MHz, CDCl_3_) *δ* 166.16, 163.66, 156.18, 153.30, 142.38, 135.50, 129.48, 128.79, 127.76, 117.69, 83.05, 79.24, 48.23, 47.68, 46.97, 46.38, 40.67, 40.20, 31.88, 31.25, 29.86, 29.58, 29.54, 29.29, 28.32, 28.09, 27.04, 26.88, 26.70, 25.38, 22.68, 14.13.

##### 2.1.6.5 N-(3,6-dimethylhept-5-en-1-yl)-N-(4-guanidinobutyl)cinnamamide (**9e**)

Yellow oil, yield 71%. ^1^H NMR (500 MHz, CDCl_3_) *δ* 11.51 (s, 1H), 8.36 (dt, *J* = 21.5, 5.3 Hz, 1H), 7.70 (dd, *J* = 15.4, 5.3 Hz, 1H), 7.51 (dd, *J* = 7.4, 2.4 Hz, 2H), 7.42–7.32 (m, 3H), 6.82 (dd, *J* = 15.4, 6.3 Hz, 1H), 5.19–5.02 (m, 1H), 3.55–3.34 (m, 6H), 1.99 (ddq, *J* = 30.4, 14.4, 7.9, 7.2 Hz, 2H), 1.77–1.56 (m, 12H), 1.46 (s, 18H), 1.37–1.20 (m, 3H), 0.97 (dd, *J* = 14.0, 6.3 Hz, 3H). ^13^C NMR (125 MHz, CDCl_3_) *δ* 166.11, 163.65, 156.17, 142.46, 135.46, 131.64, 129.50, 128.80, 127.75, 117.57, 83.06, 79.24, 47.61, 46.33, 45.17, 40.69, 40.22, 36.91, 34.84, 30.49, 28.32, 28.30, 26.69, 26.46, 25.75, 25.48, 25.45, 19.56, 17.69.

##### 2.1.6.6 N-(4-tert-butyl(diaminomethylene)carbamate-butyl)-N-(4-(trifluoromethyl)benzyl) cinnamamide (**9f**)

Yellow oil, yield 58%. ^1^H NMR (500 MHz, CDCl_3_) *δ* 11.50 (s, 1H), 8.36 (s, 1H), 7.77 (dd, *J* = 20.8, 15.3 Hz, 1H), 7.57–7.51 (m, 1H), 7.47–7.42 (m, 1H), 7.41–7.28 (m, 4H), 7.24 (d, *J* = 8.5 Hz, 1H), 7.15 (d, *J* = 8.4 Hz, 1H), 6.91–6.83 (m, 3H), 4.64 (d, *J* = 15.1 Hz, 2H), 3.44 (ddt, *J* = 26.1, 19.7, 7.2 Hz, 4H), 1.69–1.57 (m, 4H), 1.52–1.44 (m, 18H). ^13^C NMR (125 MHz, CDCl_3_) *δ* 166.89, 159.15, 153.33, 143.12, 135.30, 129.81, 129.64, 129.55, 128.84, 128.75, 127.85, 127.70, 117.64, 114.34, 113.98, 65.02, 55.32, 50.73, 48.61, 46.57, 46.12, 40.69, 40.19, 38.63, 28.30, 28.09, 26.46, 25.06.

##### 2.1.6.7 N-(4-tert-butyl(diaminomethylene)carbamate-butyl)-N-(pyridin-4-ylmethyl)cinnamamide (**9g**)

Yellow oil, yield 43%. ^1^H NMR (500 MHz, CDCl_3_) *δ* 11.51 (s, 1H), 8.57 (d, *J* = 17.5 Hz, 2H), 8.40–8.26 (m, 1H), 7.83–7.69 (m, 1H), 7.55–7.49 (m, 1H), 7.40–7.27 (m, 4H), 7.17 (d, *J* = 4.9 Hz, 1H), 6.89 (d, *J* = 15.3 Hz, 1H), 4.70 (d, *J* = 13.6 Hz, 2H), 3.45 (dq, *J* = 13.7, 6.9 Hz, 4H), 1.74–1.57 (m, 4H), 1.45 (d, *J* = 9.5 Hz, 18H). ^13^C NMR (125 MHz, CDCl_3_) *δ* 166.96, 163.59, 156.37, 153.42, 150.28, 148.78, 144.42, 135.02, 130.09, 129.00, 128.08, 123.11, 121.51, 116.21, 83.45, 79.56, 49.10, 47.83, 40.04, 31.31, 28.36, 28.15, 28.11, 27.99, 26.66, 26.45.

##### 2.1.6.8 N-(3,5-dibromo-4-methoxybenzyl)-N-(4-tert-butyl(diaminomethylene)carbamate-butyl) cinnamamide (**9h**)

Yellow oil, yield 59%. ^1^H NMR (500 MHz, CDCl_3_) *δ* 11.51 (s, 1H), 8.37 (dt, *J* = 19.9, 5.3 Hz, 1H), 7.79 (t, *J* = 15.8 Hz, 1H), 7.56–7.53 (m, 1H), 7.48–7.42 (m, 2H), 7.41–7.33 (m, 4H), 6.88 (d, *J* = 15.4 Hz, 1H), 4.63 (s, 2H), 3.88 (d, *J* = 8.0 Hz, 3H), 3.46 (dt, *J* = 14.5, 7.3 Hz, 4H), 1.67 (dq, *J* = 36.1, 7.4 Hz, 4H), 1.52–1.45 (m, 18H). ^13^C NMR (125 MHz, CDCl_3_) *δ* 166.67, 163.57, 156.28, 153.36, 144.05, 136.56, 135.07, 131.97, 130.43, 129.88, 128.89, 127.97, 118.30, 116.47, 83.30, 79.41, 60.63, 47.96, 47.09, 40.03, 28.30, 28.10, 28.05, 26.62, 26.43, 24.93.

#### 2.1.7 General procedure for the preparation of compounds **10a–10h**

The synthesis of target compounds **10a**–**10h** from intermediates **9a**–**9h** was carried out via a method comparable to that for **6a**–**6c**, providing yields in the range of 28–56%. It should be noted that for tertiary amide derivatives (compounds **10a–10h**), slow rotation about the amide C-N bond on the NMR timescale may result in the observation of split or broadened signals for certain carbons, particularly for carbonyl and olefinic carbons. Such spectral features arise from the presence of rotamers and do not affect the unambiguous structural assignment, which is further corroborated by HRMS data.

##### 2.1.7.1 Amino((4-(N-butylcinnamamido)butyl)amino)methaniminium (**10a**)

Yellow solid, yield 50%. ^1^H NMR (500 MHz, CD_3_OD) *δ* 7.64–7.56 (m, 3H), 7.39 (qdd, *J* = 7.0, 4.8, 1.9 Hz, 3H), 7.05 (dd, *J* = 15.4, 2.9 Hz, 1H), 3.51 (dddd, *J* = 30.3, 23.1, 15.0, 7.3 Hz, 4H), 3.23 (dt, *J* = 12.4, 6.9 Hz, 2H), 1.74–1.55 (m, 6H), 1.38 (dq, *J* = 20.6, 7.4 Hz, 2H), 0.98 (dt, *J* = 8.6, 7.4 Hz, 3H). ^13^C NMR (125 MHz, CD_3_OD) *δ* 171.33, 161.22, 146.52, 139.01, 133.55, 132.57, 131.54, 121.20, 50.42, 49.66, 44.67, 35.50, 30.43, 29.67, 28.55, 23.82, 23.53, 16.76. HRMS (ESI) C_18_H_29_N_4_O [M + H]^+^ calcd = 317.2336; found [M + H]^+^ = 317.2343.

##### 2.1.7.2 Amino((4-(N-hexylcinnamamido)butyl)amino)methaniminium (**10b**)

Yellow solid, yield 35%. ^1^H NMR (500 MHz, CD_3_OD) *δ* 7.66–7.55 (m, 3H), 7.39 (dt, 3H), 7.06 (dd, *J* = 15.4, 5.8 Hz, 1H), 3.63–3.40 (m, 4H), 3.23 (dt, *J* = 11.4, 6.9 Hz, 2H), 1.77–1.56 (m, 6H), 1.34 (q, *J* = 4.7, 4.2 Hz, 6H), 0.90 (t, *J* = 6.9 Hz, 3H). ^13^C NMR (125 MHz, CD_3_OD) *δ* 167.40, 157.26, 157.24, 142.54, 135.07, 129.62, 128.63, 127.61, 117.31, 45.70, 40.74, 31.27, 29.32, 27.47, 26.43, 26.05, 25.74, 24.62, 22.28, 12.97. HRMS (ESI) C_20_H_33_N_4_O [M + H]^+^ calcd = 345.2649; found [M + H]^+^ = 345.2655.

##### 2.1.7.3 Amino((4-(N-octylcinnamamido)butyl)amino)methaniminium (**10c**)

Yellow solid, yield 28%. ^1^H NMR (500 MHz, CD_3_OD) *δ* 7.68–7.54 (m, 3H), 7.47–7.33 (m, 3H), 7.06 (d, *J* = 15.4 Hz, 1H), 3.63–3.41 (m, 4H), 3.23 (dt, *J* = 13.9, 7.0 Hz, 2H), 1.76–1.55 (m, 6H), 1.38–1.25 (m, 10H), 0.88 (dt, *J* = 13.7, 6.2 Hz, 3H). ^13^C NMR (125 MHz, CD_3_OD) *δ* 167.45, 157.23, 142.56, 135.07, 129.62, 128.61, 127.59, 117.29, 45.66, 40.74, 31.55, 29.29, 28.97, 27.49, 26.75, 26.30, 25.71, 24.61, 22.31, 13.02. HRMS (ESI) C_22_H_37_N_4_O [M + H]^+^ calcd = 373.2962; found [M + H]^+^ = 373.2967.

##### 2.1.7.4 Amino((4-(N-decylcinnamamido)butyl)amino)methaniminium (**10d**)

Pale yellow solid, yield 46%. ^1^H NMR (500 MHz, CD_3_OD) *δ* 7.65–7.56 (m, 3H), 7.44–7.34 (m, 3H), 7.06 (dd, *J* = 15.4, 4.3 Hz, 1H), 3.61–3.40 (m, 4H), 3.23 (dt, *J* = 11.7, 7.0 Hz, 2H), 1.77–1.56 (m, 6H), 1.38–1.23 (m, 14H), 0.88 (dt, *J* = 9.2, 6.9 Hz, 3H). ^13^C NMR (125 MHz, CD_3_OD) *δ* 167.40, 157.25, 142.52, 135.08, 129.61, 128.63, 127.61, 117.33, 45.68, 40.74, 31.66, 29.37, 29.29, 29.06, 29.00, 27.51, 26.76, 26.51, 26.29, 25.74, 24.62, 22.33, 13.09. HRMS (ESI) C_24_H_41_N_4_O [M + H]^+^ calcd = 401.3275; found [M + H]^+^ = 401.3281.

##### 2.1.7.5 Amino((4-(N-(2,6-dimethylhept-5-en-1-yl)cinnamamido)butyl)amino)methaniminium (**10e**)

Pale yellow solid, yield 42%. ^1^H NMR (500 MHz, CD_3_OD) *δ* 7.66–7.55 (m, 3H), 7.43–7.32 (m, 3H), 7.04 (ddd, *J* = 15.5, 5.4 Hz, 1H), 3.65–3.42 (m, 4H), 3.23 (dt, *J* = 13.1, 7.0 Hz, 2H), 2.05–1.07 (m, 20H), 0.98 (qd, *J* = 6.1, 3.5 Hz, 3H). ^13^C NMR (125 MHz, CD_3_OD) *δ* 167.38, 157.25, 142.49, 135.06, 129.66, 128.67, 127.57, 117.99, 117.32, 89.38, 69.98, 43.50, 40.73, 39.90, 34.38, 27.86, 27.79, 26.49, 25.79, 25.73, 21.38, 21.38, 20.74. HRMS (ESI) C_24_H_39_N_4_O [M + H]^+^ calcd = 399.3119; found [M + H]^+^ = 399.3126.

##### 2.1.7.6 Amino((4-(N-(4-(trifluoromethyl)benzyl)cinnamamido)butyl)amino)methaniminium (**10f**)

Pale yellow solid, yield 51%. ^1^H NMR (500 MHz, CD_3_OD) *δ* 7.72–7.62 (m, 4H), 7.54–7.45 (m, 3H), 7.44–7.33 (m, 3H), 7.10 (dd, 1H), 4.94 (s, 1H), 4.81 (s, 1H), 3.57 (dt, *J* = 28.6, 7.3 Hz, 2H), 3.20 (dt, *J* = 24.5, 7.1 Hz, 2H), 1.78–1.55 (m, 4H). ^13^C NMR (125 MHz, CD_3_OD) *δ* 168.14, 167.70, 157.23, 143.67, 142.29, 134.82, 129.78, 128.63, 128.57, 127.82, 127.68, 126.96, 125.45, 125.13, 116.85, 50.47, 48.96, 46.13, 40.70, 26.15, 25.73, 24.40. HRMS (ESI) C_22_H_26_F_3_N_4_O [M + H]^+^ calcd = 419.2054; found [M + H]^+^ = 419.2057.

##### 2.1.7.7 Amino((4-(N-(pyridin-4-ylmethyl)cinnamamido)butyl)amino)methaniminium (**10g**)

Pale yellow solid, yield 56%. ^1^H NMR (400 MHz, CD_3_OD) δ 8.48 (dd, *J* = 15.3, 5.2 Hz, 2H), 7.68–7.60 (m, 2H), 7.52–7.44 (m, 1H), 7.44–7.28 (m, 5H), 7.04 (dd, 1H), 4.75 (s, 2H), 3.57 (dt, 2H), 3.18 (qd, *J* = 7.3, 1.8 Hz, 2H), 1.78–1.50 (m, 4H). ^13^C NMR (100 MHz, CD_3_OD) *δ* 168.24, 167.88, 157.34, 149.12, 148.67, 143.81, 134.99, 129.90, 128.72, 127.92, 127.79, 122.79, 122.09, 116.58, 40.76, 26.32, 25.81, 25.76, 24.53. 26.15, 25.73, 24.40. HRMS (ESI) C_20_H_26_N_5_O [M + H]^+^ calcd = 352.2132; found [M + H]^+^ = 352.2139.

##### 2.1.7.8 Amino((4-(N-(3,5-dibromo-4-methoxybenzyl)cinnamamido)butyl)amino)methaniminium (**10h**)

Pale yellow solid, yield 55%. ^1^H NMR (500 MHz, CD_3_OD) *δ* 7.72–7.61 (m, 2H), 7.54 (d, *J* = 10.5 Hz, 2H), 7.47 (s, 1H), 7.43–7.33 (m, 3H), 7.07 (dd, 1H), 4.72 (d, 2H), 3.82 (d, *J* = 5.7 Hz, 3H), 3.55 (dt, 2H), 3.20 (dt, *J* = 21.5, 7.0 Hz, 2H), 1.78–1.54 (m, 4H). ^13^C NMR (125 MHz, CDCl_3_) *δ* 171.60, 161.17, 157.25, 147.67, 140.93, 138.88, 135.79, 134.76, 133.75, 132.56, 131.76, 122.07, 121.67, 120.51, 63.65, 53.25, 49.96, 44.67, 30.11, 29.66, 28.33. HRMS (ESI) C_22_H_27_Br_2_N_4_O_2_ [M + H]^+^ calcd = 539.0475; found [M + H]^+^ = 539.0483.

### 2.2 *In vitro* antimicrobial assay

The MIC values of cinnamamide derivatives against a panel of strains were determined using the standard microbroth dilution method. Briefly, test compounds were serially diluted two-fold across a 96-well plate, and the wells were subsequently seeded with bacterial cultures in exponential phase, adjusted to a density of 1 × 10^6^ CFU/mL. Positive reference antibiotics (vancomycin and colistin) and a negative control (Mueller-Hinton broth alone) were included. Following incubation at 37 °C for 18–24 h, turbidity was recorded as an indicator of bacterial proliferation. The MIC was defined as the lowest drug concentration that yielded clear, growth-free wells. Each measurement was performed in triplicate or more independent experiments.

### 2.3 Hemolysis activity assay

To evaluate whether **10c** causes red blood cell lysis, a 5% sheep erythrocyte suspension in PBS was prepared. Two-fold dilution series of the compound were placed in 96-well plates, then mixed with the RBC suspension to give final concentrations between 2.5 and 1,280 μg/mL. Following a 1 h incubation at 37 °C, the plates were centrifuged. Supernatant (100 μL) from each well was transferred to a fresh plate, and its absorbance was measured at 540 nm. The percentage of hemolysis was determined as: (*A*_sample − *A*_PBS)/(*A*_Triton X-100 − *A*_PBS) × 100%. Each experiment was carried out in triplicate and repeated three times independently.

### 2.4 Cytotoxicity assay

The cytotoxicity of compound **10c** toward LO2 cells was determined using a CCK-8 assay. In this procedure, LO2 cells were placed into 96-well plates at a concentration of 1 × 10^4^ cells per well and allowed to attach over 24 h. Following exposure to a series of increasing concentrations of compound **10c** for 24 h, the culture medium was removed, and the cells were gently washed with PBS. Subsequently, CCK-8 reagent diluted in fresh culture medium (10%, v/v) was added to each well. The plates were then incubated for 2 h at 37 °C in the dark, after which the absorbance was measured at 450 nm. The percentage of growth inhibition was calculated using the formula: Inhibition rate (%) = [1 − (OD_sample − OD_blank)/(OD_control − OD_blank)] × 100%. All experiments were performed in triplicate and repeated three times independently.

### 2.5 Bactericidal activity in biological fluids

To evaluate the stability of compound **10c** in biological fluids, the minimum bactericidal concentration (MBC) was measured. Briefly, **10c** was preincubated in 50% (v/v) plasma at 37 °C, and MBC values were measured at 0, 3, and 6 h. In parallel, the impact of different biological matrices was investigated by measuring MBCs in 50% plasma, 50% serum, and 50% whole blood. Each assay was performed in triplicate across three independent experimental runs.

### 2.6 Dynamic time-kill curve assay

A single colony of *S. aureus* ATCC 25923 was inoculated into Mueller-Hinton broth and incubated at 37 °C for 5 h. The resulting bacterial culture was then adjusted to a concentration of 1 × 10^6^ CFU/mL. Compound **10c** was added to the bacterial suspension at final concentrations corresponding to 1×, 2×, 4×, and 8× MIC, and the mixtures were incubated at 37 °C. Viable bacterial counts were determined at various time points over a 24 h period (0 to 24 h).

### 2.7 Drug resistance assay

*S. aureus* ATCC25923 was incubated in Mueller-Hinton broth for 5 h at 37 °C, then diluted to 1 × 10^6^ CFU/mL. Compound **10c** was added at a concentration equal to 1/2× MIC (based on the MIC determined on the previous day). The culture was incubated for 18 h, after which the MIC was re-determined. This process was repeated daily for 20 consecutive passages. For each passage, the drug concentration was adjusted to 1/2× of the newly determined MIC from the previous day to maintain consistent selective pressure. MIC values were recorded at each passage to monitor susceptibility changes.

### 2.8 Biofilm inhibition and disruption evaluation

#### 2.8.1 Assessment of biofilm inhibition

A suspension of *S. aureus* ATCC25923 in MHB was prepared at 1 × 10^8^ CFU/mL. In each well, 100 μL of the bacterial suspension was mixed with 100 μL of various concentrations of compound **10c** (0.5–16 μg/mL), then incubated at 37 °C for 24 h. Planktonic bacteria were removed, and the wells were rinsed three times with PBS. Adherent biofilm was fixed using 150 μL of methanol (30 min incubation). Following aspiration of methanol, the biofilm was stained with 150 μL of 0.1% crystal violet for 15 min, then washed three times with PBS. The crystal violet was solubilized by adding 150 μL of ethanol, and the optical density was recorded at 575 nm. The relative biofilm mass was expressed as (OD_sample − OD_blank)/(OD_positive control − OD_blank) × 100%.

#### 2.8.2 Assessment of biofilm disruption

To evaluate biofilm disruption, *S. aureus* ATCC25923 was first allowed to form biofilms in 96-well plates for 24 h. Following the removal of non-adherent bacteria, the biofilms were treated with six concentration levels of compound **10c** (0.5× to 16× MIC) for 24 h at 37 °C. The crystal violet assay (absorbance at 575 nm) was used to measure the remaining biofilm mass. The percentage of biofilm mass was then determined by the formula: (OD_sample − OD_blank)/(OD_positive control − OD_blank) × 100%.

### 2.9 Zeta potential assay

#### 2.9.1 Bacterial zeta potential test

Log-phase *S. aureus* ATCC 25923 was harvested, washed three times with 40 mM HEPES (pH 7.4), and resuspended to 1 × 10^8^ CFU/mL. Compound **10c** was introduced at 0–64 μg/mL, and the mixtures were incubated at 37 °C for 8 h, followed by a 1 h rest at room temperature. Zeta potential was then measured.

#### 2.9.2 Cellular zeta potential test

HEK293T cells were seeded at a density of 5 × 10^5^ per 5 cm dish and cultured at 37 °C under 5% CO₂ for 12 h to ensure full attachment. Afterwards, the culture medium was replaced with fresh medium containing different concentrations of compound **10c**, and the cells were incubated for an additional 2 h. Following three washes with PBS, the cells were harvested using 0.25% trypsin-EDTA, collected by centrifugation, and then resuspended in 40 mM HEPES buffer (pH 7.4) for zeta potential analysis.

### 2.10 Membrane depolarization assay

*S. aureus* ATCC25923 in mid-logarithmic growth were pelleted, washed thrice with PBS, and resuspended to 1.0 × 10^8^ CFU/mL. A 150 μL portion of the suspension was placed into a black 96-well plate, mixed with 50 μL of 10 μM DiSC_3_(5), and incubated in the dark for 30 min. Baseline fluorescence was then read at 2-min intervals for 6 min (622/670 nm). Following addition of compound **10c** (1×, 2×, 4×, 8× MIC), fluorescence readings continued for 14 min.

### 2.11 Membrane permeabilization assay

Mid-log cultures of *S. aureus* ATCC25923 were washed three times with PBS and diluted to 1.0 × 10^8^ CFU/mL. Then, 150 μL of the bacterial suspension was placed into each well of a black 96-well plate, combined with 50 μL of 10 μM PI, and incubated in the dark for 30 min. Initial fluorescence was read at 2-min intervals for 6 min (535/615 nm). Subsequently, compound **10c** (1–8 μg/mL) was added, and fluorescence was tracked for an additional 14 min.

### 2.12 DAPI/PI fluorescence assay

Samples of *S. aureus* ATCC25923 harvested during mid-exponential growth were incubated with **10c** at 4× MIC over a 2-h period. After centrifugation at 4000 rpm for 5 min, the supernatant was removed, the pellet was washed, and the cells were resuspended in 160 μL PBS to a density of 1 × 10^8^ CFU/mL. Next, 20 μL of PI (10 μg/mL) together with 20 μL of DAPI (10 μg/mL) were added, and the mixture was incubated at 4 °C in the absence of light for 30 min. An aliquot of 10–20 μL was then deposited onto a chamber slide and visualized with a laser scanning confocal microscope.

### 2.13 ROS determination

Mid-logarithmic phase cultures of *S. aureus* ATCC25923 were collected, resuspended in PBS, and adjusted to a concentration of 1.0 × 10^8^ CFU/mL. An equal volume of 10.0 μM DCF-DA was added, and the mixture was incubated at 37 °C for 30 min. After centrifugation at 3500 rpm for 4 min, the cells were washed and resuspended in PBS. Aliquots were then transferred to a black 96-well plate and treated with compound **10c** at concentrations ranging from 2× to 16× MIC for 1 h in the dark. Fluorescence intensity was measured at excitation/emission wavelengths of 488/530 nm.

### 2.14 Protein and DNA leakage assay

*S. aureus* ATCC25923 cultures in mid-log growth phase were resuspended in PBS to reach 1.0 × 10^8^ CFU/mL. After being treated with compound **10c** (2×–16× MIC) for 4 h at 37 °C, the reaction mixtures were centrifuged at 3500 rpm for 4 min. A BCA assay kit was used to evaluate the protein content, and a micro-spectrophotometer was employed to quantify the DNA present in the supernatant.

### 2.15 Effect of bacterial membrane components

Serial two-fold dilutions of each bacterial membrane component (PG, PE, CL, and PGN) were made in sterile water to give final concentrations of 2–64 μg/mL. Compound 10c was separately dissolved in sterile water to a concentration of 512 μg/mL. The diluted components and 10c were combined in a 96-well plate, after which 100 μL of *S. aureus* ATCC25923 inoculum (1 × 10^6^ CFU/mL) was added. The plate was incubated at 37 °C for 18 h, and then the MIC values were read.

### 2.16 ITC assay

ITC experiments were performed to assess the binding affinity between compound **10c** and PG. PG (2 mM) and **10c** (0.2 mM) were dissolved in a solution of 5% DMSO in sterile water. The titration consisted of 25 consecutive injections of PG into the ITC cell containing **10c**, with a 120-s pause following each injection. The software provided by the instrument calculated the *K_D_*, Δ*H*, and Δ*S* parameters.

### 2.17 *In vivo* antibacterial activity

The *in vivo* efficacy of compound **10c** was evaluated in a mouse skin abscess model induced by *S. aureus* ATCC25923. Animal experiments were performed in accordance with the guidelines approved by the Animal Care and Use Committee of Liaocheng University. Male BALB/c mice (6–8 weeks old, ~20 g) were obtained from Shandong Pengyue Experimental Animal Technology Co., Ltd. [License Number: SCXK(LU)20220006]. Abscesses were induced in SPF-grade BALB/c mice by subcutaneous injection of a *S. aureus* ATCC25923 suspension at a concentration of 6 × 10^8^ CFU/mL, with the mice first anesthetized using isoflurane inhalation: induction was performed with 3% isoflurane in oxygen (1 L/min flow rate) in an induction chamber until loss of the righting reflex (approximately 3–5 min), followed by maintenance with 1.5–2% isoflurane via a nose cone during the injection procedure. Following infection, the animals were randomly assigned into five groups (*n* = 5 each): a control receiving PBS, an untreated infection group, a vancomycin-treated group (10 mg/kg), and two groups treated with **10c** at either 5 mg/kg or 10 mg/kg. Two hours after bacterial challenge, the corresponding treatment (100 μL) was administered via local subcutaneous injection around the abscess sites. All injections were repeated every 12 h over a period of 2 days, and the progression of abscess appearance was further monitored over a 24 h period. After that, the mice were euthanized by isoflurane overdose (5% isoflurane in oxygen at 1 L/min for 3–5 min, followed by cervical dislocation to ensure death), and the infected skin tissues were harvested. Skin samples from the infected area were homogenized in sterile saline, serially diluted, and then spread onto MHA agar plates. Following incubation at 37 °C for 16–24 h, bacterial colonies were counted. For histological evaluation, the collected tissues were fixed, embedded in paraffin, sectioned, and stained with hematoxylin and eosin (H&E). Pathological changes were observed under a digital microscope.

## 3 Results and discussion

### 3.1 Chemistry

**Scheme 1:**
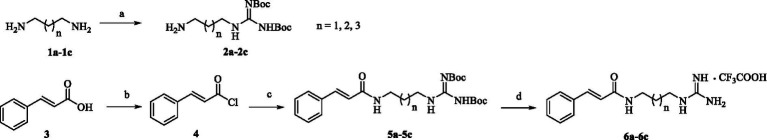
Reagents and conditions: (a) 1,3-Di-Boc-2-methylisothiourea, DIPEA, dichloromethane, rt., 3 h; (b) Thionyl chloride, DMF, reflux, 2 h; (C) **2a–2c**, Et_3_N, dichloromethane, 0 °C, 10 h; (D) CF_3_COOH, dichloromethane, rt., 10–18 h.

The synthetic routes to **6a–6c** are presented in Scheme 1. The synthesis commenced with the installation of a Boc-protected guanidine moiety. The starting material was treated with 1,3-di-Boc-2-methylisothiourea in the presence of DIPEA in dichloromethane at room temperature for 3 h to afford intermediates **2a**–**2c**. Subsequently, cinnamic acid was activated using thionyl chloride with a catalytic amount of DMF under reflux for 2 h, followed by base-mediated amidation to yield **5a**–**5c**. Finally, global deprotection of the Boc groups was achieved with trifluoroacetic acid in dichloromethane, furnishing the guanidinium salts **6a**–**6c**.

**Scheme 2:**
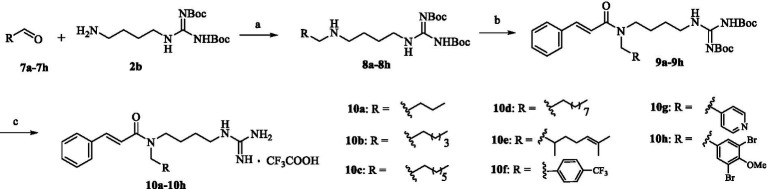
Reagents and conditions: (a) (i) Et_3_N, Methanol, 0 °C, 4 h; (ii) NaBH_4_, 0 °C, 12–18 h; (b) Cinnamic acid, HATU, DIPEA, acetonitrile, rt., 5–10 h; (c) CF_3_COOH, dichloromethane, rt., 10–18 h.

The target guanidinium-containing products **10a–10h** were synthesized via a convergent route, as outlined in Scheme 2. The strategy employed a reductive amination sequence to construct the key amine linkage. Specifically, aldehydes **7a–7h** were condensed with amine **2b** to form the corresponding imine intermediates, which were subsequently reduced with NaBH_4_ to afford secondary amines **8a**–**8h**. These intermediates were then coupled with cinnamic acid using HATU as the coupling reagent and DIPEA as the base in acetonitrile, yielding the Boc-protected amides **9a**–**9h**. Finally, global deprotection of the Boc groups was achieved with TFA, providing the desired final products **10a**–**10h**.

### 3.2 *In vitro* antimicrobial activities

Minimum inhibitory concentrations (MICs) were established through the routine broth microdilution method as part of our *in vitro* antibacterial evaluation. A series of guanidinium-containing compounds (**6a–6c** and **10a–10h**) were evaluated for their in vitro antibacterial activity against a panel of seven bacterial strains, including Gram-positive strains (*S. aureus* ATCC25923 and ATCC43300, *E. faecalis* ATCC29212, *B. subtilis* ATCC9372) as well as Gram-negative (*K. pneumoniae* ATCC10031, *E.coli* ATCC25922, *P. aeruginosa* ATCC27853) pathogens. Vancomycin and colistin were employed as control drugs.

To assess the impact of varying the linker length (*n*) on antibacterial properties, compounds **6a**–**6c** were synthesized. These derivatives shared a common cinnamoyl guanidinium scaffold, in which the cinnamoyl unit was connected to the guanidine via an amide bond. As summarized in Table 1, all three compounds displayed uniformly weak to moderate activity, with MIC values ranging from 16 to 128 μg/mL against Gram-positive strains and ≥128 μg/mL against most Gram-negative pathogens. Among them, **6b** (*n* = 2) exhibited the highest potency (MIC = 16 μg/mL against *S. aureus* ATCC25923 and 32 μg/mL against MRSA ATCC43300), whereas **6a** (*n* = 1) and **6c** (*n* = 3) were less effective (MIC ≥64 μg/mL). None of the compounds showed meaningful activity against *K. pneumoniae*, *E. coli*, or *P. aeruginosa* (MIC >128 μg/mL). These results indicated that the optimal antibacterial activity was achieved when the intermediate linking chain contained four carbon atoms (*n* = 2). The narrow spectrum and generally weak antibacterial activity observed might stem from inadequate hydrophobicity, which likely prevented these lipid chains from inserting effectively into the bacterial membrane bilayer. On the basis of this hypothesis, we kept the optimal four-carbon linker in the subsequent structural optimization and focused on modifying the hydrophobic region. To systematically adjust the overall amphiphilic properties, various groups such as linear alkyl, unsaturated alkyl, and aromatic chains were introduced onto the cinnamamide scaffold.

**Table 1:** In vitro antibacterial activities of cinnamic acid-guanidinium derivatives.

Comp.	MIC (μg/mL)						
	*S. aureus*	*S. aureus*	*E. faecalis*	*B. subtilis*	*K. pneumonia*	*E. coli*	*P. aeruginosa*
	25,923	43,300	29,212	9,372	10,031	25,922	27,853
**6a**	64	64	32	64	>128	>128	>128
**6b**	16	32	32	32	128	>128	>128
**6c**	64	64	128	32	>128	>128	>128
**10a**	32	64	16	32	128	>128	>128
**10b**	4	8	32	16	32	128	32
**10c**	1	2	2	1	8	16	32
**10d**	2	4	4	8	16	64	32
**10e**	4	16	32	4	32	64	128
**10f**	4	8	32	8	32	64	128
**10g**	8	8	32	16	32	32	64
**10 h**	2	8	32	8	16	64	64
**Van**	1	1	2	1	128	128	>128
**Cos**	64	128	>128	16	2	1	1

*S. aureus* 25,923: *Staphylococcus aureus* ATCC25923, erythromycin-susceptible strain; *S. aureus* 43,300: *Staphylococcus aureus* ATCC43300, methicillin-resistant strain; *E. faecalis* 29,212: *Enterococcus faecalis* ATCC29212, vancomycin-susceptible strain; *B. subtilis* 9,372; *Bacillus subtilis* ATCC9372, penicillin-susceptible strain; *K. pneumonia* 10,031: *Klebsiella pneumoniae* ATCC10031; *E. coli* 25,922: *Escherichia coli* ATCC25922, penicillin-susceptible strain; *P. aeruginosa* 27,853: *Pseudomonas aeruginosa* ATCC27853, penicillin-susceptible strain; Van, vancomycin; Cos, colistin; All antimicrobial assays were performed in three independent replicates.

In order to clarify the impact of side-chain hydrophobicity on activity modulation, we synthesized compounds **10a–10h**. The reductive amination products **10a–10h** incorporated a hydrophobic group on the amide nitrogen, creating a secondary-amine-containing linker. This structural modification led to a dramatic improvement in activity, with pronounced structure-dependent variations. For the series of compounds bearing linear alkyl tails (**10a–10d**), a distinct structure–activity relationship (SAR) emerged: the anti-Gram-positive efficacy showed a bell-shaped dependence on carbon chain length. For instance, compound **10a** (butyl chain) exhibited weak to moderate antibacterial action, with MIC values between 16 and 64 μg/mL. Replacing the butyl chain with a hexyl group (**10b**) resulted in substantially improved effect against Gram-positive strains (MICs = 4–32 μg/mL). Optimal performance was associated with an eight-carbon chain (**10c**, MICs = 1-2 μg/mL), defining a favorable hydrophobicity property. However, when the chain was further elongated to a decanyl unit (**10d**), a pronounced loss of activity was observed (MICs = 2–8 μg/mL), indicating that excessive hydrophobicity was unfavorable. Furthermore, substituting the flexible alkyl chains with bulky, conformationally restricted groups such as citronellyl (**10e**), p-trifluoromethylbenzyl(**10f**), methylenepyridyl (**10g**) and 3,5-dibromo-4-methoxybenzyl (**10h**) resulted in moderate improved activity (MICs = 2–32 μg/mL) relative to **6a**–**6c**. Among them, **10c** emerged as the most potent analog, displaying MIC values of 1-2 μg/mL against all four Gram-positive strains, and moderate activity against *K. pneumoniae* (8 μg/mL), *E. coli* (16 μg/mL), and *P. aeruginosa* (32 μg/mL). **10d** followed closely against Gram-positive bacteria with MIC value of 2–8 μg/mL. These results indicated that all compounds in this series exhibited a clear preference for Gram-positive bacteria over Gram-negative ones. This marked selectivity of these cinnamamide derivatives could be explained by key structural and compositional differences between the two classes of bacteria. More specifically, Gram-negative bacteria are equipped with an extra outer membrane (OM) that serves as an efficient barrier to penetration. In contrast, Gram-positive bacteria have no such outer membrane, allowing our membrane-active agents to gain access to and incorporate into the cytoplasmic membrane with much greater ease. This narrow-spectrum preference for these guanidinium derivatives against Gram-negative bacteria, however, did not represent an insurmountable limitation. Future optimization efforts could focus on enhancing the self-promoted uptake mechanism by increasing the overall amphiphilicity or positive charge density through structural modifications, such as the introduction of oligo-guanidinium head groups or hydrophobic tail elongation. Alternatively, combination with sub-MIC levels of membrane permeabilizers (e.g., EDTA or polymyxin B derivatives) may synergistically disrupt the outer membrane integrity, thereby expanding the antibacterial spectrum. These strategies offer rational avenues for the development of broader-spectrum analogs in subsequent drug discovery campaigns.

In summary, a series of cinnamic acid-guanidinium analogs was prepared and tested for antibacterial efficacy. SAR studies highlighted the importance of linker length and amphiphilicity for antimicrobial activity. An octyl chain was identified as optimal for combating Gram-positive bacteria, and the corresponding analog **10c** showed the best activity, with broad-spectrum activity against Gram-positive strains. Therefore, **10c** served as a promising lead warranting further *in vitro* and *in vivo* investigation.

### 3.3 *In vitro* hemolysis and cytotoxicity

**Figure 2:**
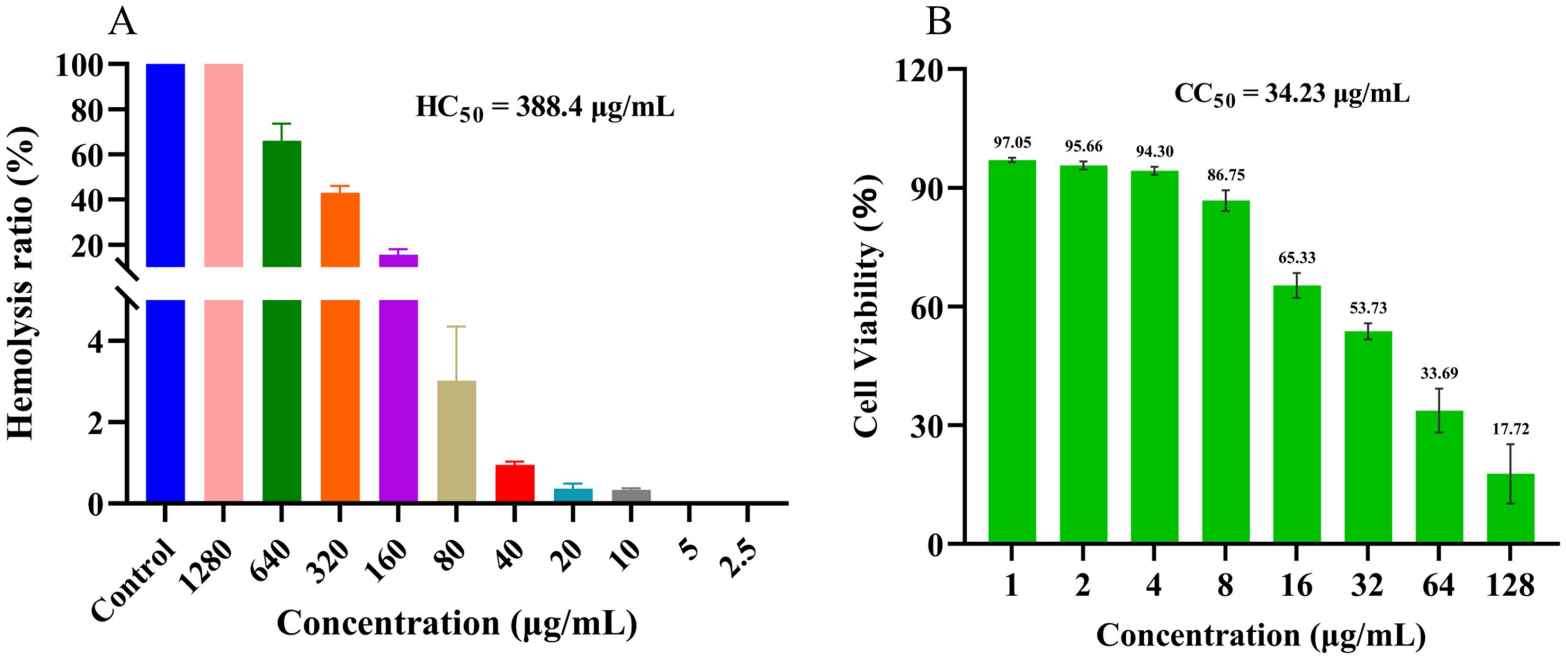
Assessment of the safety profile of compound **10c**. **(A)** Hemolytic toxicity; **(B)** Cytotoxicity against LO2 cells determined by the CCK-8 assay. Data are expressed as mean ± SD (*n* = 3 biological replicates).

The safety evaluation of the most potent derivative, **10c**, was carried out using hemolysis and cytotoxicity tests. Mature red blood cells, which are enclosed only by a plasma membrane, serve as a straightforward model to evaluate membrane-disruptive effects, where the release of hemoglobin indicates hemolytic toxicity (Guo et al., 2021). As shown in Figure 2A, **10c** induced very low hemolytic activity, with an HC_50_ value of 388.4 μg/mL. Notably, even at a relatively high concentration of 40 μg/mL, the observed hemolysis rate was merely 1.54%, underscoring its excellent membrane compatibility over a broad concentration range. To further examine its biological safety, **10c** was further tested against LO2 cells. Compound **10c** demonstrated weak cytotoxic effects, yielding a CC_50_ of 34.23 μg/mL (Figure 2B). Importantly, even at levels well above the MIC range, cell viability stayed notably high (65.33% at 16 μg/mL). To further quantitatively assess the therapeutic window of compound **10c**, its selectivity index (SI) was determined. Using the CC_50_ value against LO2 cells (34.23 μg/mL) and the MIC against *S. aureus* ATCC25923 (1 μg/mL), the SI (defined as CC_50_/MIC) was calculated as 34.23, which points to a favorable safety margin. In addition, the hemolytic selectivity index (HC_50_/MIC) reached 388.4, further underscoring its outstanding ability to distinguish bacterial cells from mammalian cell membranes. Taken together, these results indicated that **10c** demonstrated a good safety profile, showing preferential activity against bacterial cells rather than mammalian cells, which offers a promising therapeutic window for future development.

### 3.4 Stability in blood

**Figure 3:**
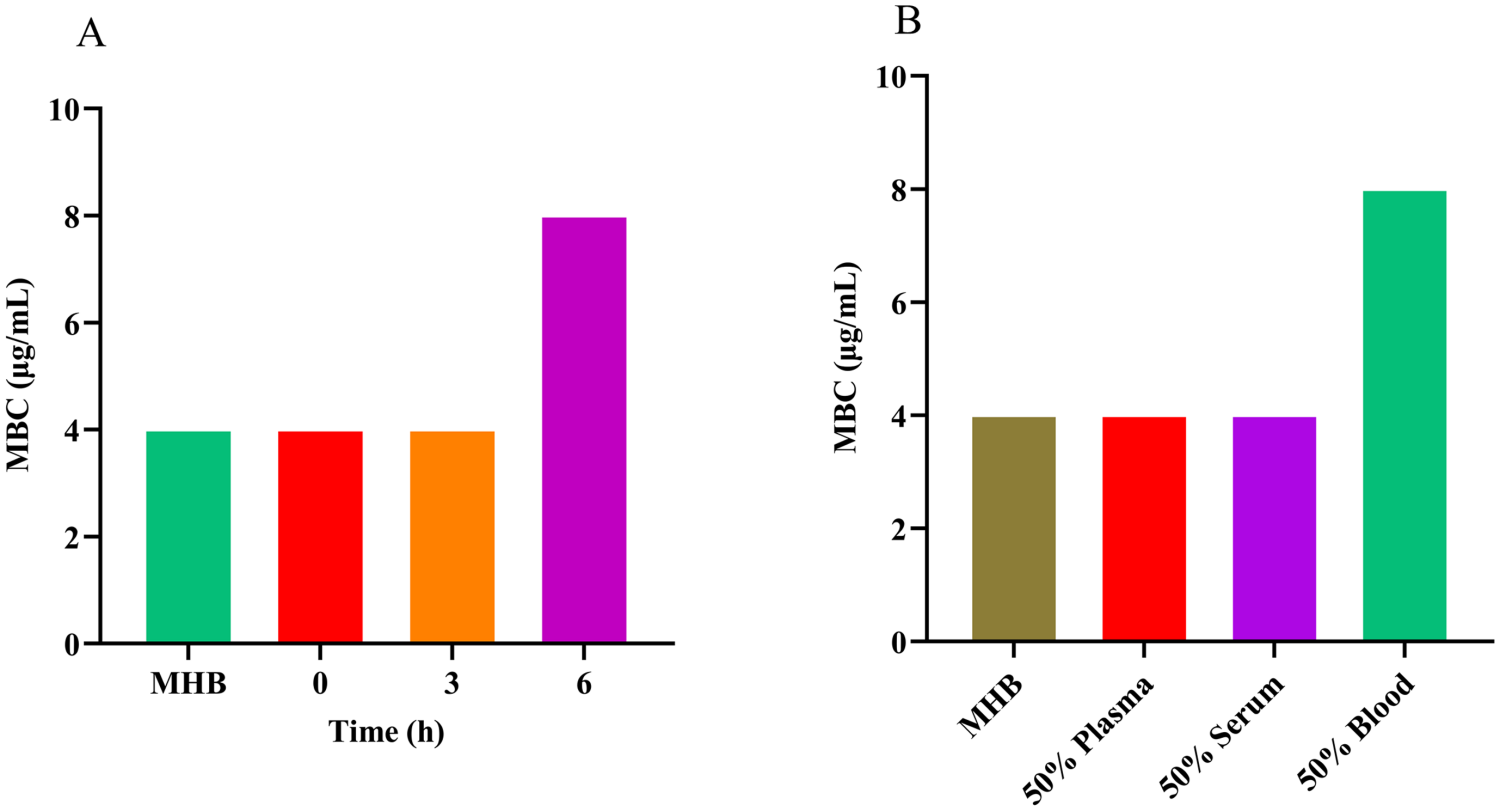
Analysis of compound **10c**’s stability and bactericidal performance in complex biological surroundings. **(A)** Stability in plasma was tested by incubating the compound in 50% human plasma for 0, 3, and 6 h, followed by MBC determination against *S. aureus*. **(B)** To examine how various blood-based matrices affect activity, MBC values were measured in different physiological fluids, a ≤2-fold change of MBC relative to the control was considered as good stability (no significant loss of activity); a 2- to 4-fold change was defined as moderate reduction; and a ≥4-fold change was defined as significant loss of bactericidal activity.

The stability of compound **10c** in physiological fluids is a key factor influencing its effectiveness and pharmacokinetic behavior (Muller and Milton, 2012). The sensitivity of **10c** to enzymatic breakdown was evaluated by pre-incubating the compound in 50% sheep plasma and whole blood, followed by monitoring changes in its minimum bactericidal concentration (MBC) over time. As depicted in Figure 3A, Exposure of **10c** to 50% plasma for up to 3 h did not altered its bactericidal activity against *S. aureus* ATCC25923, which was comparable to the values in control Mueller-Hinton broth. After incubation for 6 h, the MBC of **10c** stayed near its starting level, with an increase of no more than two times, indicating good stability in plasma. Additionally, compound **10c** retained considerable bactericidal potency when assessed in various complex mammalian matrices, including 50% plasma, 50% serum, and 50% whole blood (Figure 3B). Of note, the MBC values observed in these biological environments increased by no more than twofold relative to control conditions, confirming that **10c** preserved its structural integrity and biological function even in the presence of blood components. Such exceptional stability overcame a major limitation commonly associated with conventional AMPs and further supported the suitability of **10c** for systemic therapeutic applications.

### 3.5 Bactericidal time-kill kinetics

**Figure 4:**
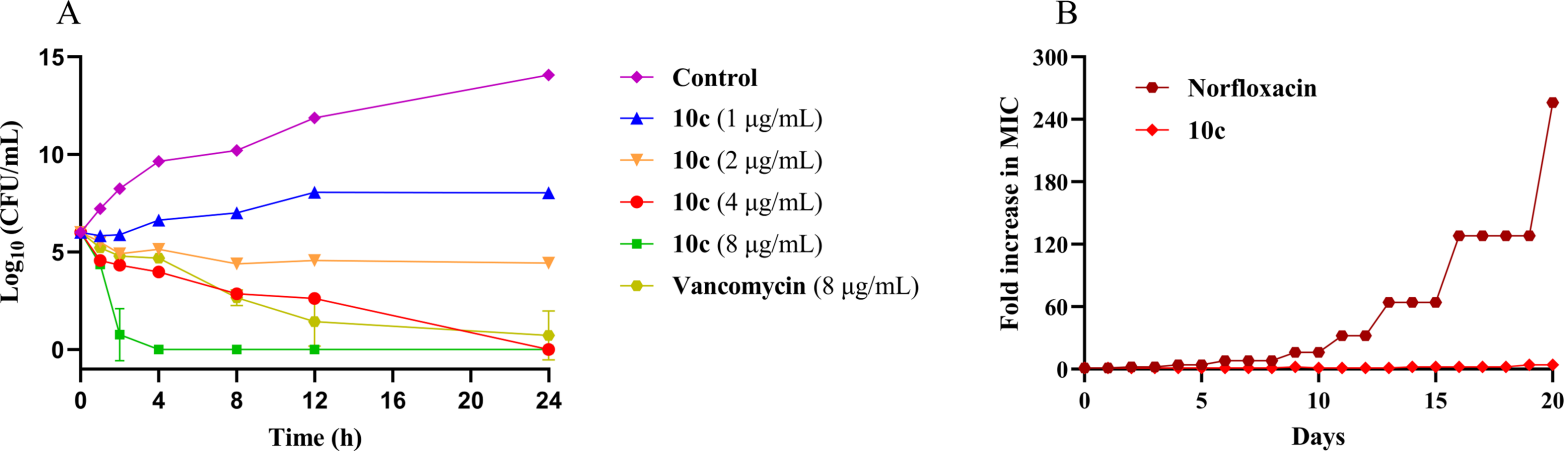
*In vitro* antimicrobial profiling of compound **10c**. **(A)** Time-kill curves depicting the bactericidal dynamics of **10c** against *S. aureus* at varying concentrations. **(B)** Longitudinal assessment of adaptive resistance development, wherein *S. aureus* was subjected to repeated sub-culturing in the presence of sub-MIC concentrations of compound **10c** to monitor any attenuation of susceptibility.

In order to better understand the pharmacodynamic behavior of **10c**, its bactericidal effect over time was tested against *S. aureus* ATCC25923, employing vancomycin as a reference standard. As shown in Figure 4A, compound **10c** displayed rapid, concentration-dependent killing. At lower concentrations (1 and 2 μg/mL), the compound mainly inhibited bacterial proliferation, producing a bacteriostatic effect. In contrast, when the concentration was raised to 4× MIC, a marked reduction in viable cell numbers was observed, leading to a >9 log_10_ decline (≥99.99% killing) within 12 h relative to the untreated control. Notably, at 8× MIC (8 μg/mL), **10c** achieved complete elimination of the initial bacterial inoculum in only 2 h. By comparison, vancomycin at the same concentration failed to eradicate the bacteria entirely over the entire 24 h observation period. These findings indicated that **10c** possessed potent, fast-acting bactericidal activity, surpassing the reference antibiotic vancomycin in both the speed and the extent of bacterial killing.

### 3.6 Resistance development evaluation

The likelihood of inducing bacterial resistance represents a key consideration when evaluating new antibacterial candidates, especially for membrane-active agents that are theoretically expected to delay the emergence of resistance (Waclaw, 2016). To investigate this attribute, we conducted 20 serial passages of *S. aureus* ATCC25923 at sub-MIC levels (0.5 × MIC) of **10c**, with the drug concentration adjusted daily based on the MIC value determined from the preceding passage. Each passage was incubated for 18 h before MIC determination. As illustrated in Figure 4B, the MIC of **10c** remained largely unaltered across the whole passaging procedure, showing no more than a fourfold variation from the initial value. This sustained susceptibility was consistent with the anticipated low resistance development associated with membrane-targeting mechanisms. In sharp contrast, norfloxacin exhibited a rapid decline in effectiveness, with MIC values increasing up to 256-fold after 20 passages. Collectively, these comparative results demonstrated that **10c** posed a substantially higher barrier to resistance development than conventional antibiotics.

### 3.7 Antibiofilm activity studies

**Figure 5:**
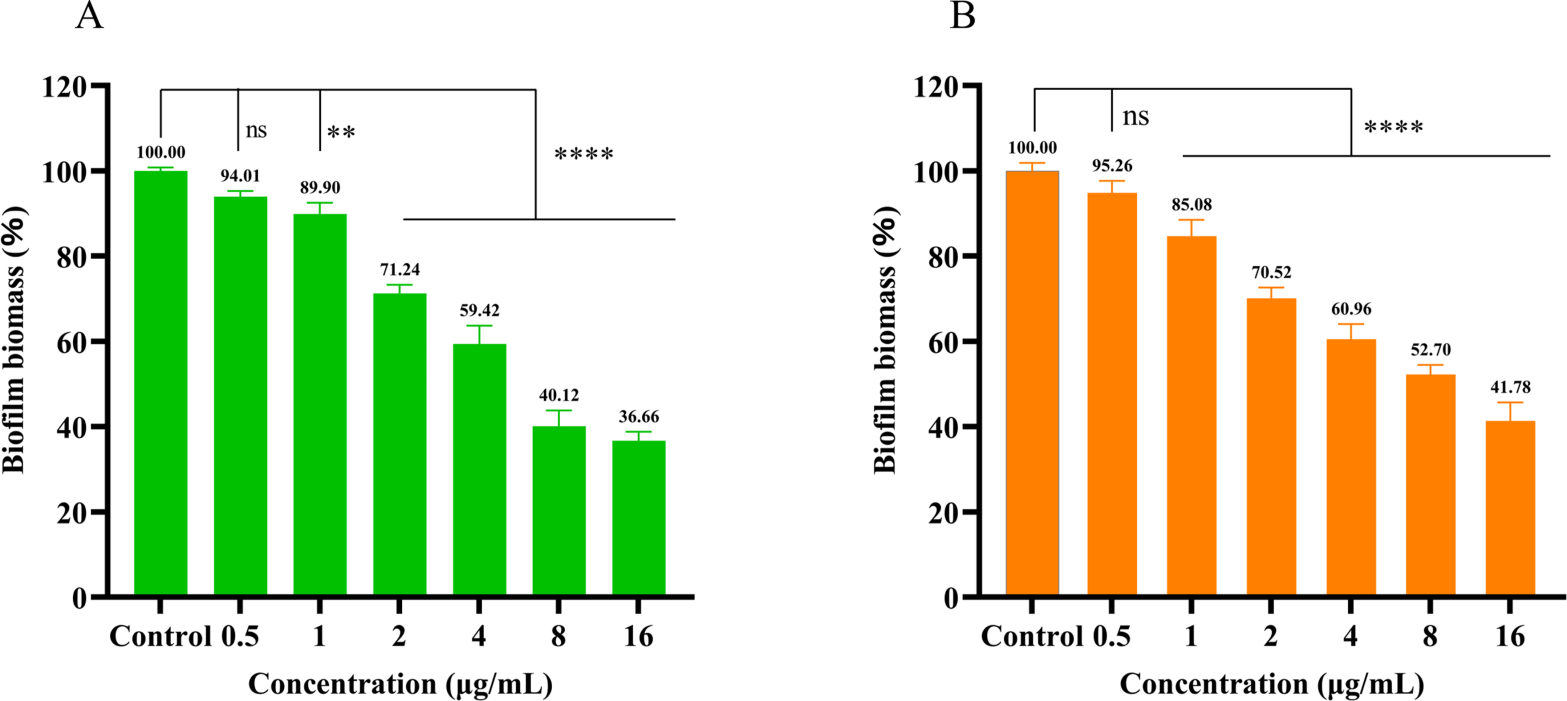
Anti-biofilm efficacy of **10c** against *S. aureus*. **(A)** The capacity of **10c** to inhibit biofilm development was examined across a range of concentrations, with biomass formation subsequently quantified. **(B)** The disruptive potential of **10c** against established, mature biofilm architectures was assessed via crystal violet staining, which measures residual adherent biomass. Values represent the mean ± SD of three biological replicates. Statistical comparisons were conducted by one-way ANOVA test (****p < 0.0001 vs. the control group).

Under nutrient-rich and favorable environmental conditions, bacteria tend to proliferate rapidly via quorum-sensing mechanisms and differentiate into highly organized, structured communities known as biofilms (Su et al., 2022). Among various species, *S. aureus* exhibits a particularly potent ability to construct biofilms. This complex extracellular polymeric matrix not only can drastically enhance bacterial tolerance to antibiotics, but also serves as a physical diffusion barrier and facilitates immune evasion, consequently weakening the host’s immune response. Therefore, the effective inhibition and disruption of biofilm architecture is regarded as a key strategy for achieving sustained bactericidal efficacy. To determine the anti-biofilm potential of **10c** against *S. aureus* ATCC25923, the crystal violet staining method was employed. As shown in Figure 5A, **10c** suppressed biofilm formation in a dose-dependent manner. When applied at 4 μg/mL, **10c** decreased biofilm biomass by 40.58%, and at the higher concentration of 16 μg/mL, the inhibitory effect became even more pronounced, reducing biofilm by 63.34%. In addition, the ability of **10c** to eliminate pre-formed biofilms was examined by exposing 24 h mature biofilms to the compound for another 24 h (Figure 5B). A clear dose-dependent eradicative effect was observed, with modest disruption at lower doses and a maximum of 58.22% removal of the established biomass at the highest concentration tested (16 μg/mL). Together, the above data demonstrated that **10c** possessed the dual activity to inhibit biofilm development and eradicate established biofilms, thereby enhancing its antibacterial efficacy.

### 3.8 Antimicrobial mechanism studies

Since the design of these cinnamamide derivatives drew inspiration from the structural characteristics of cationic AMPs, we hypothesized that their antibacterial activity was primarily mediated through a membrane-disruptive mechanism. To systematically test this hypothesis, a tiered mechanistic framework was established using the active lead compound **10c**, encompassing three key levels. (i) Initial interaction event: a MIC shift assay combined with isothermal titration calorimetry (ITC) was used to confirm the specific electrostatic interaction between **10c** and anionic lipid components in the bacterial cell membrane. (ii) Intermediate effect characterization: surface potential measurement, membrane depolarization assays, and permeability probe techniques were further applied to quantitatively assess, from multiple dimensions, the loss of membrane structural integrity. (iii) Downstream effect validation: ROS levels and leakage of intracellular contents were analyzed to systematically investigate secondary biochemical events triggered by membrane disruption, such as oxidative stress and cytoplasmic content release. This progressive framework, following the sequence of electrostatic binding, membrane structural damage, and cellular functional collapse, was designed to elucidate whether **10c** exerted its bactericidal action by targeting the cell membrane, thereby providing a clear mechanistic basis for subsequent structural optimization.

#### 3.8.1 Binding to bacterial membrane structural elements

**Figure 6:**
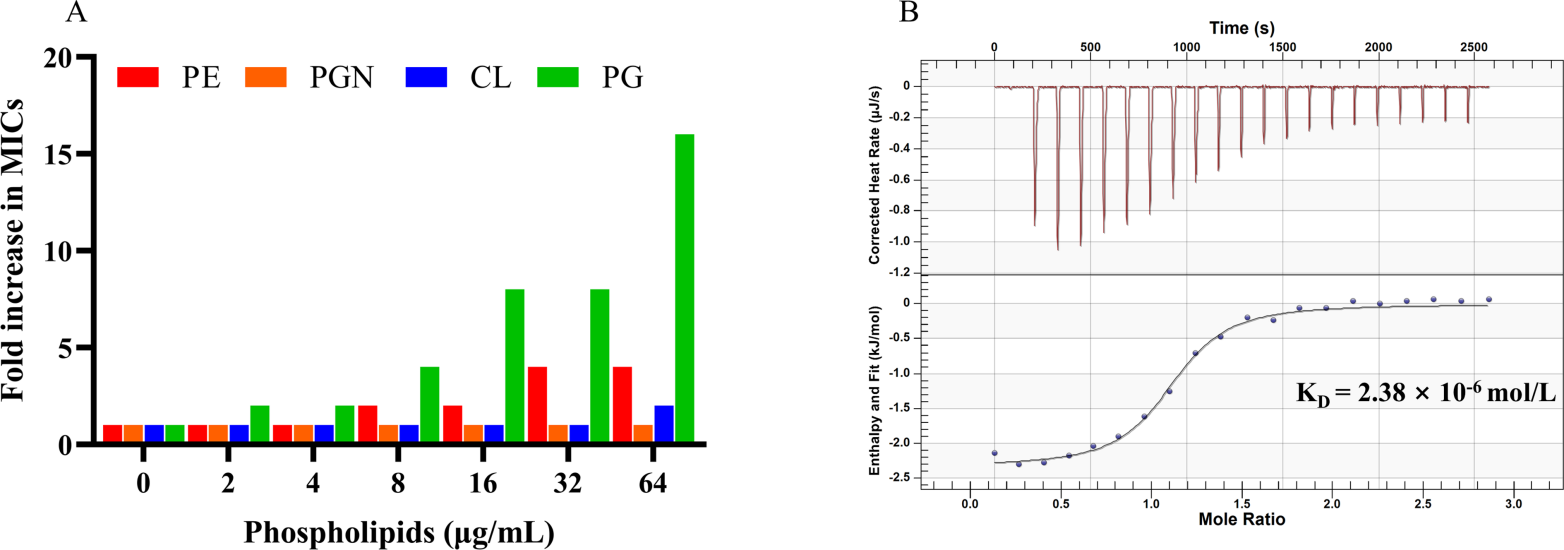
Elucidation of the antimicrobial mechanism of **10c** against *S. aureus*. **(A)** MIC assays for **10c** were conducted with exogenous supplementation of various lipids (PE, PGN, PG, and CL) over a concentration gradient of 0–64 μg/mL. **(B)** ITC-based quantification of the binding between **10c** and PG. The interaction showed a K_D_ value of 2.38 × 10^−6^ mol·L^−1^ and a binding site number (*n*) close to unity (1.064). The binding was associated with a negative Δ*H* (−22.334 kJ·mol^−1^) and a positive Δ*S* (99.85 J·mol^−1^·K^−1^), suggesting that the binding is favored by both enthalpy and entropy.

To gain deeper insight into the molecular basis of **10c**’s selective antibacterial action, a systematic evaluation was carried out to determine how the antimicrobial effectiveness against *S. aureus* ATCC25923 is affected by the presence of added bacterial membrane elements such as phosphatidylethanolamine (PE), phosphatidylglycerol (PG), cardiolipin (CL), and peptidoglycan (PGN). As shown in Figure 6A, the antibacterial activity of **10c** remained unchanged upon addition of PGN. Only minor alterations in potency were observed when PE or CL was added. In sharp contrast, the addition of PG caused a concentration-dependent and marked reduction in the activity of **10c**. At a PG concentration of 64 μg/mL, the MIC value rose by as much as 16-fold compared to the control. The observed differences imply that PG-specific recognition and binding played a role in the bactericidal action of **10c**. The fact that this negatively charged phospholipid was richly distributed in bacterial cell membranes while being nearly absent from mammalian membranes gave rise to a critical structural rationale underlying the selective bacterial killing by **10c**. Furthermore, ITC assay was used to quantitatively characterize the **10c**-PG binding. The results yielded a dissociation constant (*K_D_*) of 2.38 × 10^−6^ mol/L (Figure 6B), indicating a high-affinity specific binding. This finding clearly distinguished the interaction from nonspecific associations of **10c** with neutral or zwitterionic lipid species, thus providing a clear mechanistic rationale for the 1**0c**’s wide therapeutic window and low toxicity to mammalian cells.

#### 3.8.2 Effect on bacterial surface potential

**Figure 7:**
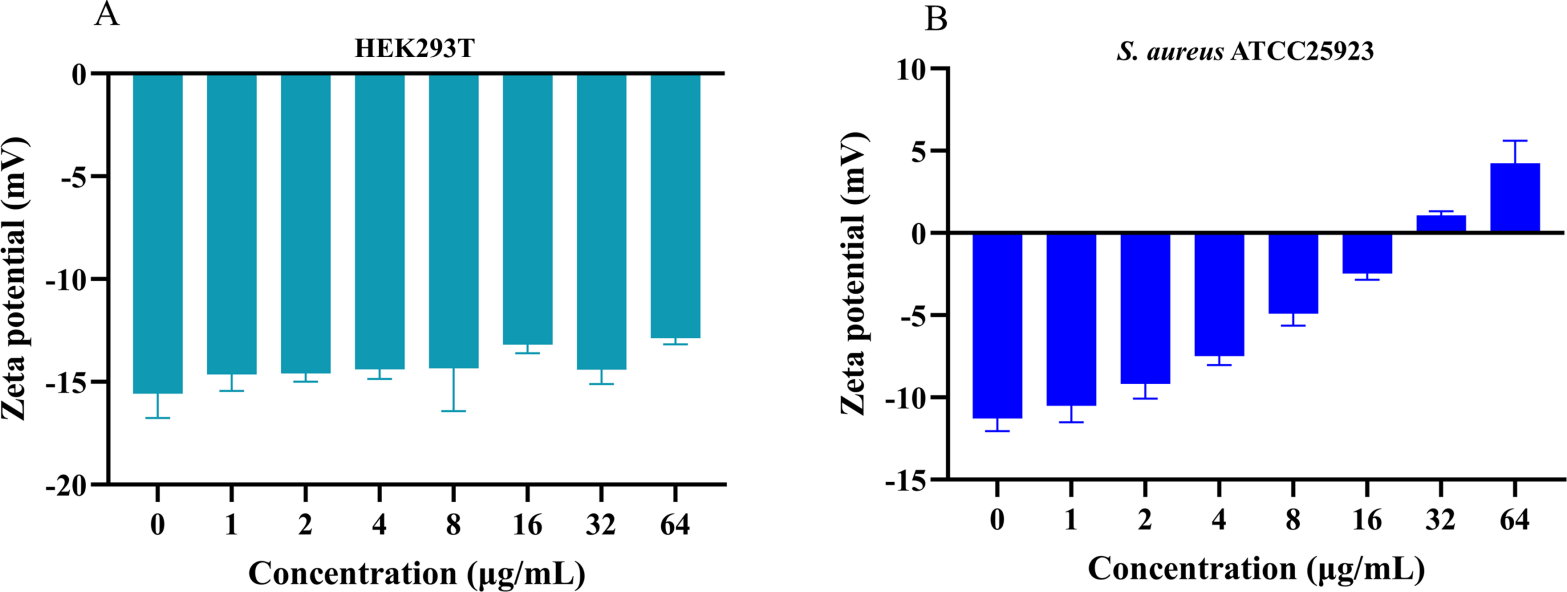
Alterations in surface potential induced by compound **10c** across bacterial and mammalian cell types. Zeta potential values were recorded for **(A)** HEK293T cells and **(B)** *S. aureus* ATCC25923 after treatment with increasing concentrations of **10c**. All data are expressed as the mean ± standard deviation of three independent measurements (*n* = 3).

Zeta potential is a key parameter characterizing the surface electrical properties of suspended cells, reflecting the potential distribution of the interfacial electrical double layer and serving as an effective indicator for monitoring changes in cell membrane stability and permeability abnormalities. To deeply investigate the differential mechanism of compound **10c** on bacterial versus mammalian cell membranes, this study systematically characterized the concentration-dependent dynamic changes in surface potential induced by **10c** in *S. aureus* ATCC25923 as well as in HEK293T mammalian cells. As shown in Figure 7, the experimental data revealed significant selectivity. In the Gram-positive bacterial system, increasing concentrations of **10c** led to a concentration-dependent positive shift in the surface potential of *S. aureus* (Figure 7B), indicating progressive structural perturbation and charge redistribution of the bacterial cell membrane. In stark contrast, the mammalian HEK293T cells exhibited a fundamentally different response pattern, maintaining a stable baseline surface potential regardless of **10c** concentration (Figure 7A). This differential action pattern demonstrated that **10c** specifically targeted the membrane surface charge characteristics of Gram-positive bacteria, inducing membrane destabilization through the reconstitution of bacterial membrane potential, while exerting no significant effect on the intrinsic electrical properties of mammalian cell membranes. This selective regulatory mechanism not only revealed the antibacterial specificity of **10c** from an electrophysical perspective but also provided a key theoretical basis for its excellent bacterial selectivity and favorable biocompatibility, further supporting its potential as a novel antibacterial drug candidate.

#### 3.8.3 Fluorescence microscopy

**Figure 8:**
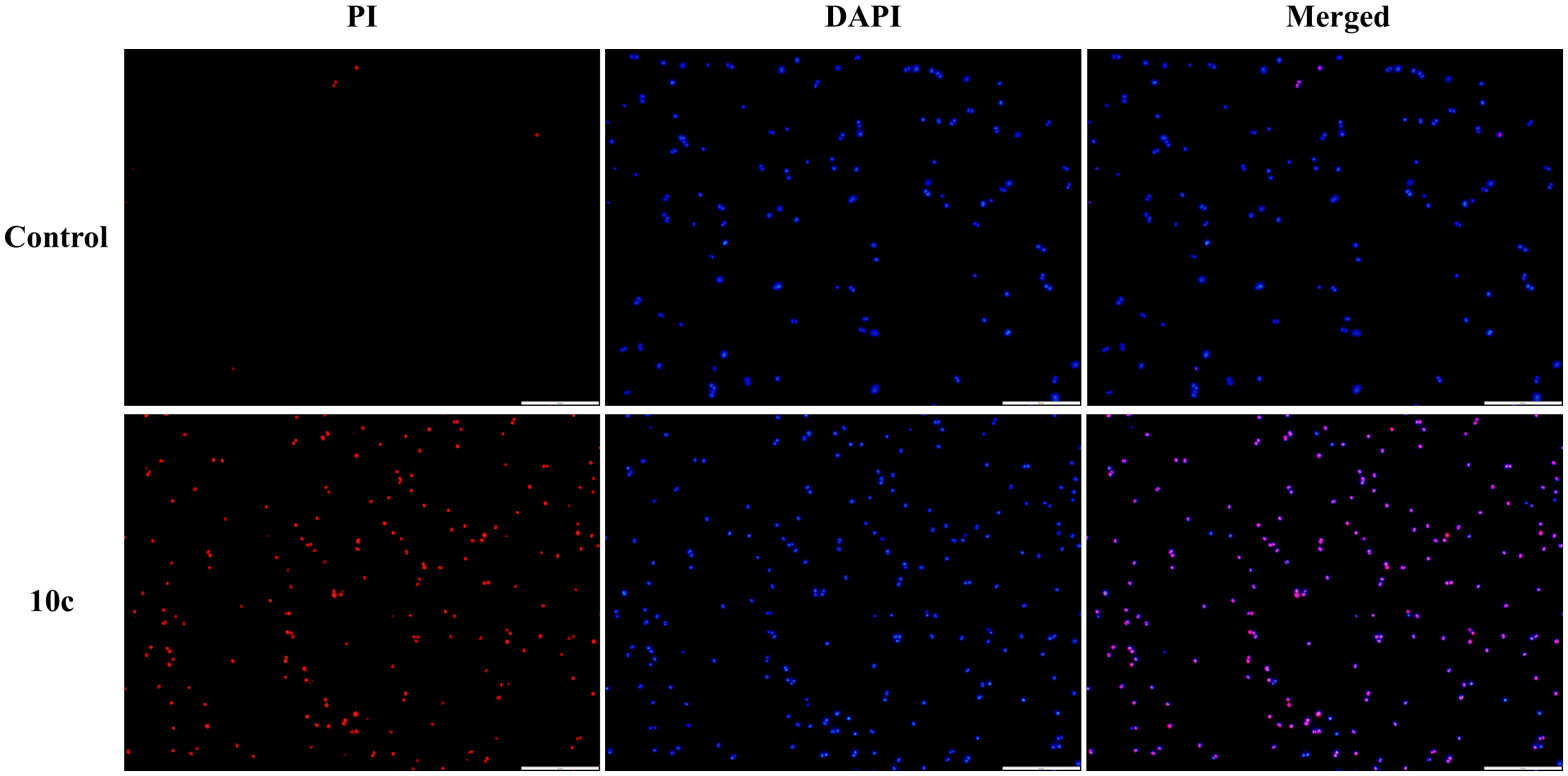
Visualization of membrane integrity in *S. aureus* ATCC25923 using dual-fluorescence labeling with DAPI (blue) and PI (red), scale bar = 20 μm.

To visually elucidate the effect of **10c** on bacterial membrane integrity, this study employed dual fluorescent probe staining using 4′,6-diamidino-2-phenylindole (DAPI) and propidium iodide (PI). DAPI, a membrane-permeable nucleic acid dye, can penetrate intact cell membranes and bind to nucleic acids to emit blue fluorescence, making it suitable for whole-cell staining and localization. In contrast, PI can only enter bacterial cells with compromised membrane integrity and intercalates into nucleic acids to emit red fluorescence, thereby serving as a specific indicator of membrane damage (Kong et al., 2023; Berney et al., 2007). As shown in Figure 8, untreated control cells displayed uniform and stable blue fluorescence, confirming that their cell membrane structure remained intact. Following treatment with compound **10c**, a significant fluorescence signal translocation was observed in the cell population: a large number of cells simultaneously exhibited both intense red fluorescence and co-localized blue signals, indicating that **10c** effectively breached the bacterial cell membrane barrier, allowing PI to penetrate the cells and specifically bind to nucleic acids. These results provided direct evidence that **10c** exerted its antibacterial activity through a membrane-disruptive mechanism, confirming that its mode of action is highly consistent with the paradigm of classical membrane-targeting antibacterial agents.

#### 3.8.4 Cytoplasmic membrane depolarization

**Figure 9:**
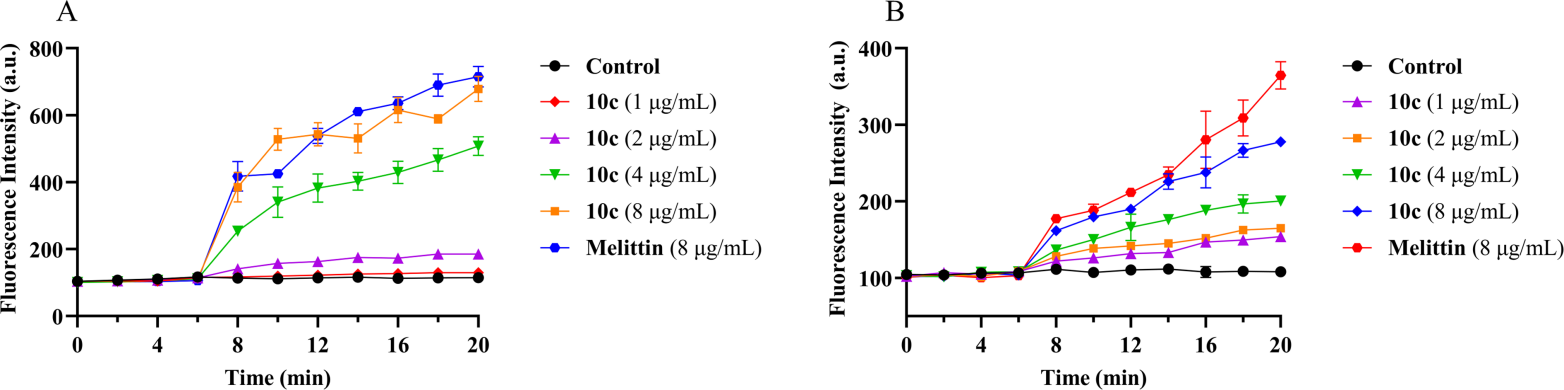
Studies on the mechanism by which **10c** targets the bacterial membrane in *S. aureus*. **(A)** Dissipation of the transmembrane electrical potential was assessed via the DiSC_3_(5) fluorescence assay, wherein depolarization manifests as an increase in probe fluorescence intensity. **(B)** Membrane permeability was evaluated by monitoring the cellular uptake of PI, a membrane-impermeant nucleic acid dye that gains intracellular entry only upon membrane compromise. Parallel analyses of untreated cells (negative control) and melittin-treated cells (positive control) were performed to establish baseline and maximal response thresholds, respectively.

To better understand how antibacterial activity correlates with disruption of the membrane potential, the probe DiSC_3_(5) was used for real-time monitoring. This probe is sensitive to changes in transmembrane potential: it accumulates within polarized membranes, where its fluorescence is quenched. When membrane potential dissipates (depolarization occurs), the probe is expelled into the external medium, leading to a substantial increase in fluorescence signal (Cheng et al., 2023). As illustrated in Figure 9A, the concentration-dependent membrane potential-disrupting effect of **10c** against *S. aureus* was captured by real-time fluorescence recordings. Treatment with **10c** at low dose (1 and 2 μg/mL) produced a fluorescence trace nearly identical to that of the untreated group, indicating no significant change in membrane potential. However, at higher concentrations (4–8 μg/mL), a steep fluorescence surge occurred, with peak intensities rising by 5.1- to 6.8-fold relative to the baseline. This effect was similar to that seen with the reference compound, melittin. These data demonstrated that compound **10c** could effectively trigger rapid depolarization of the bacterial membrane potential, revealing that membrane potential collapse is a core event in its bactericidal mechanism of action.

#### 3.8.5 Cell membrane permeabilization

To further investigate whether compound **10c** compromised bacterial membranes, a PI uptake assay was conducted. As demonstrated in Figure 9B, exposure of *S. aureus* to **10c** led to a rapid, concentration-dependent increase in PI fluorescence. When cells were treated with 4 μg/mL of **10c**, the fluorescence signal rose approximately 2.8-fold relative to the control (no treatment) over a 20-min period. Upon increasing the concentration to 8 μg/mL, an even greater fluorescence enhancement was observed, reaching levels similar to those induced by melittin. In contrast, the untreated control group showed no significant change in fluorescence, remaining at baseline levels throughout. These outcomes unequivocally demonstrated that the bacterial cytoplasmic membrane was successfully rendered permeable by treatment with **10c**.

#### 3.8.6 Secondary outcomes associated with membrane disruption

**Figure 10:**
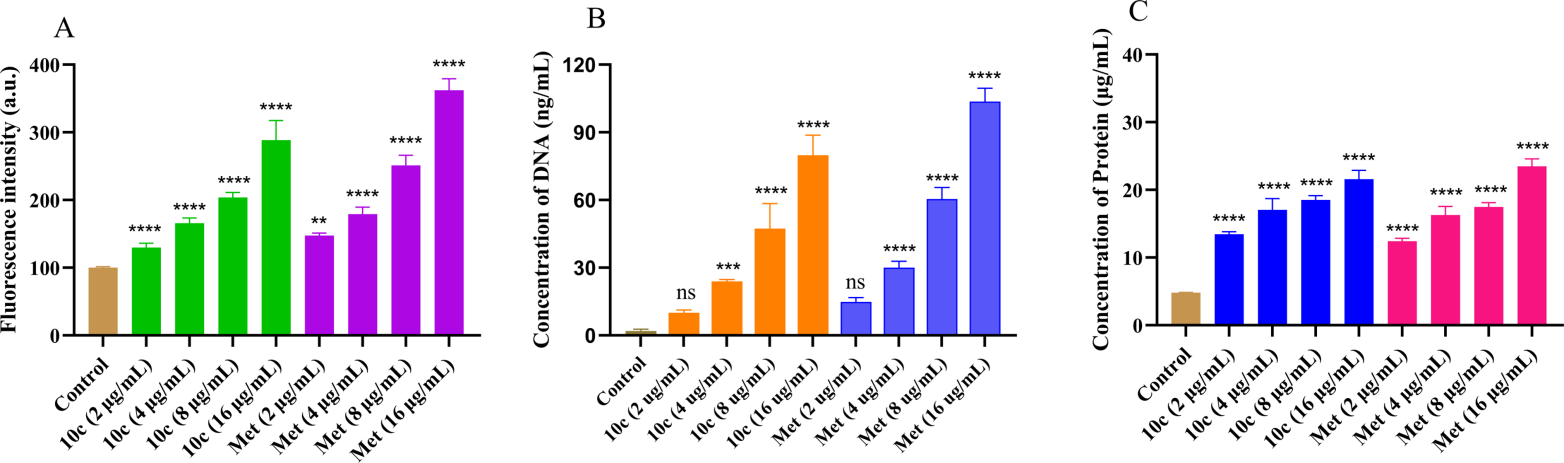
Mechanistic investigation of the antibacterial action of compound **10c** against *S. aureus* ATCC 25923, with melittin used as a reference drug. **(A)** Assessment of intracellular ROS generation. **(B)** Measurement of extracellular DNA released into the culture supernatant. **(C)** Measurement of protein leakage as an indicator of membrane permeabilization. Values are shown as means ± SD calculated from three biologically independent replicates. To determine statistical significance, a one-way ANOVA test was applied (ns, not significant; **p < 0.01, ***p < 0.001; ****p < 0.0001 versus the respective control groups).

The bacterial cell membrane serves as a critical barrier for maintaining intracellular homeostasis, and its structural disruption often triggers a lethal cascade of downstream events (Weng et al., 2024). This study investigated the downstream biological effects induced by compound **10c**, with a focus on the generation of ROS and leakage of intracellular macromolecules. The intracellular oxidative stress response of *S. aureus* ATCC25923 was first assessed using the DCFH-DA fluorescent probe. As demonstrated in Figure 10A, compound **10c** dramatically altered the level of intracellular ROS in a dose-dependent fashion. At 16 μg/mL, the ROS level increased to 2.9-fold higher than that of the control, an effect comparable to that of reference peptide melittin (3.6-fold). This intense oxidative burst, acting together with membrane disruption, likely promoted rapid and irreversible bacterial cell death. In parallel, the release of cytoplasmic components was quantitatively measured to provide direct physical evidence of membrane barrier breakdown. The data (Figures 10B,C) demonstrated that increasing concentrations of **10c** led to a clear dose-dependent rise in extracellular DNA and protein levels. At 8 μg/mL, the amounts of DNA and protein in the external medium were 19.5-fold and 3.8-fold higher, respectively, than those observed in the PBS. Increasing the dose to 16 μg/mL further intensified the membrane-disruptive effect, resulting in DNA and protein leakage that reached 33.2-fold and 4.5-fold relative to the control. Taken together, compound **10c** operated through a dual-action bactericidal mechanism, the combination of physical membrane disruption and chemical oxidative stress accounted for its rapid and strong antibacterial efficacy.

### 3.9 *In vivo* antibacterial activity

**Figure 11:**
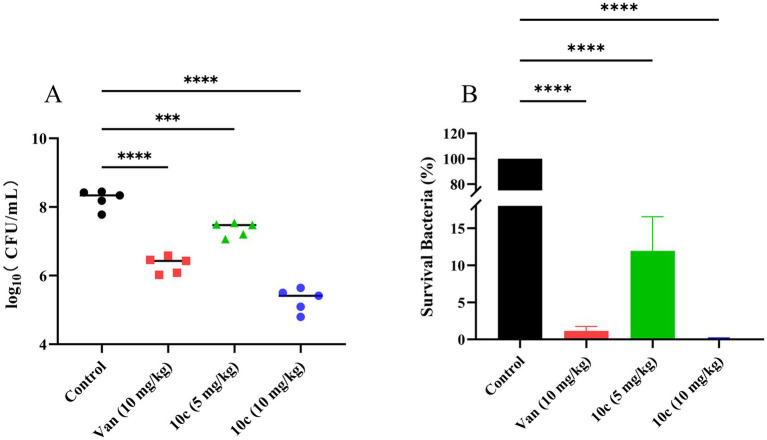
Evaluation of compound **10c** against *S. aureus* skin infection *in vivo*. **(A)** Quantification of bacterial burden in infected tissue homogenates from mice treated with PBS, vancomycin (Van), or **10c**. **(B)** Reduction in bacterial viability expressed as percentage survival. Data are shown as mean ± SD (*n* = 5 biologically independent samples). Statistical differences were determined by one-way ANOVA test (***p < 0.001, ****p < 0.0001).

Based on the encouraging *in vitro* findings, the *in vivo* efficacy of compound **10c** was evaluated using a murine model of skin abscess induced by *S. aureus* ATCC25923. The infection model was first established by subcutaneous injection of bacterial suspension (6 × 10^8^ CFU/mL, 100 μL) into the back of the SPF male BALB/c mice (6–8 weeks old, weighing 18–22 g). After 2 h of the injection, the animals were treated with either a low dose (5 mg/kg, 100 μL) or a high dose (10 mg/kg, 100 μL) of **10c** via local subcutaneous injection around the abscess sites. PBS was administered to the negative control group, while vancomycin (10 mg/kg, 100 μL) served as the positive control. All injections were repeated every 12 h over a period of 2 days, and the progression of abscess appearance was further monitored over a 24 h period. After that, the mice were euthanized. Treatment outcomes were assessed by measuring bacterial counts in infected skin tissues and by examining histopathological changes through H&E staining. As shown in Figure 11, compound **10c** led to a significant, dose-dependent decrease in bacterial burden relative to the PBS control. At the low dose of 5 mg/kg, **10c** lowered the bacterial load in infected skin by 0.81 log units (corresponding to 88.1% killing). At the higher dose, it reduced the bacterial load by approximately 2.92 log_10_ units (99.9% killing) at the infection site, outperforming vancomycin, which at an equivalent dose achieved a 1.88 log10 reduction (98.2% killing).

**Figure 12:**
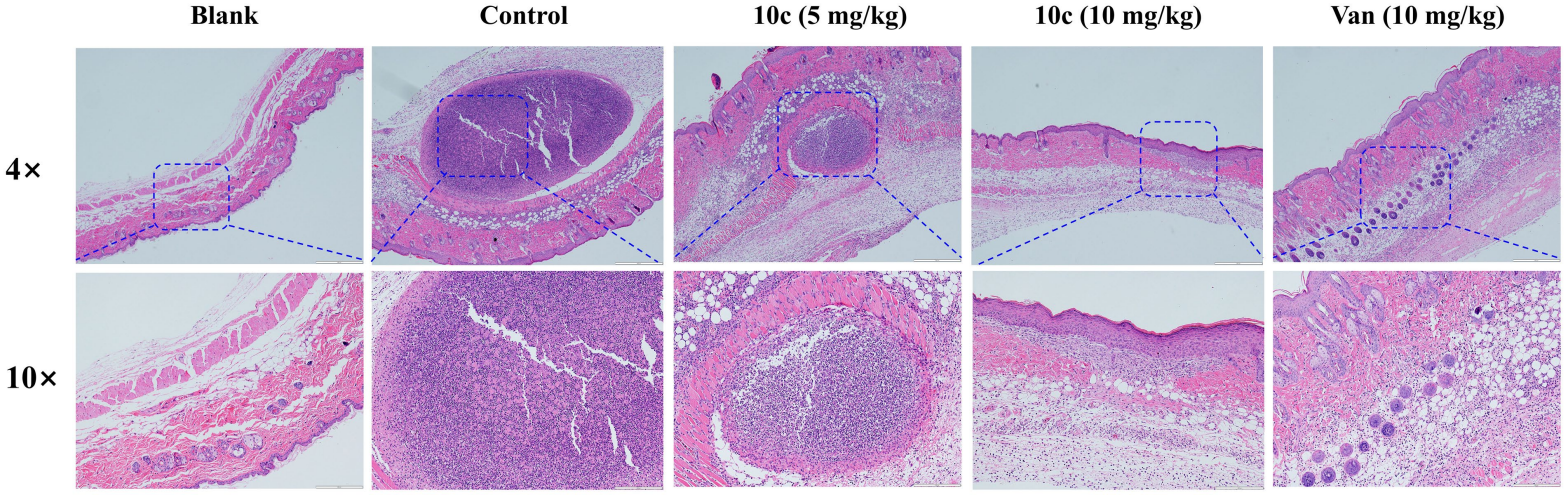
Histological changes in mouse skin tissue following infection, as evaluated by H&E staining. Representative sections are presented for the blank control, PBS-treated (vehicle control), vancomycin-treated (Van), and compound **10c**-treated groups. Scale bars represent 500 μm (4× magnification) and 200 μm (10× magnification).

To gain further insight into the *in vivo* activity of compound **10c**, histological examination of infected skin tissues was performed. In the control group, H&E-stained sections revealed extensive inflammatory cell infiltration, predominantly neutrophils, within the subcutaneous layer, indicative of a robust local immune response. In contrast, treatment with **10c** markedly alleviated this inflammation, with only mild and scattered neutrophil presence at the site of infection. Notably, at a dose of 10 mg/kg, compound **10c** reduced inflammatory cell infiltration in infected tissues to levels nearly equivalent to those seen in uninfected control animals (Figure 12). Collectively, these results demonstrated that compound **10c** exhibits potent *in vivo* antibacterial activity, effectively eliminating bacterial loads and ameliorating infection-associated tissue injury.

## 4 Conclusion

In summary, through rational design and systematic optimization of natural product-derived scaffolds, a series of cinnamic acid-guanidinium derivatives that effectively mimic the amphiphilic properties of cationic AMPs were synthesized. Following systematic bioactivity evaluation, Compound **10c** emerged as the most effective derivative, exhibiting strong and broad-spectrum antibacterial effects against Gram-positive pathogens, with MIC values ranging from 1 to 2 μg/mL. In-depth mechanistic studies confirmed that **10c** selectively disrupted the bacterial membrane through specific binding to PG within the membrane. This interaction triggered membrane depolarization, increased membrane permeability, and subsequently led to the generation of ROS and leakage of intracellular contents, ultimately resulting in bactericidal effects. This membrane-targeting mode of action endowed **10c** with several therapeutic advantages, including fast bactericidal action, a high barrier to resistance development, and effective anti-biofilm potency. Furthermore, **10c** showed an excellent safety profile, characterized by minimal hemolysis and cytotoxicity, along with favorable stability in plasma. Remarkably, **10c** demonstrated greater therapeutic efficacy than vancomycin in a mouse skin abscess model. Overall, these findings confirmed that membrane-targeting amphiphilic molecules represent a promising strategy to combat the antimicrobial resistance crisis and provided strong support for the further development of this class of drug candidates.

## Data Availability

The original contributions presented in the study are included in the article/Supplementary material, further inquiries can be directed to the corresponding authors.
