# Supplementary material for: Discovery of guanidinium salt incorporating cinnamic acid skeleton as new antibacterial agents against Gram-positive bacteria
Source: Front Microbiol. 2026 Jul 15;17:1906096. doi: 10.3389/fmicb.2026.1906096 (PMC13416634; doi:10.3389/fmicb.2026.1906096)
Supplement: Supplementary file 1 [file Table_1.docx]

Supplementary Material

**Discovery of** **Guanidinium Salt Incorporating** **Cinnamic Acid Skeleton As New Antibacterial Agents Against Gram-positive Bacteria**

**Fen Zhou ^1^, Ping Zhao ^1^, Mengqi Liu ^2^, Hongxin Li ^1,^ *, Yinhu Wang ^2,^ * and Liang Liang ^1,^ ***

|  |
| --- |

^1^ Department of Pharmacy, Liaocheng People′s Hospital, Shandong Province, Liaocheng 252000, China

^2^ School of Pharmaceutical Sciences and Food Engineering, Liaocheng University, Liaocheng 252059, China

Correspondence: hongxinli1989@163.com (HL); wangyinhuabc@126.com (YW); LiangL1515@163.com (LL)

Supplementary data

**Index:**

^1^H NMR, ^13^C NMR Spectra and HRMS of target compounds 2-18


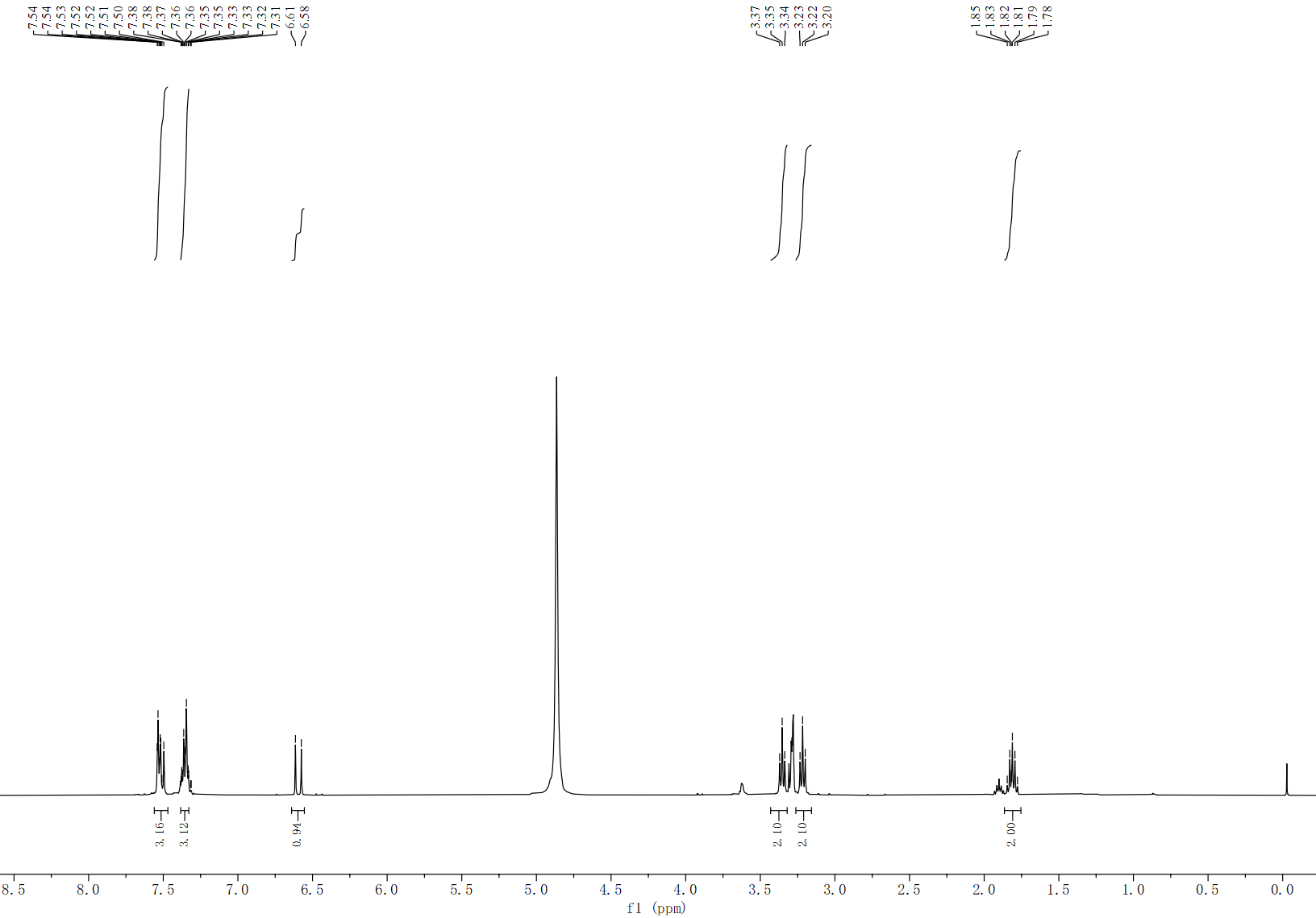

**^1^H NMR spectrum of 6a**


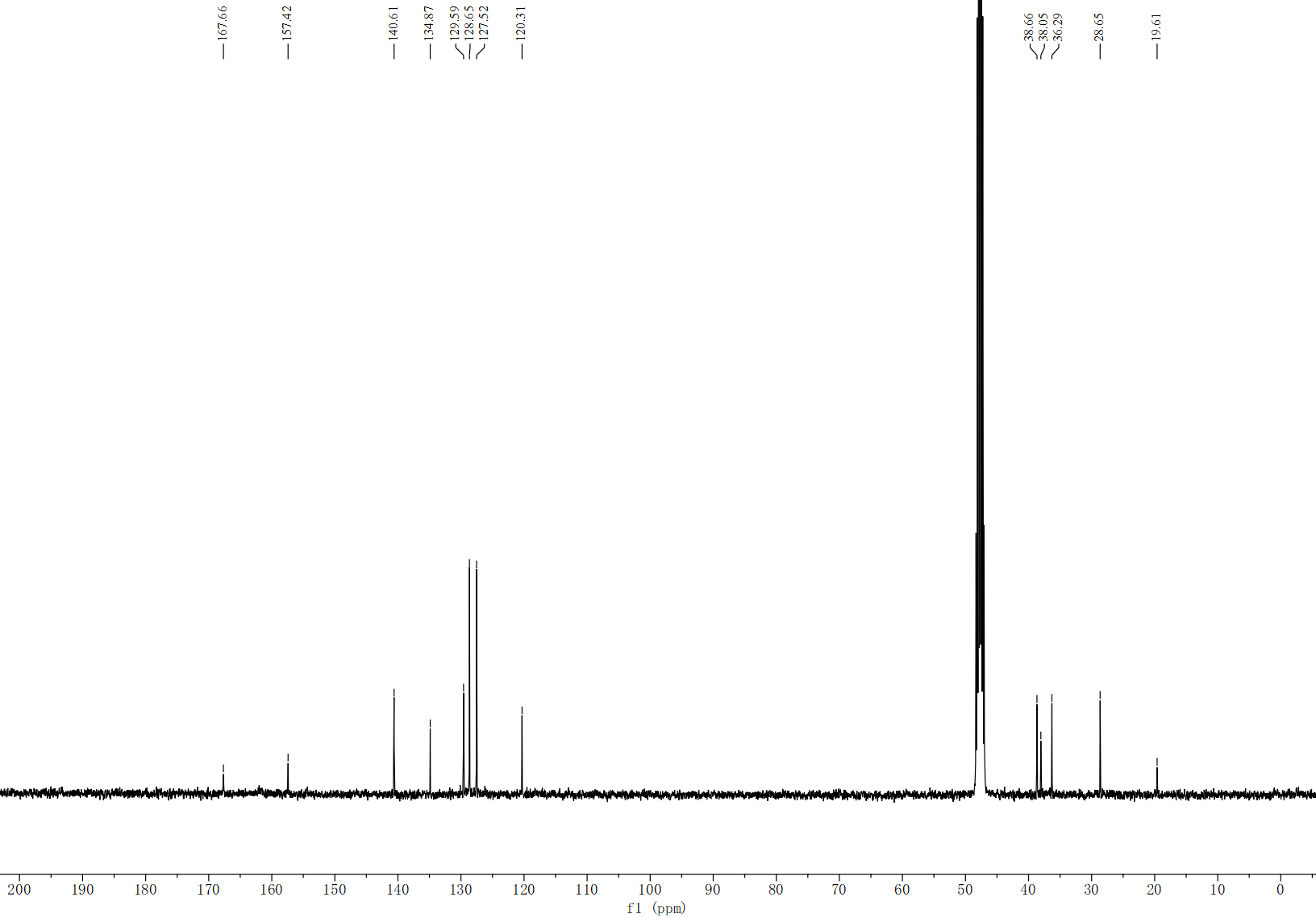


**^13^C NMR spectrum of 6a**


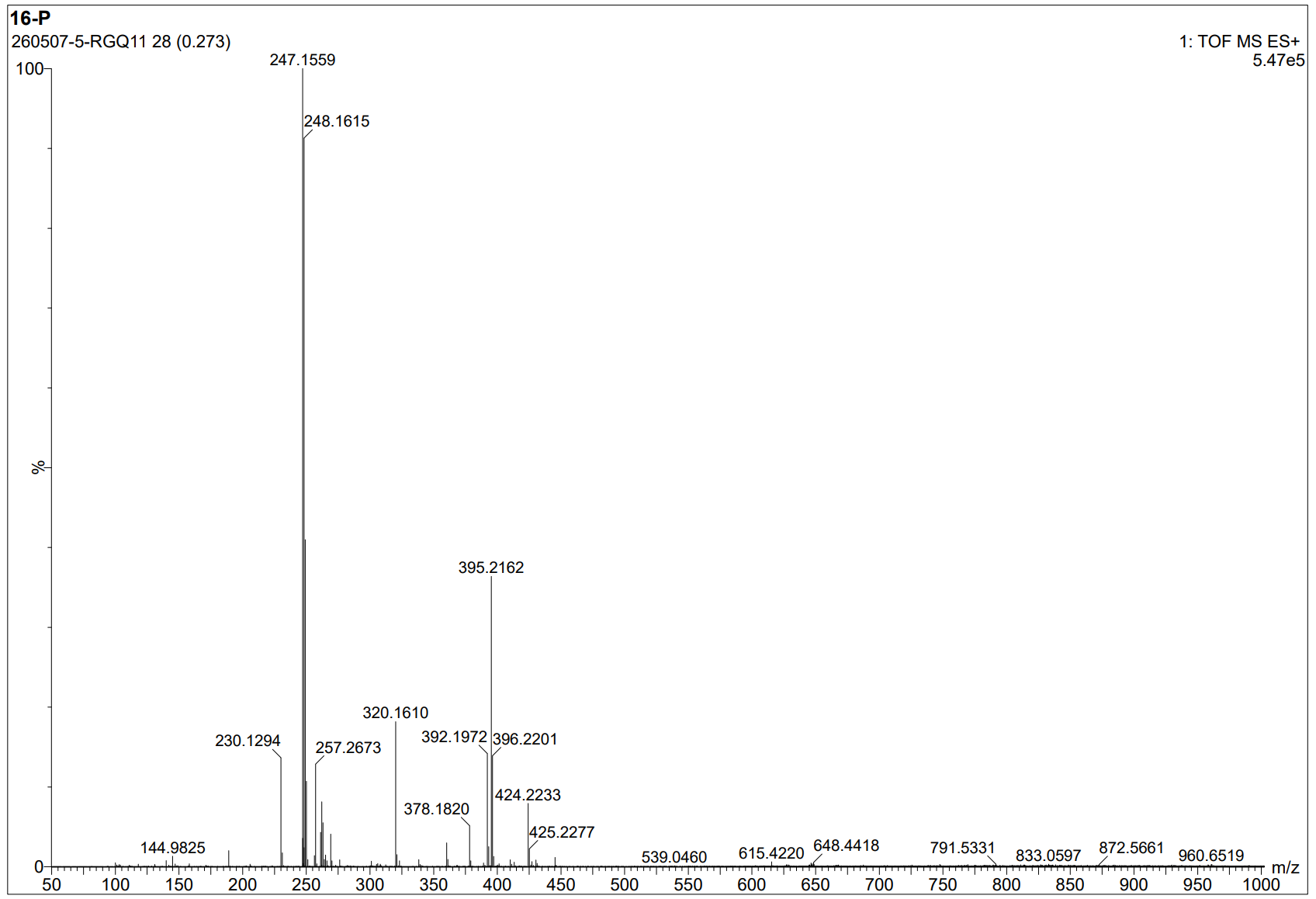


**HRMS spectrum of 6a**


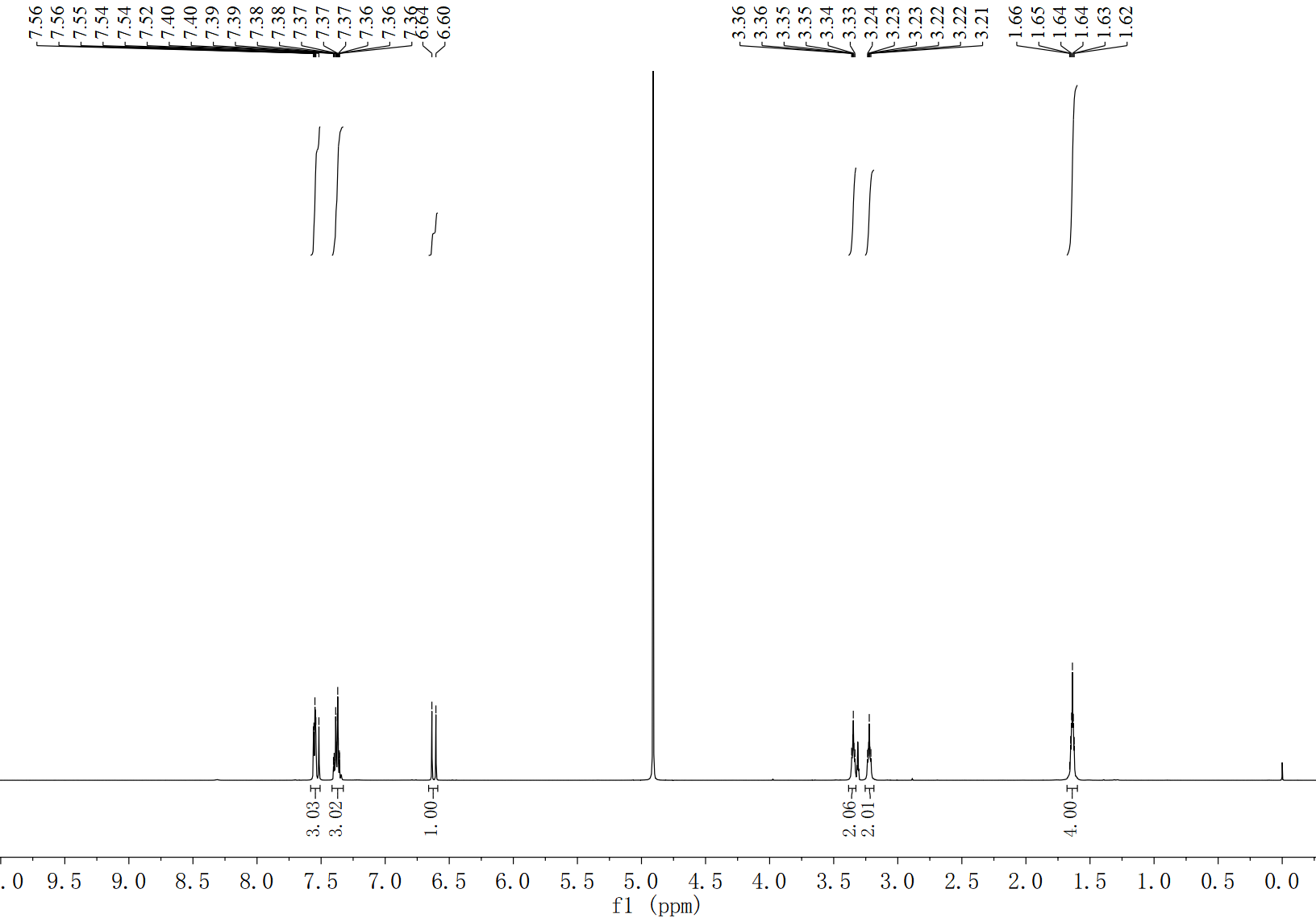

**^1^H NMR spectrum of 6b**


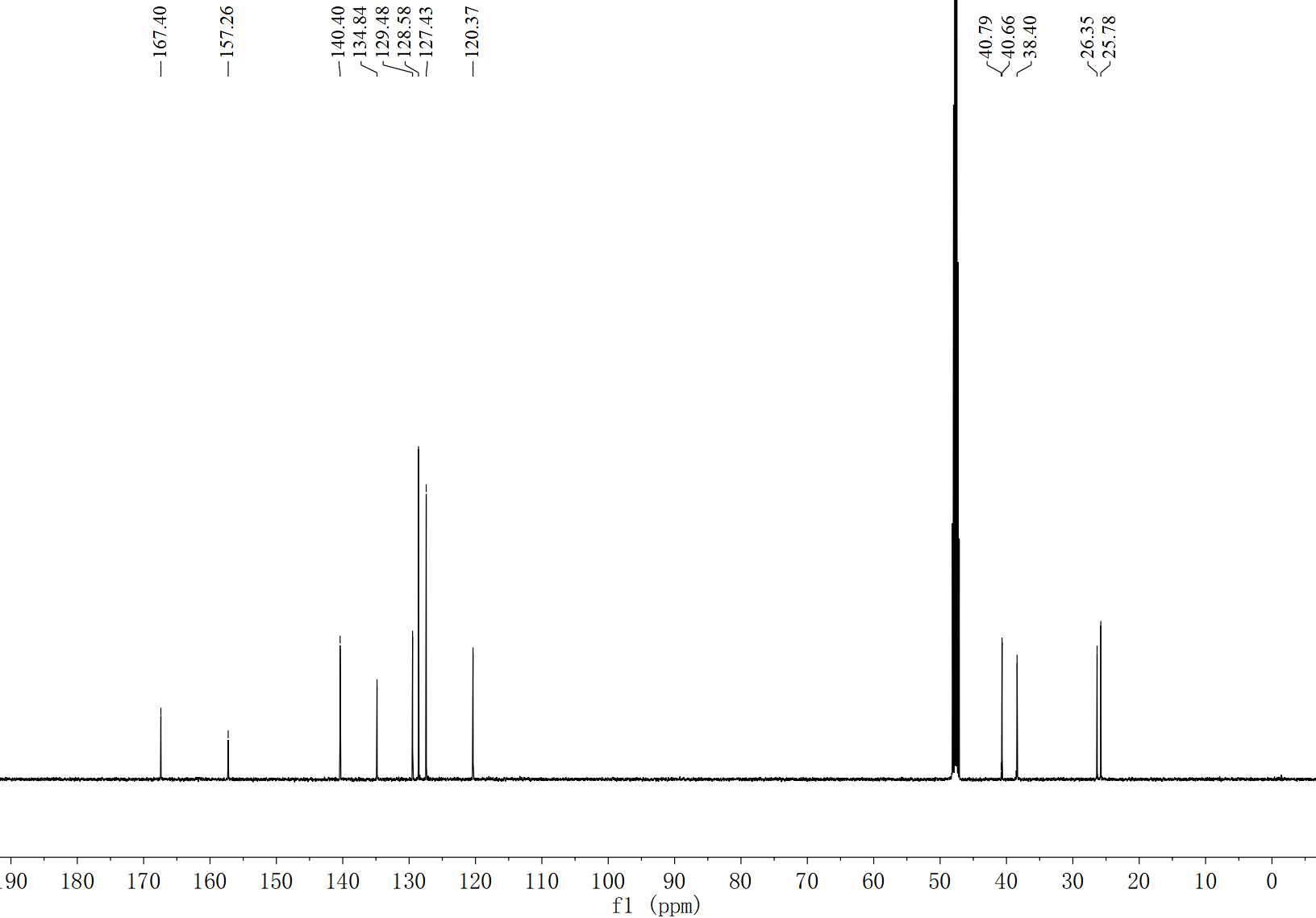


**^13^C NMR spectrum of 6b**


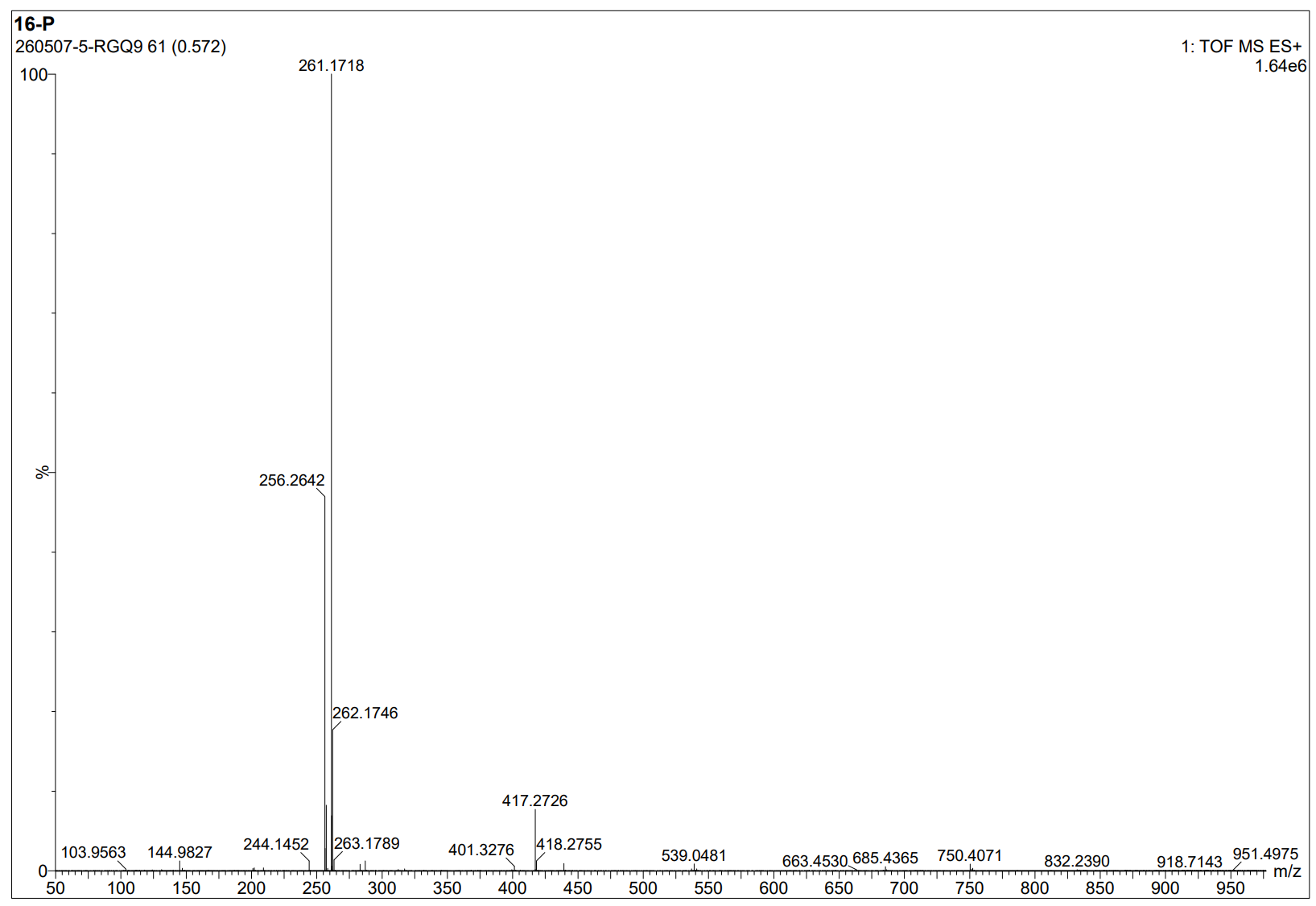


**HRMS spectrum of 6b**


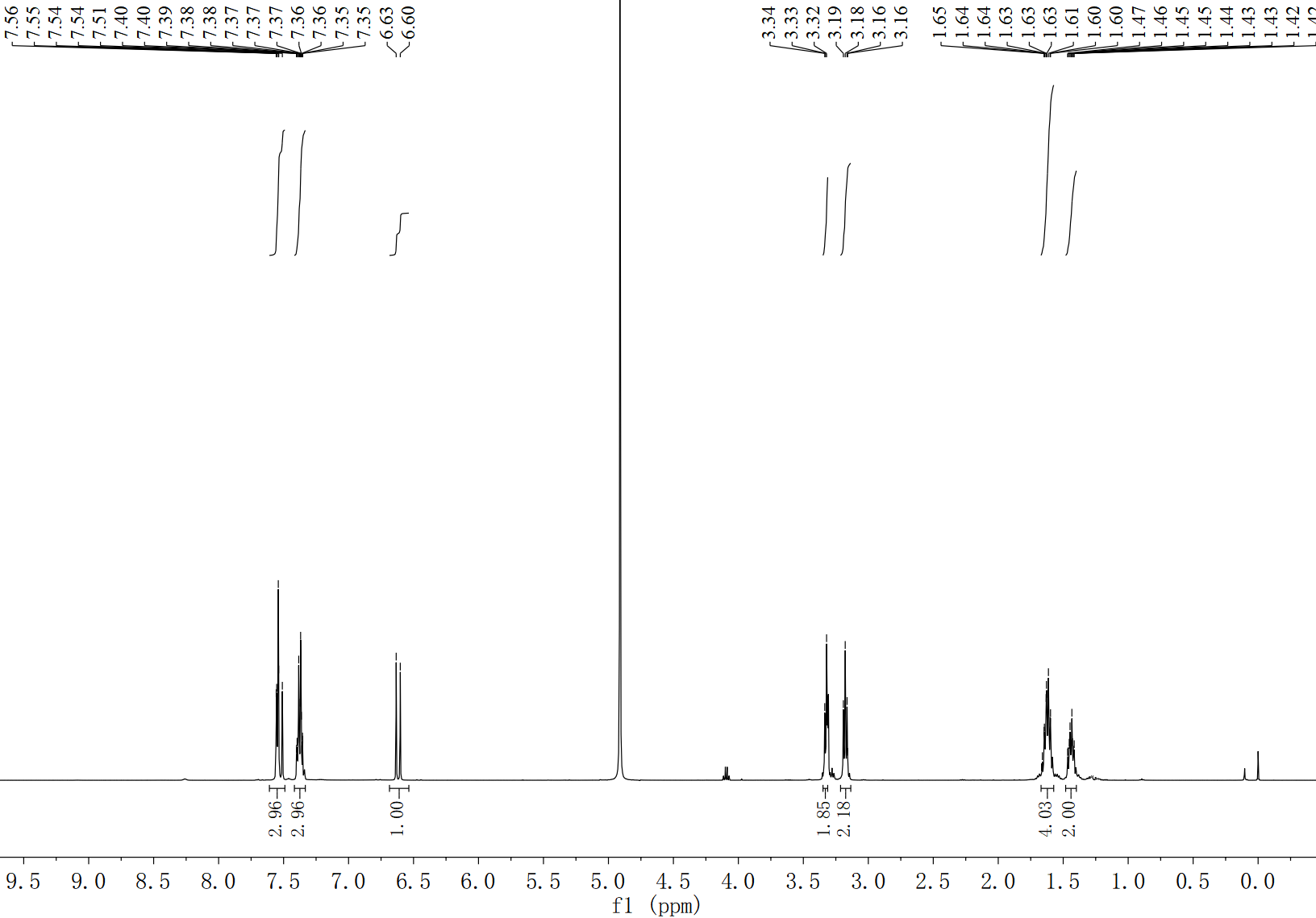

**^1^H NMR spectrum of 6c**


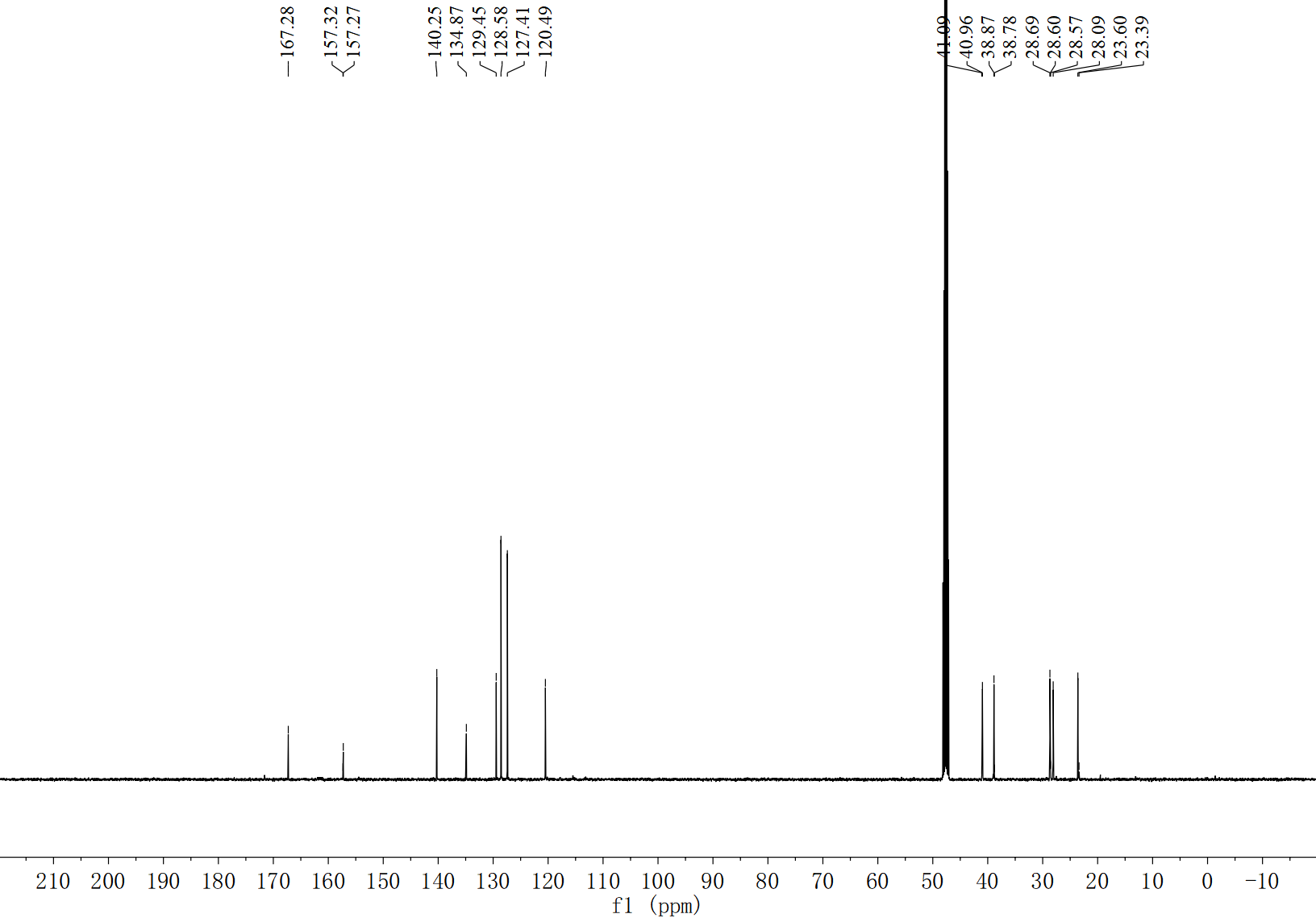


**^13^C NMR spectrum of 6c**


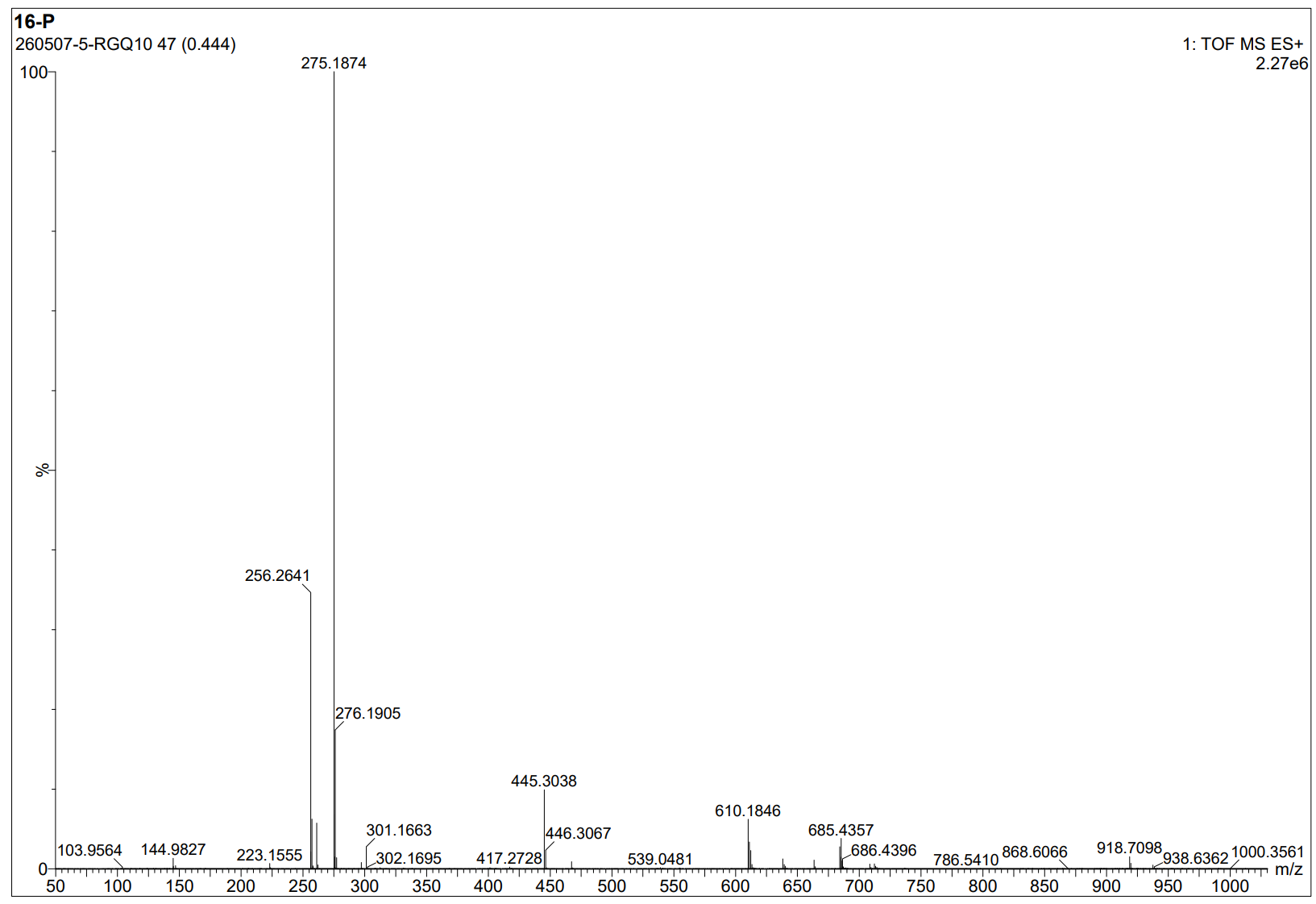


**HRMS spectrum of 6c**


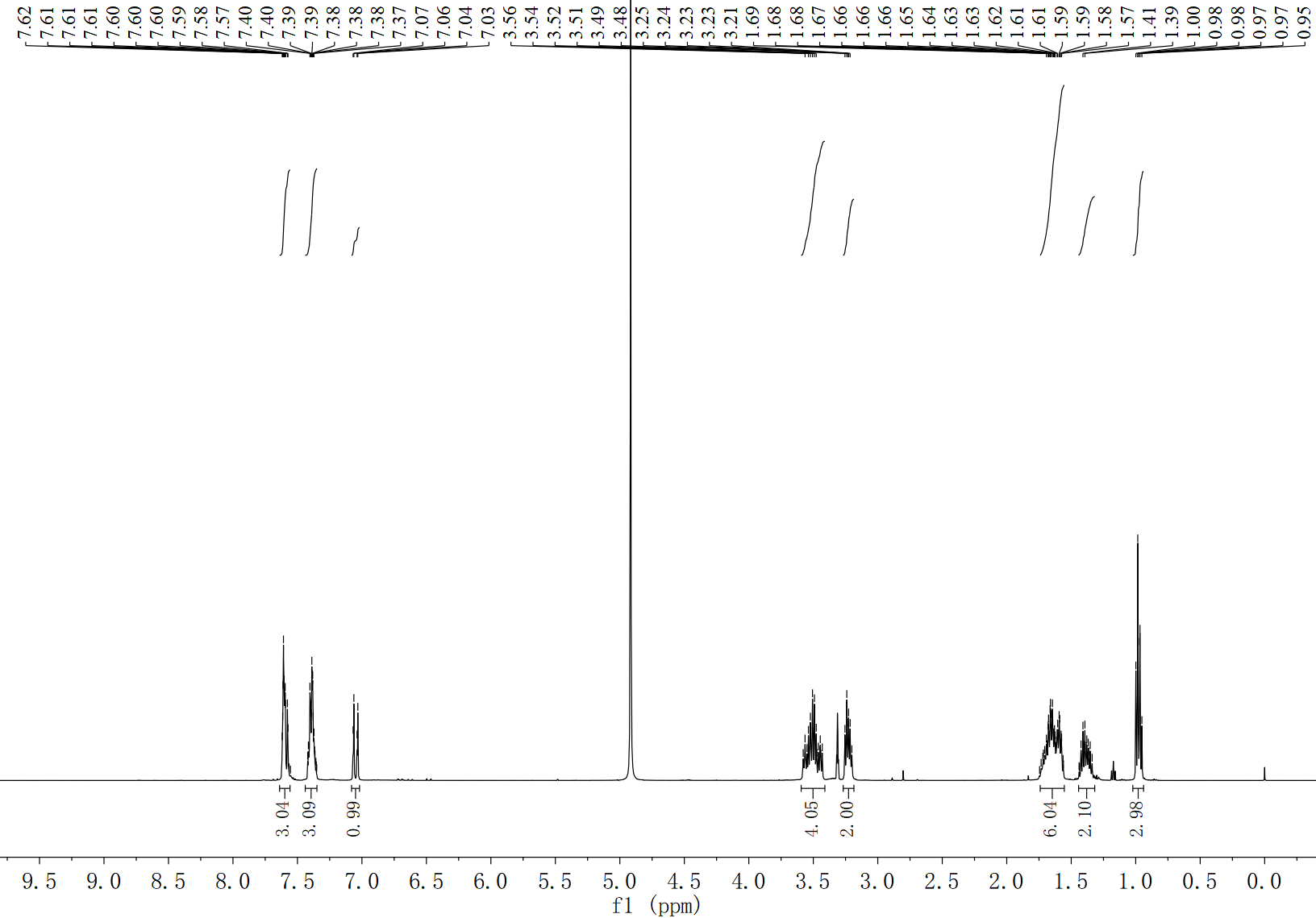

**^1^H NMR spectrum of 10a**


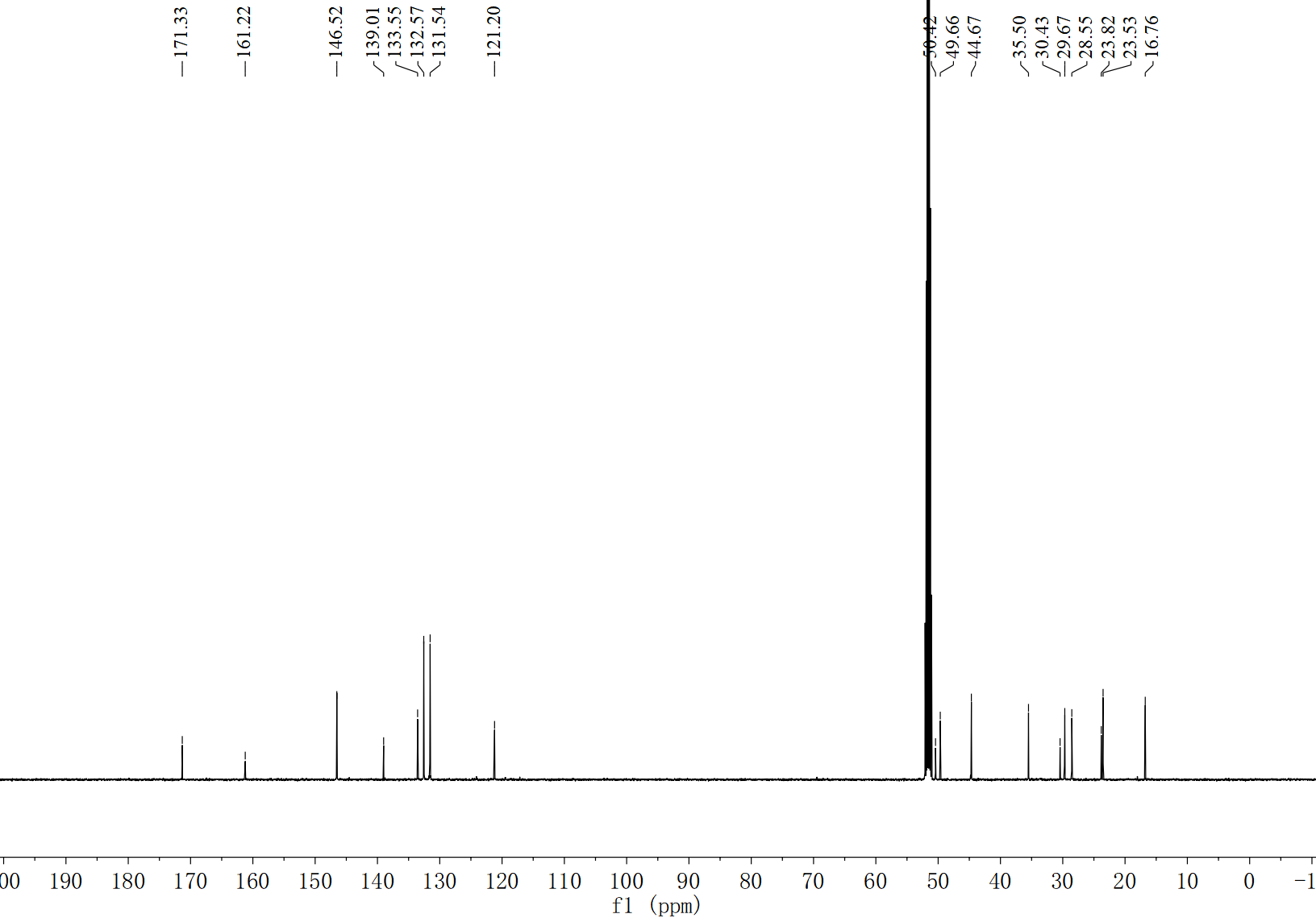


**^13^C NMR spectrum of 10a**


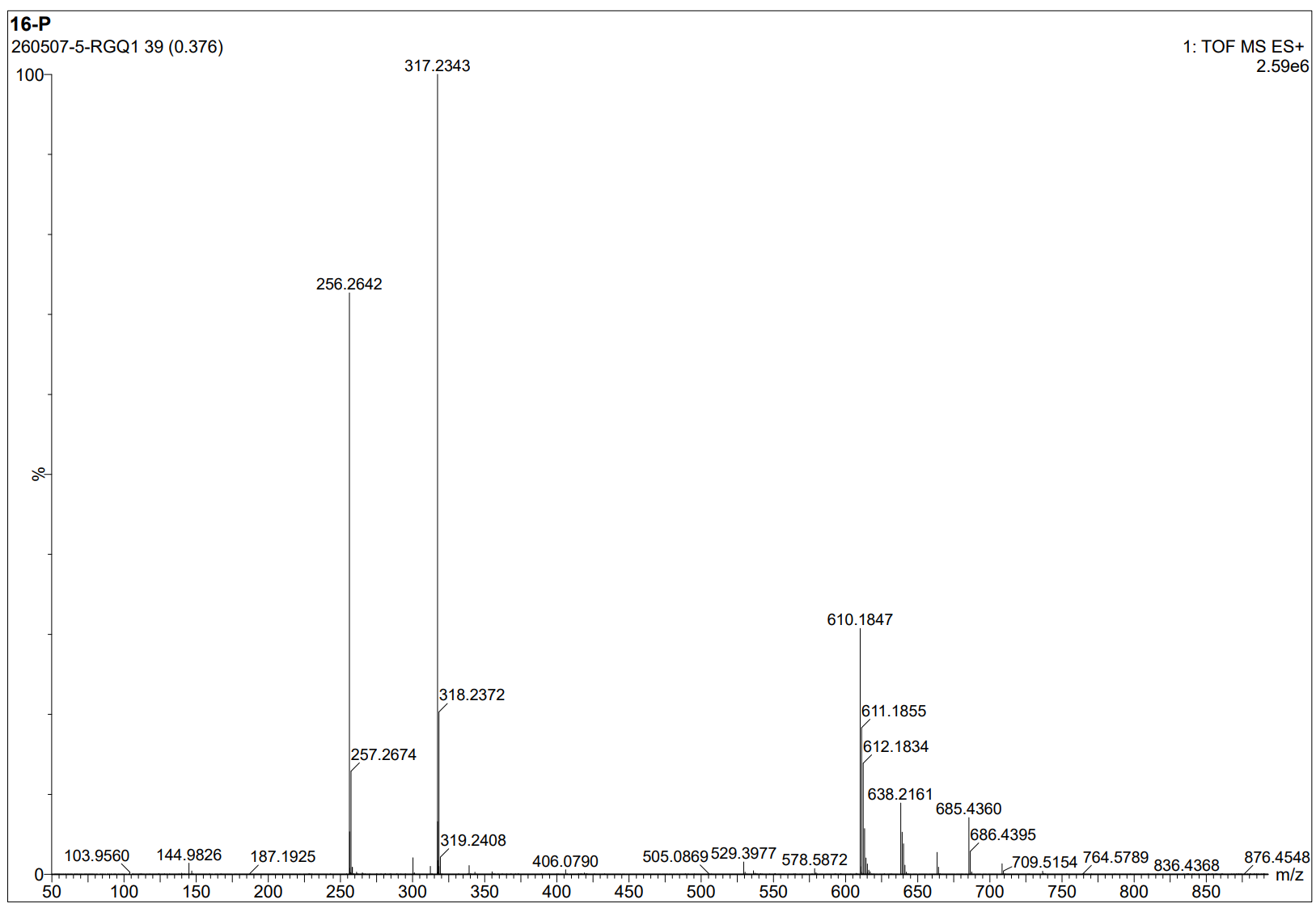


**HRMS spectrum of 10a**


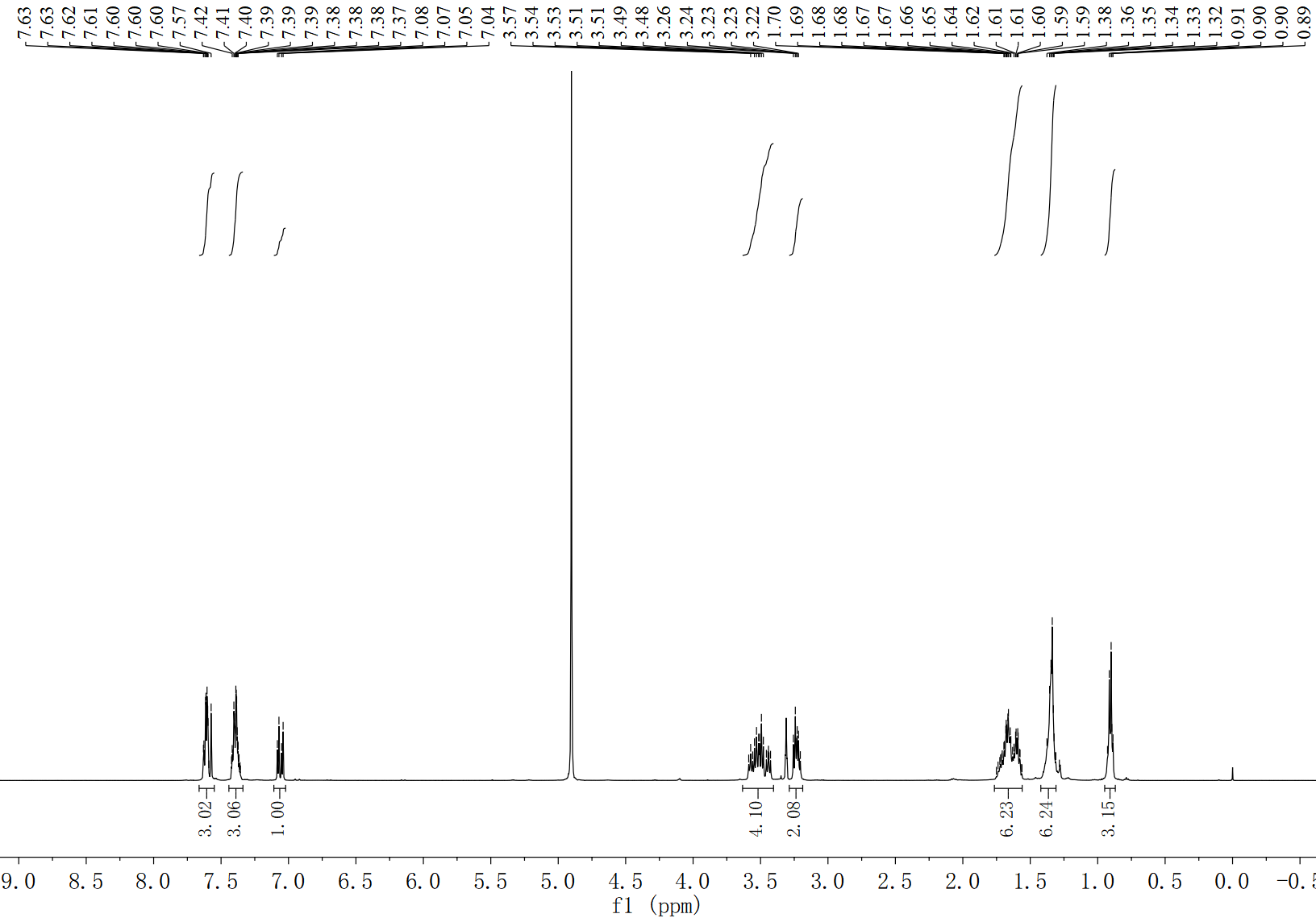

**^1^H NMR spectrum of 10b**


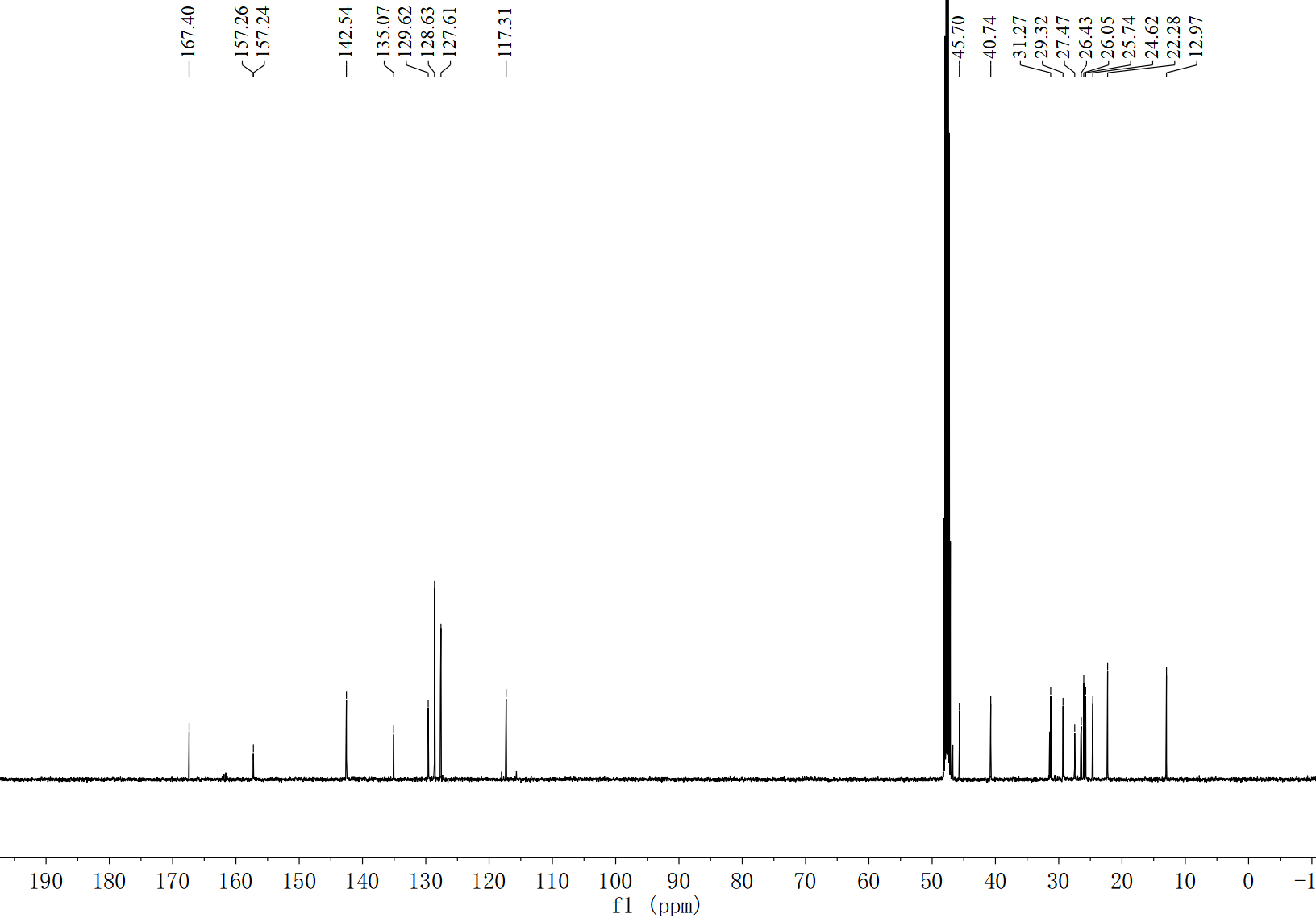


**^13^C NMR spectrum of 10b**


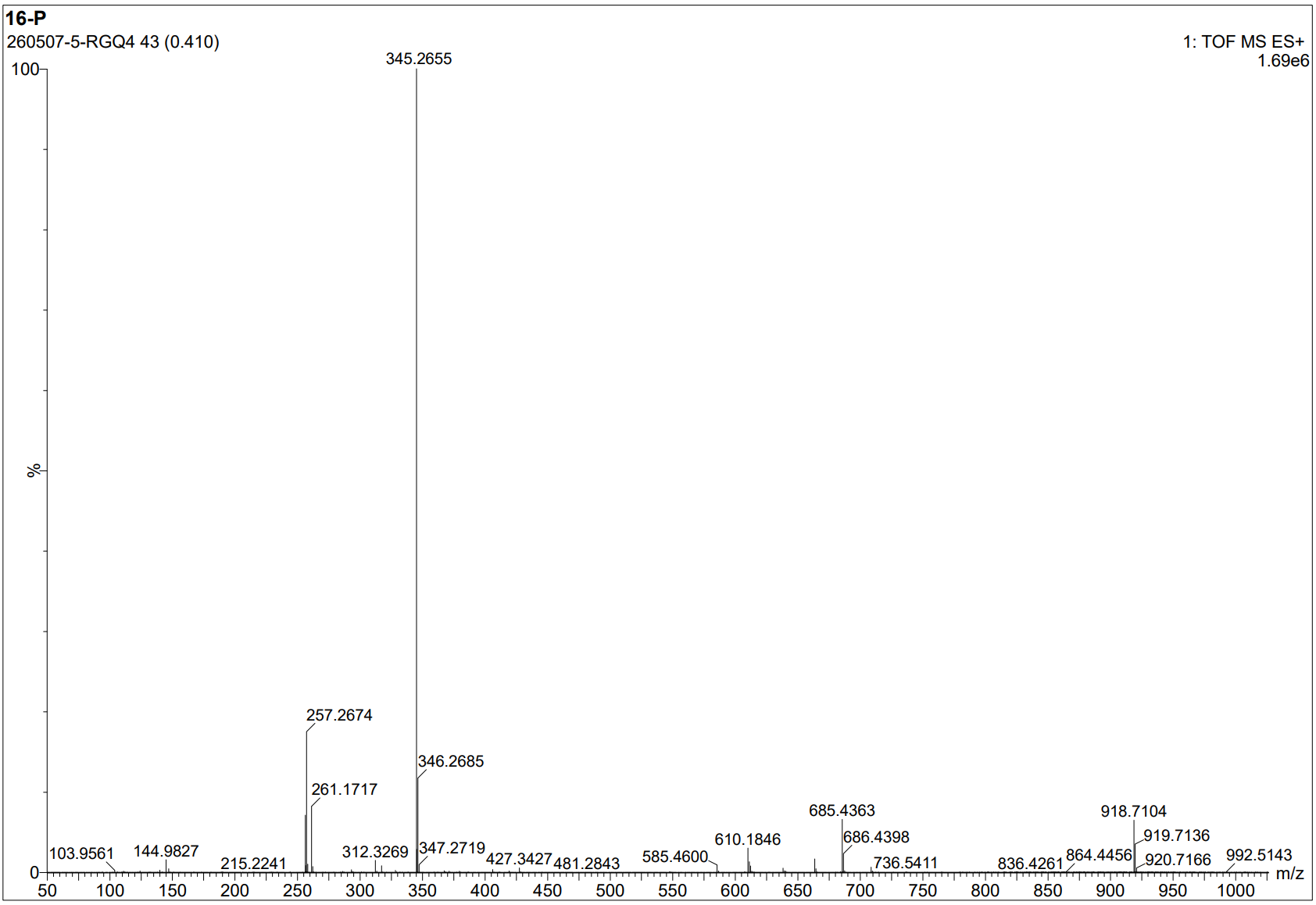


**HRMS spectrum of 10b**


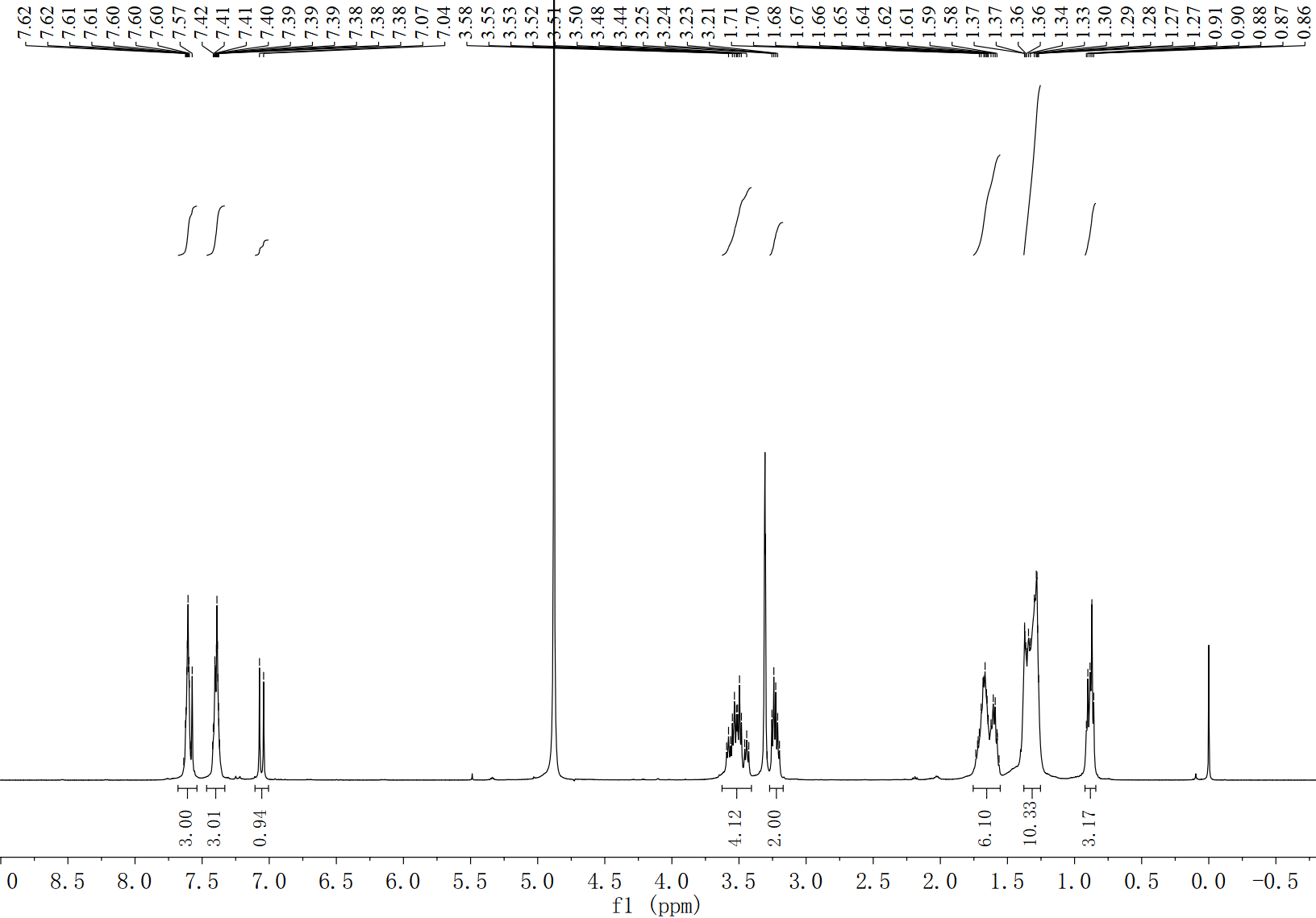

**^1^H NMR spectrum of 10c**


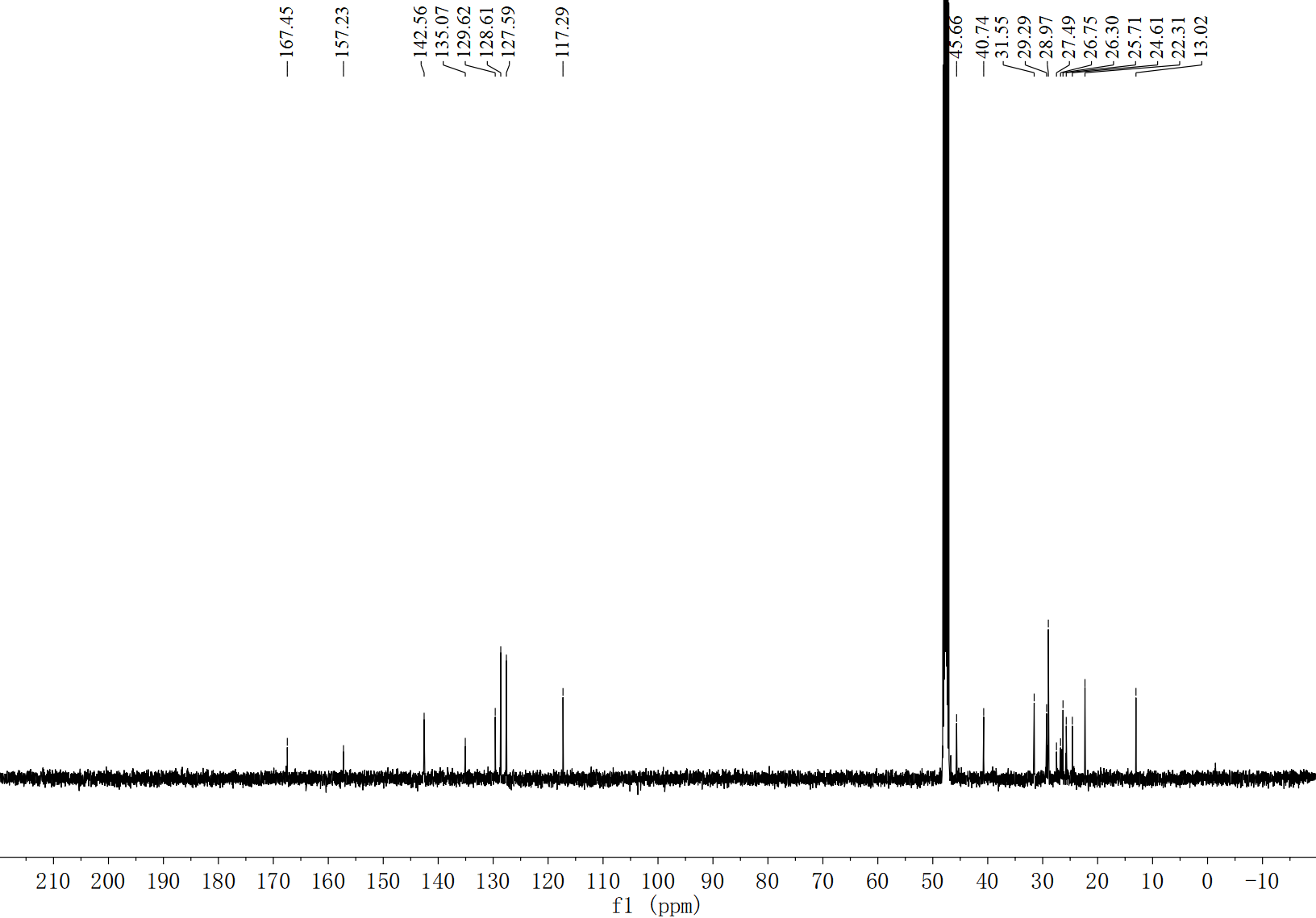


**^13^C NMR spectrum of 10c**


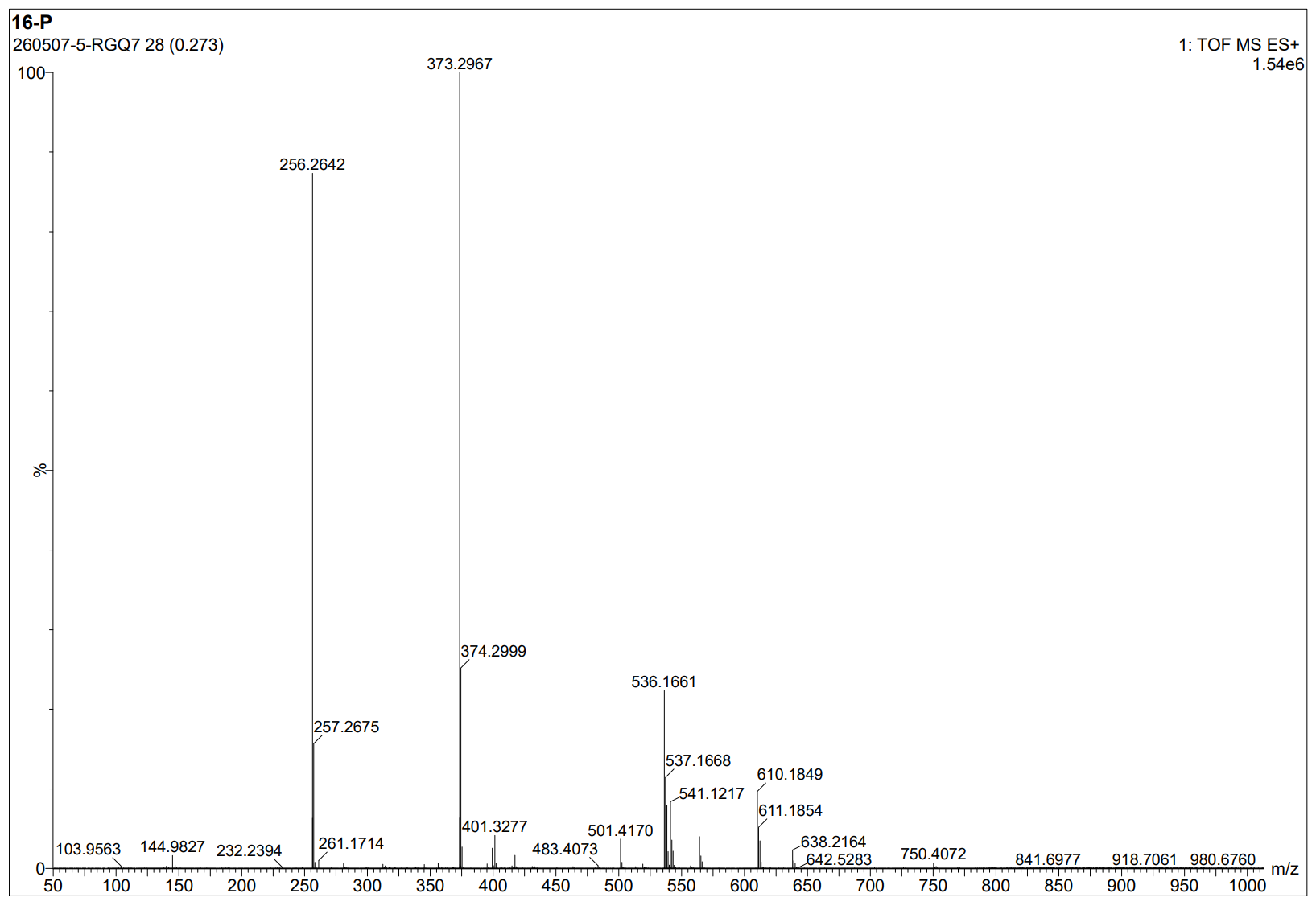


**HRMS spectrum of 10c**


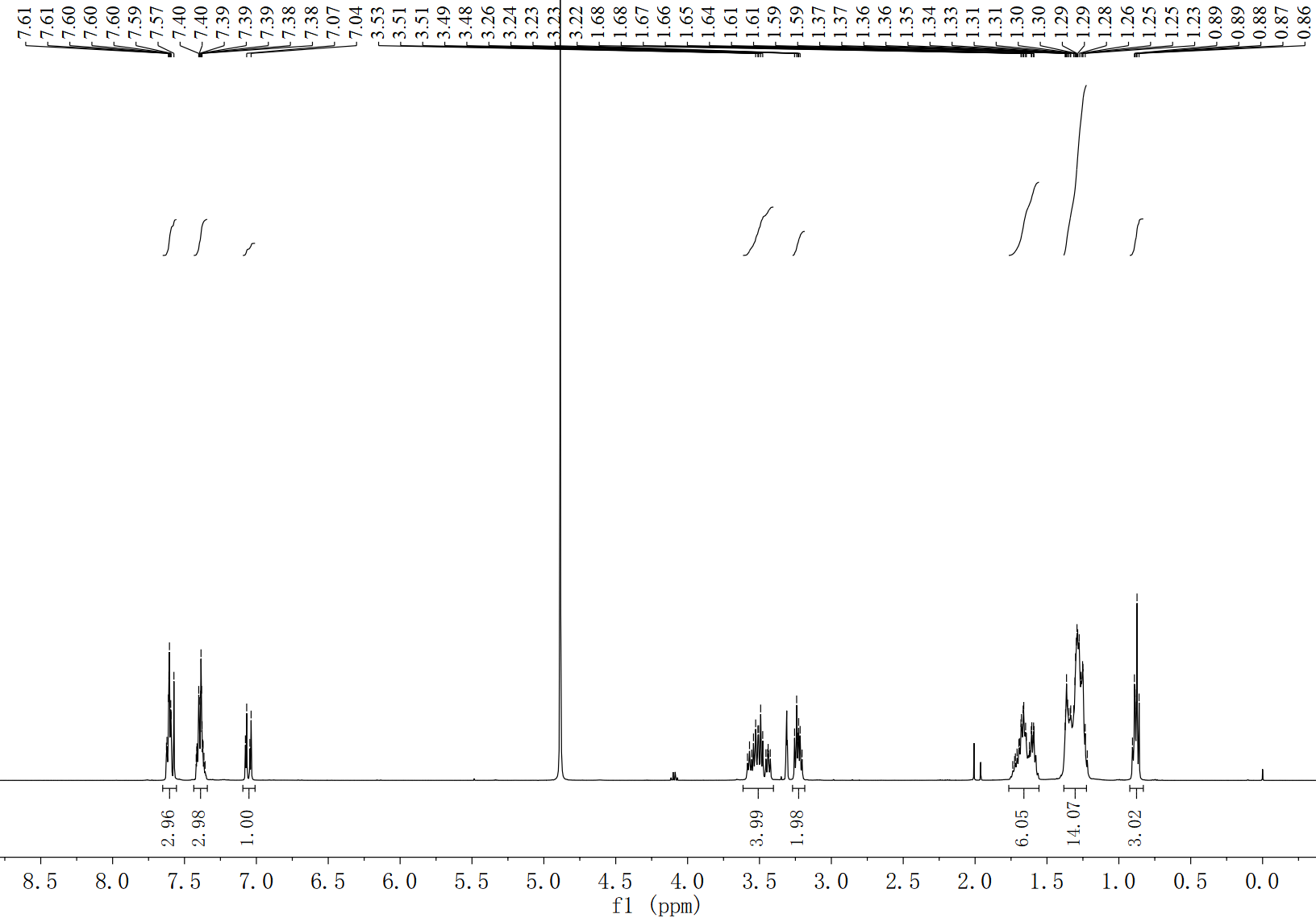

**^1^H NMR spectrum of 10d**


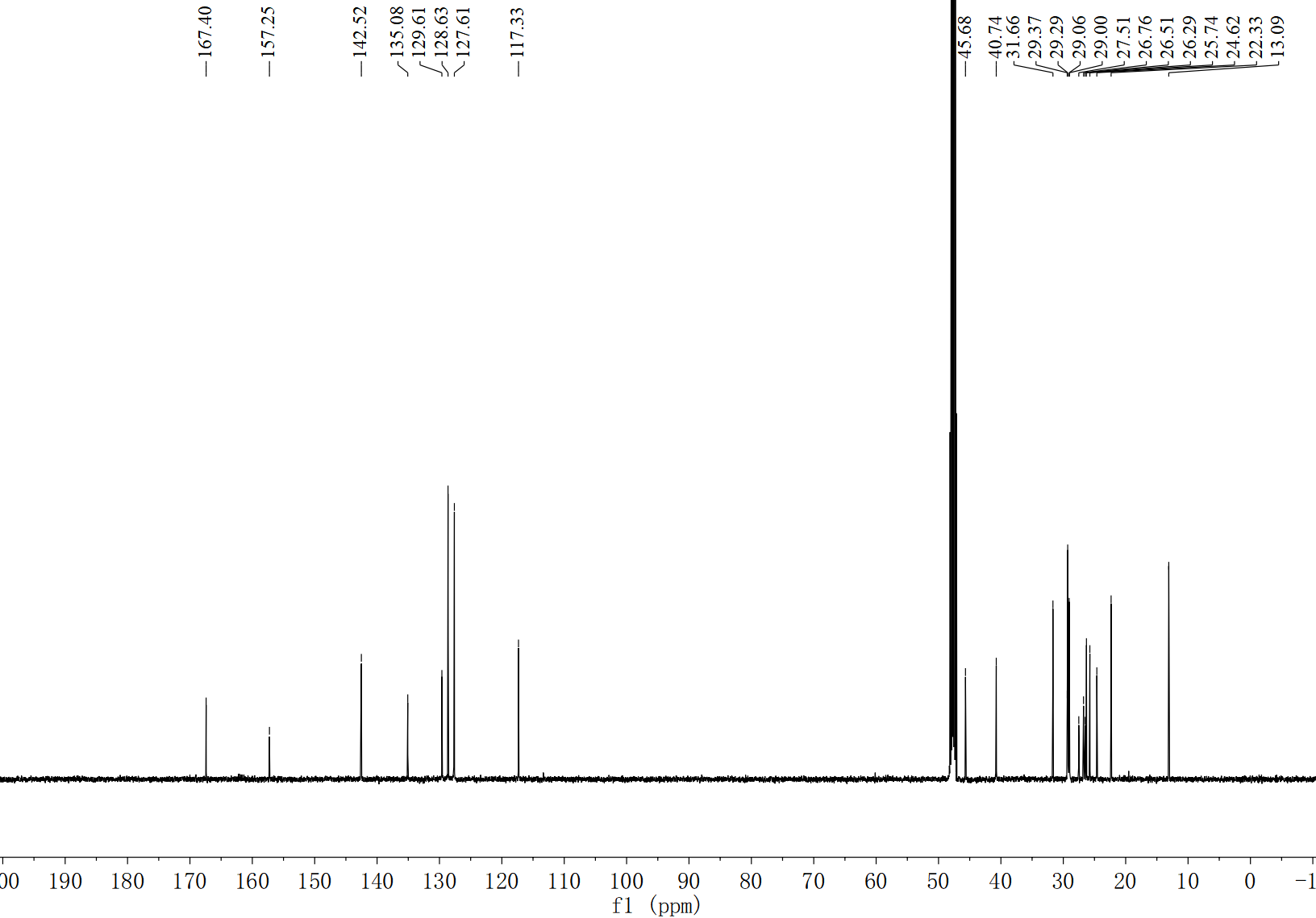


**^13^C NMR spectrum of 10d**


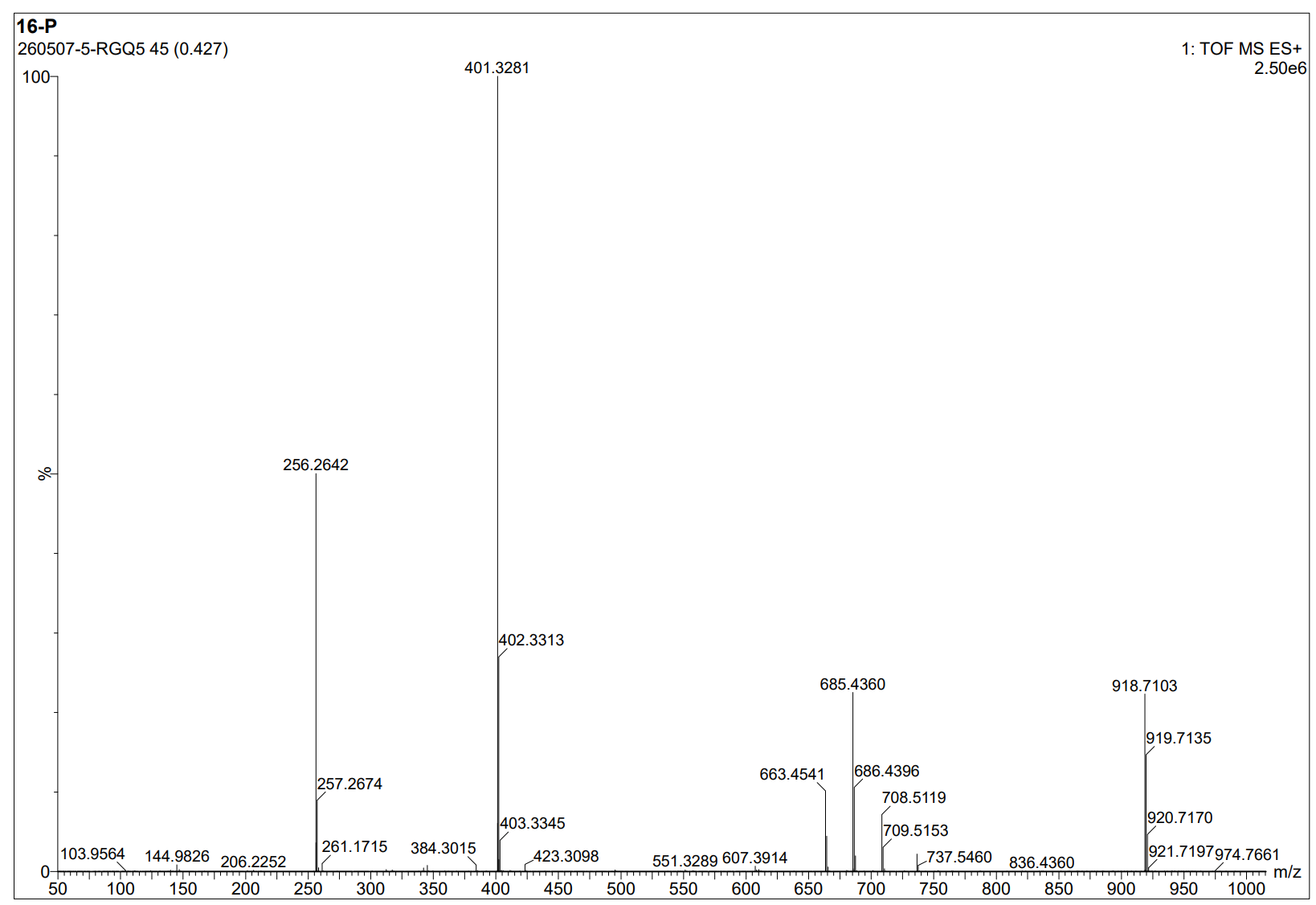


**HRMS spectrum of 10d**


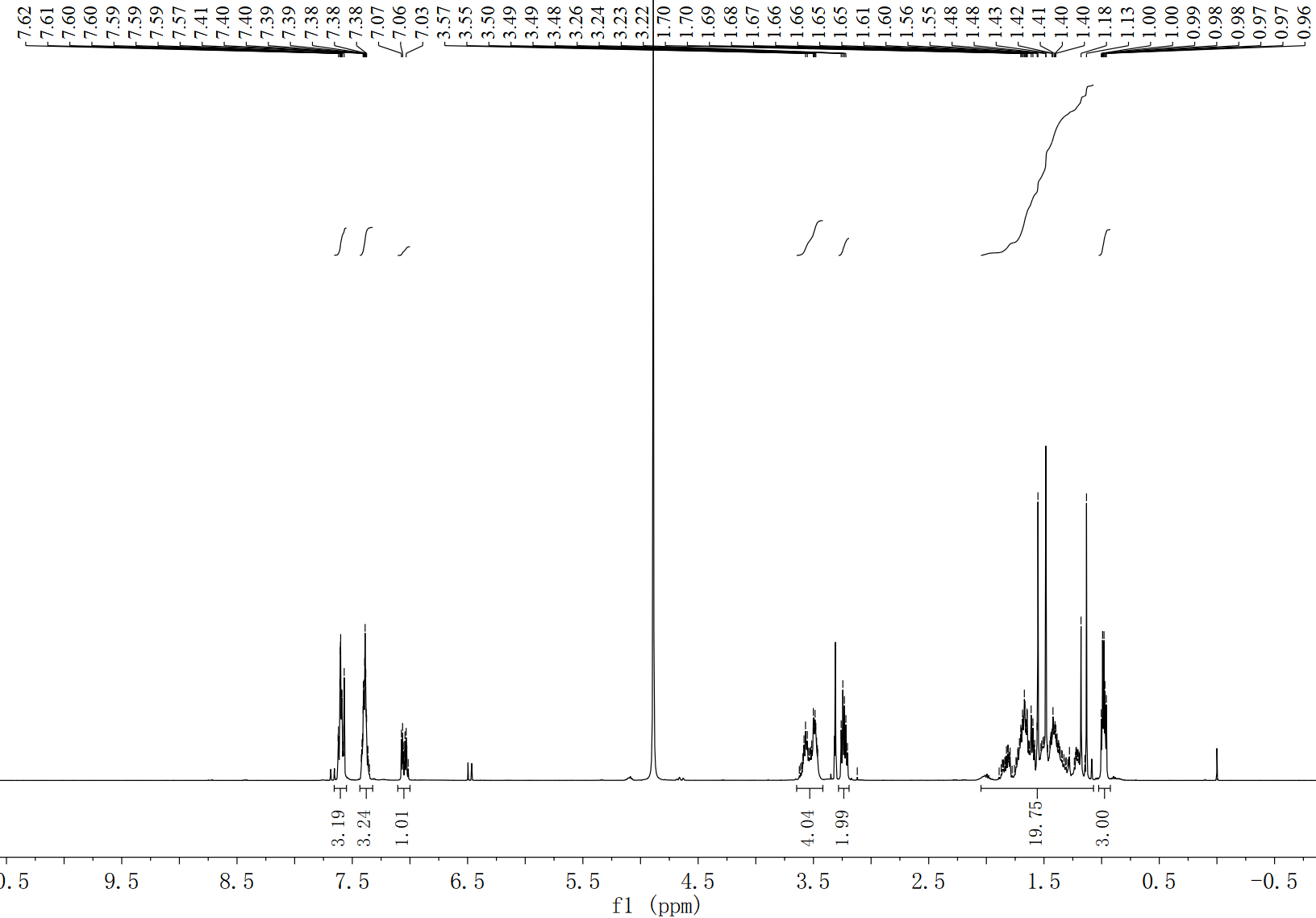

**^1^H NMR spectrum of 10e**


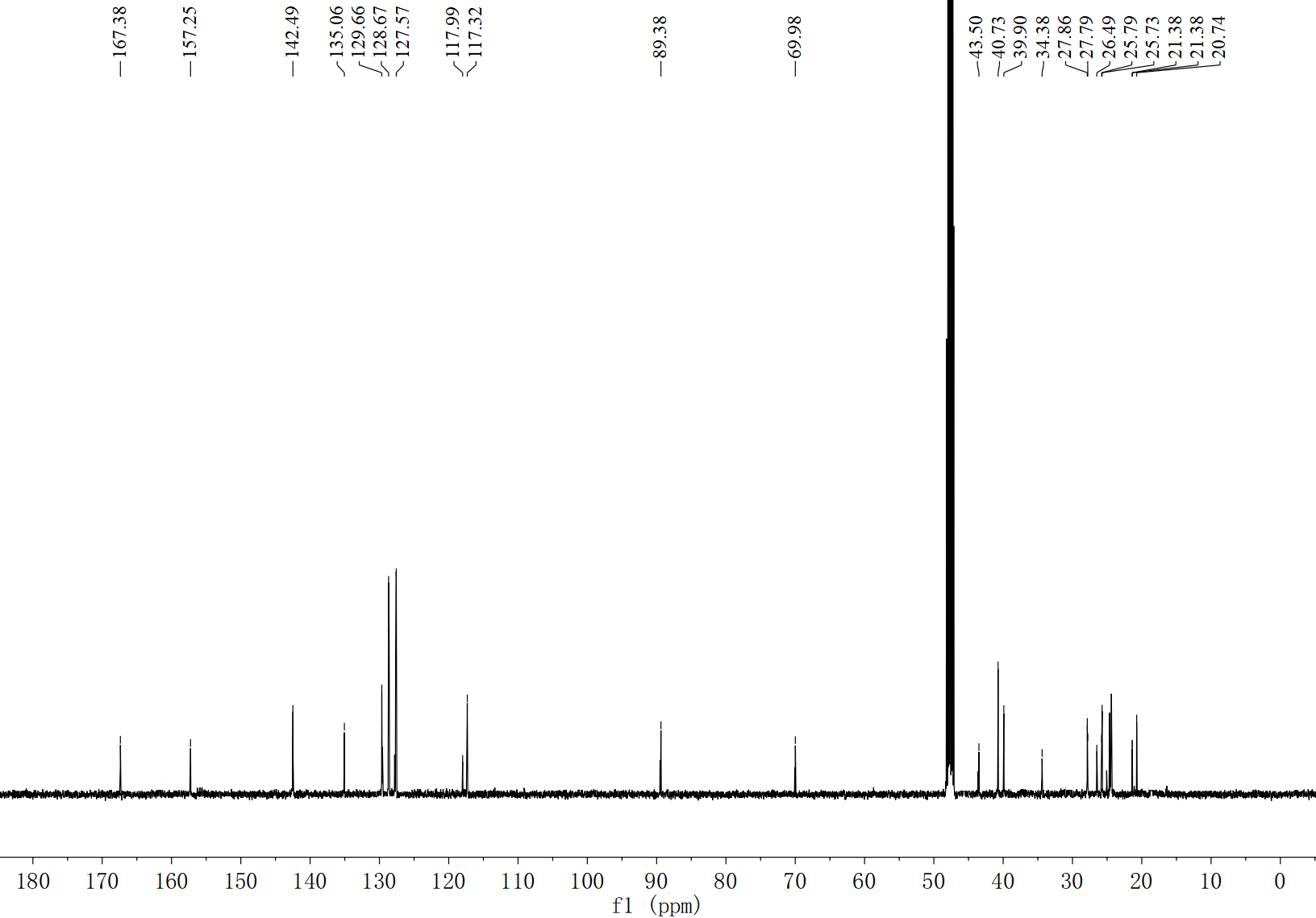


**^13^C NMR spectrum of 10e**


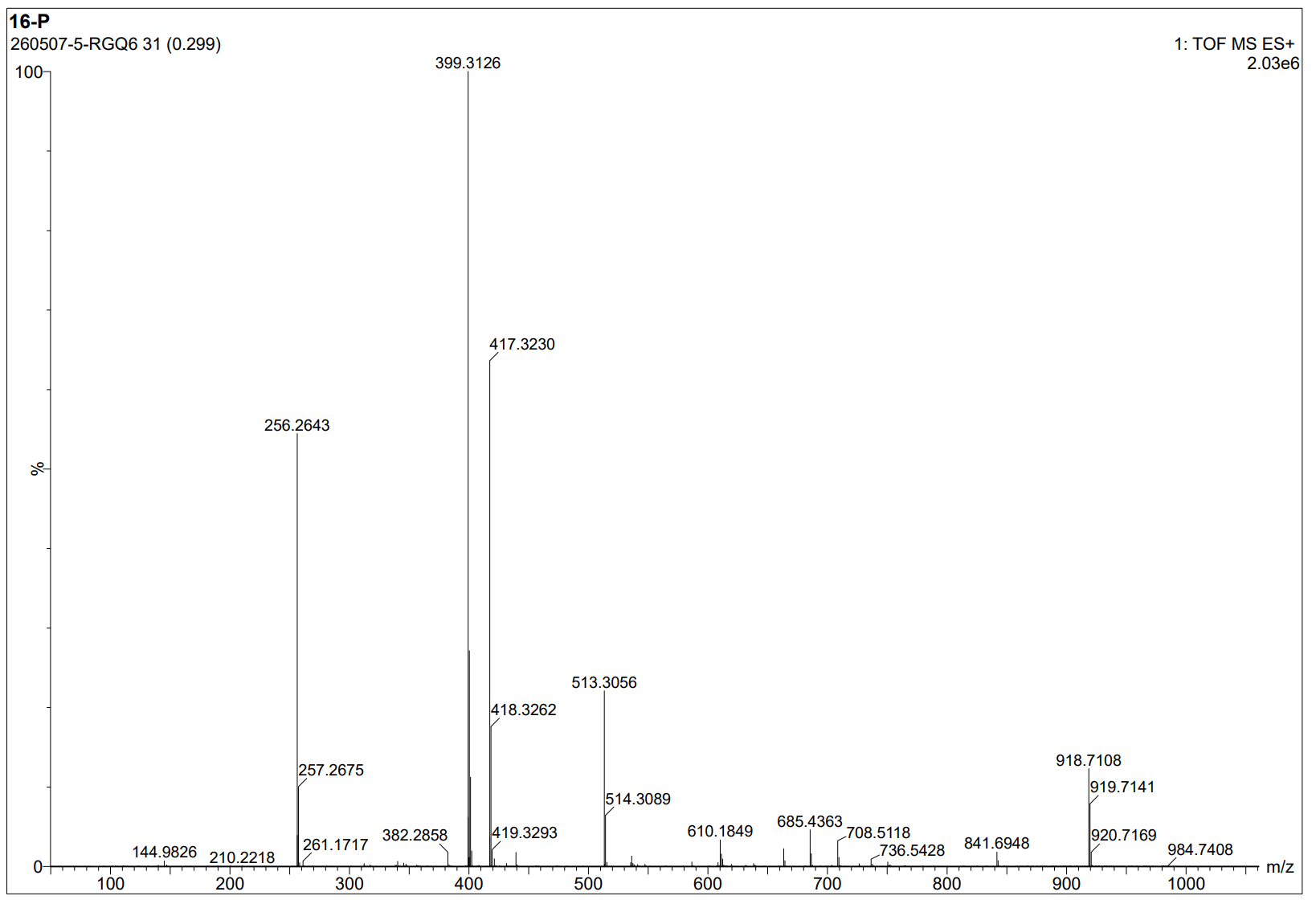


**HRMS spectrum of 10e**


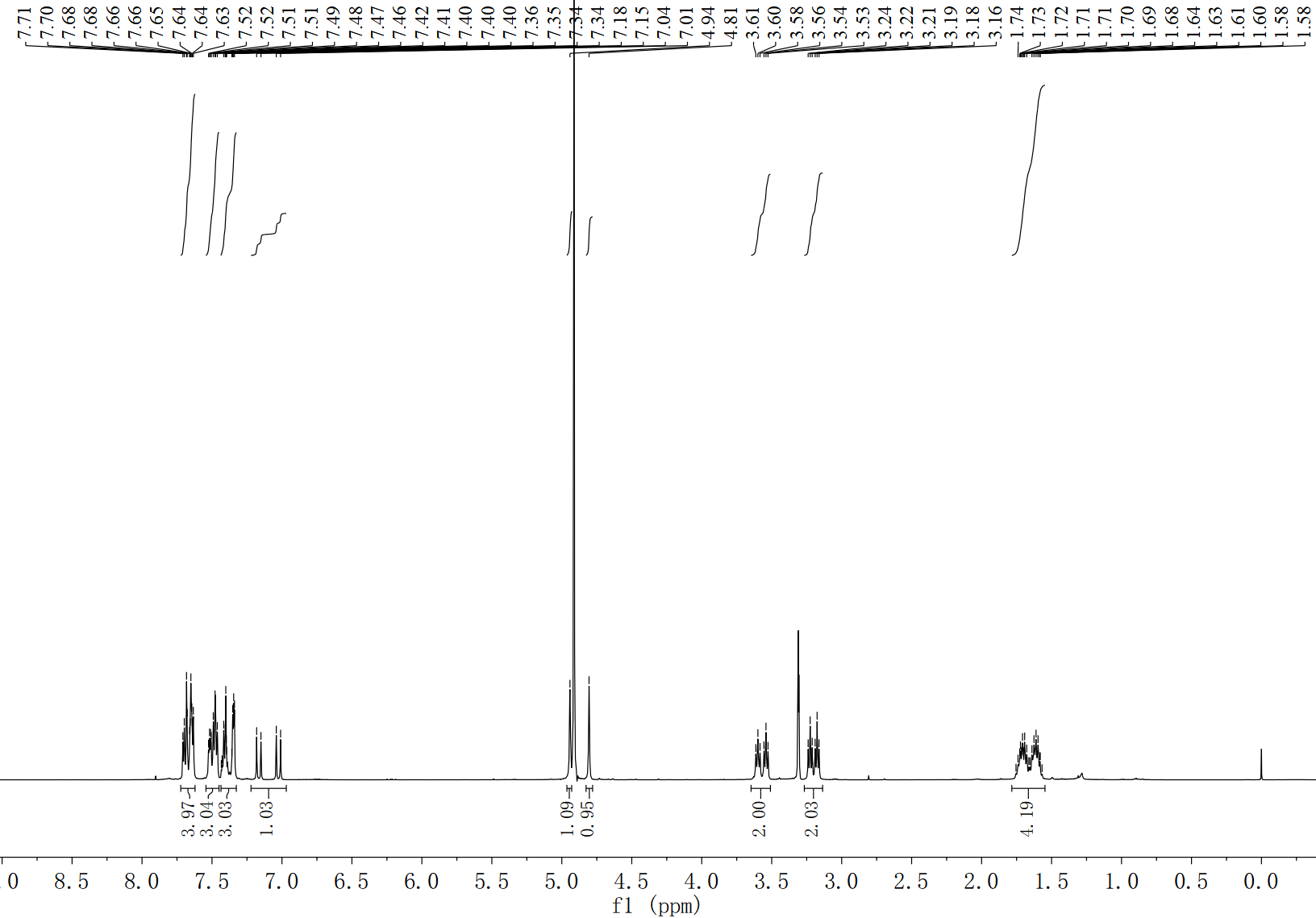

**^1^H NMR spectrum of 10f**


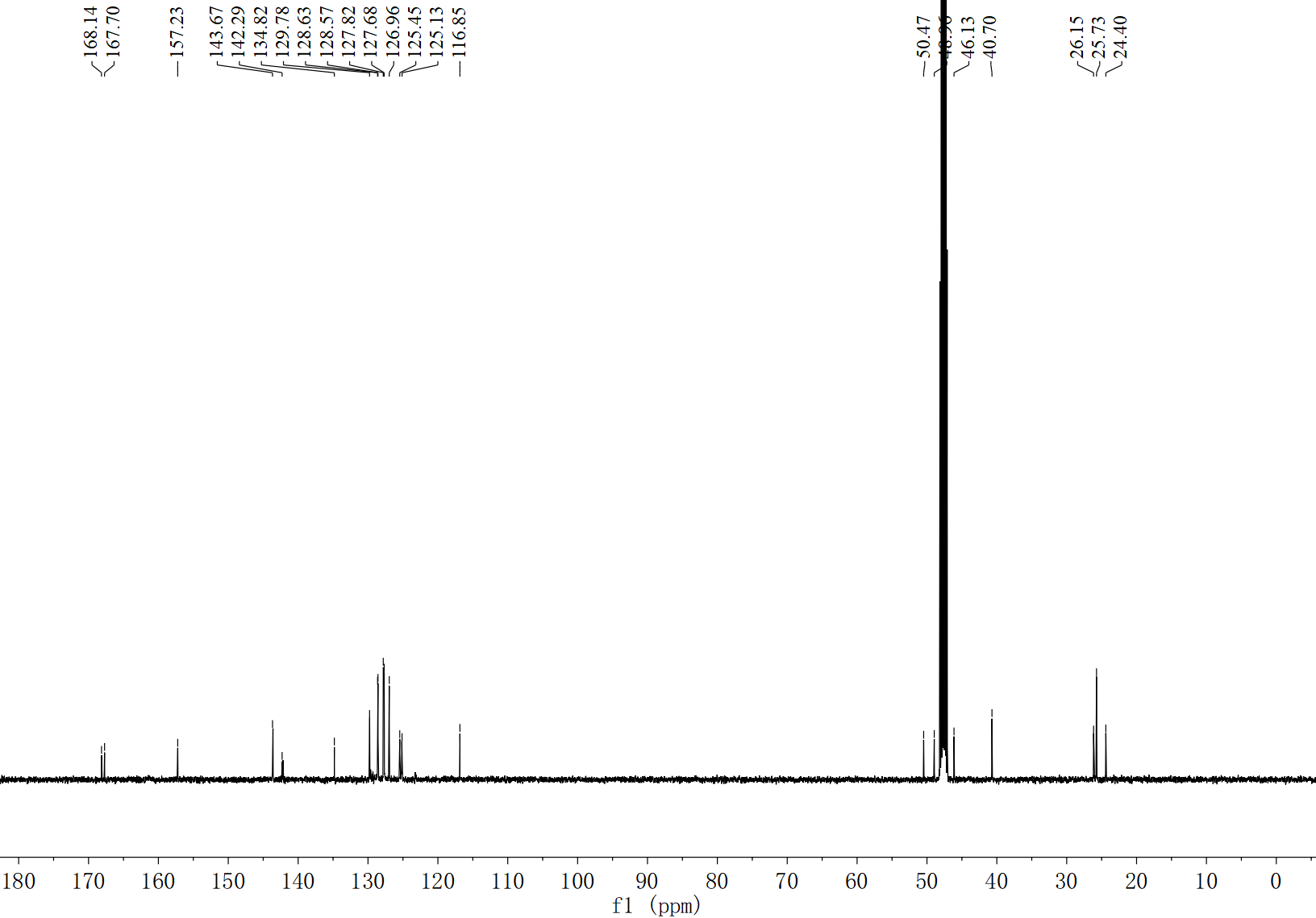


**^13^C NMR spectrum of 10f**


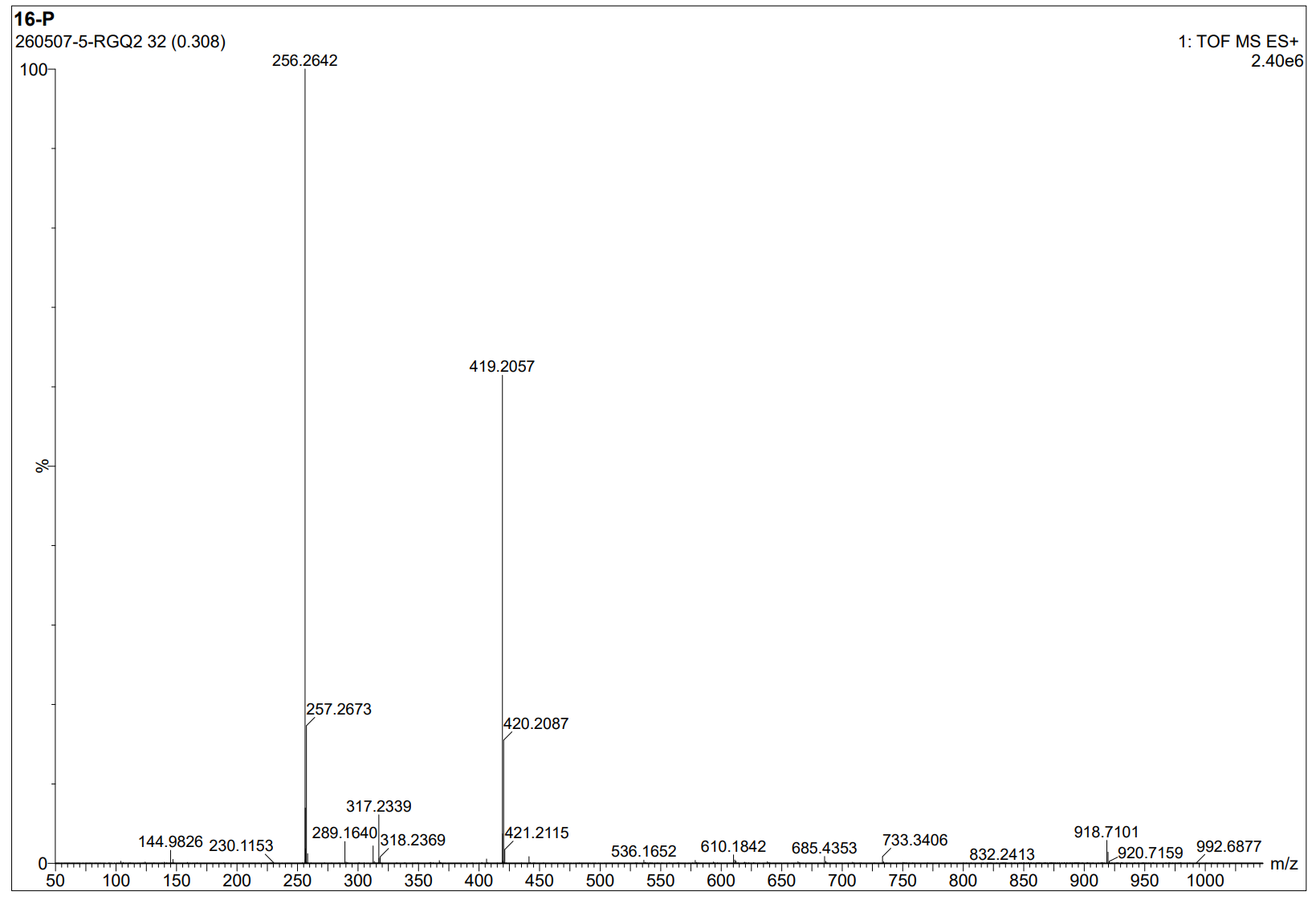


**HRMS spectrum of 10f**


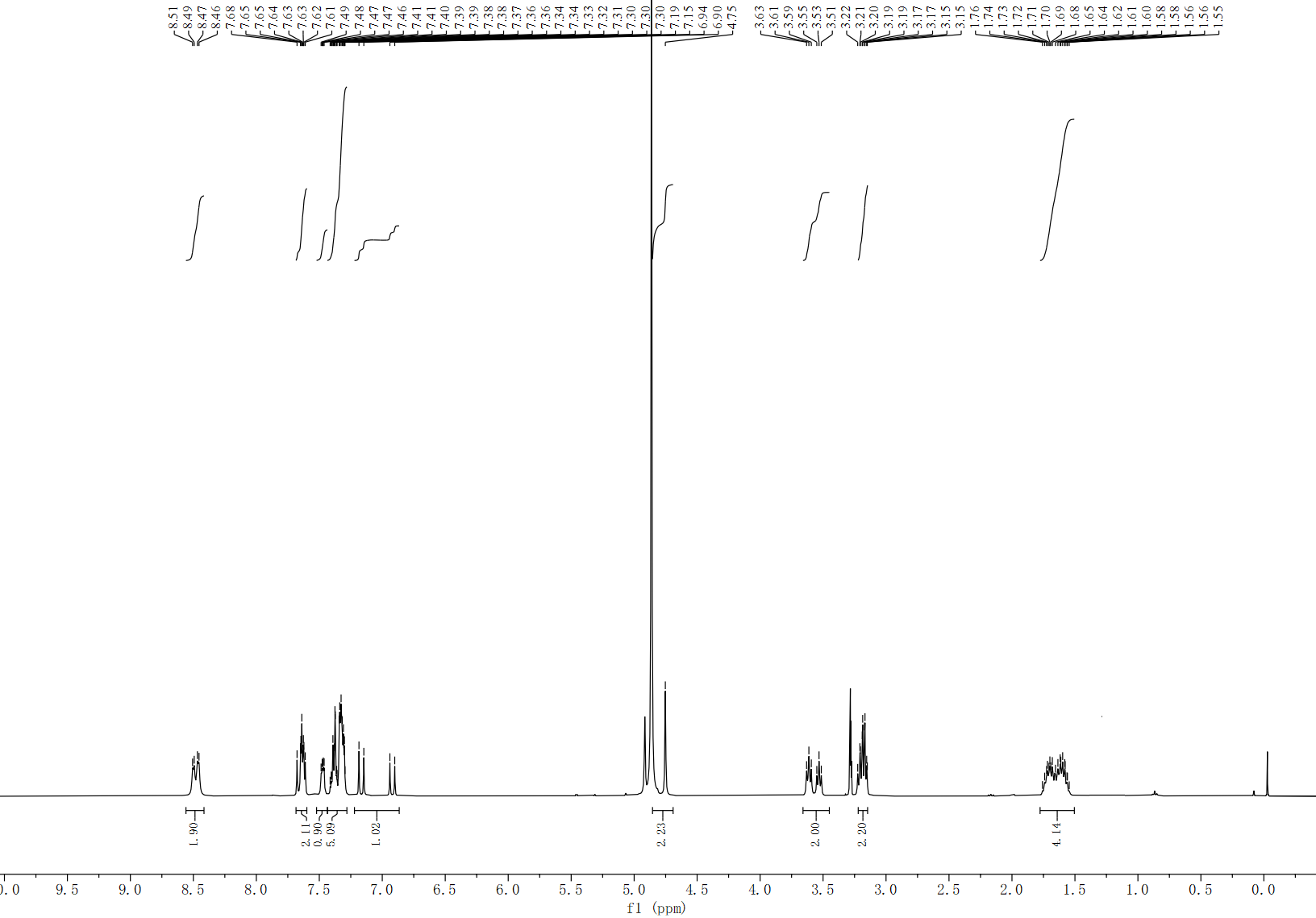

**^1^H NMR spectrum of 10g**


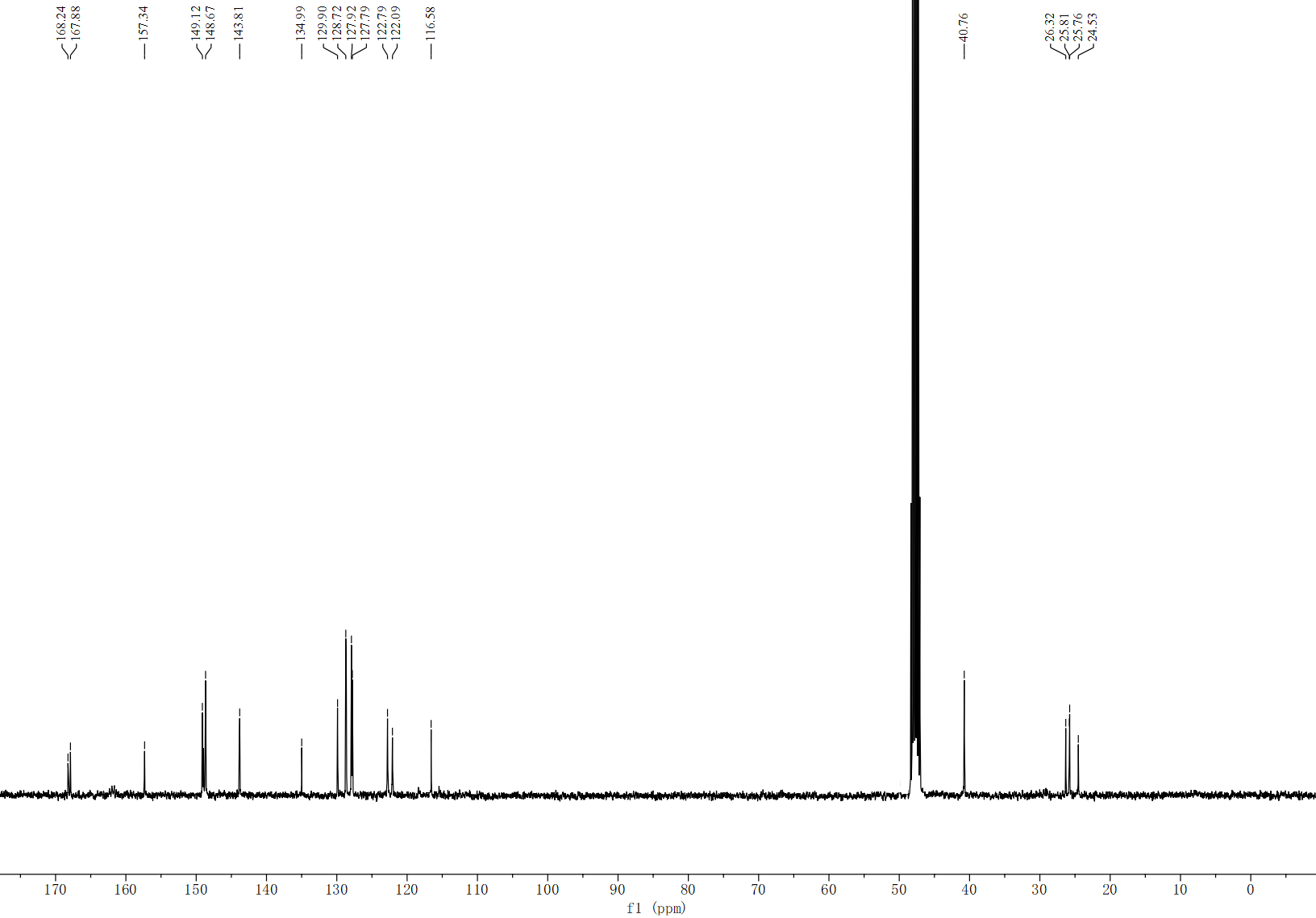


**^13^C NMR spectrum of 10g**


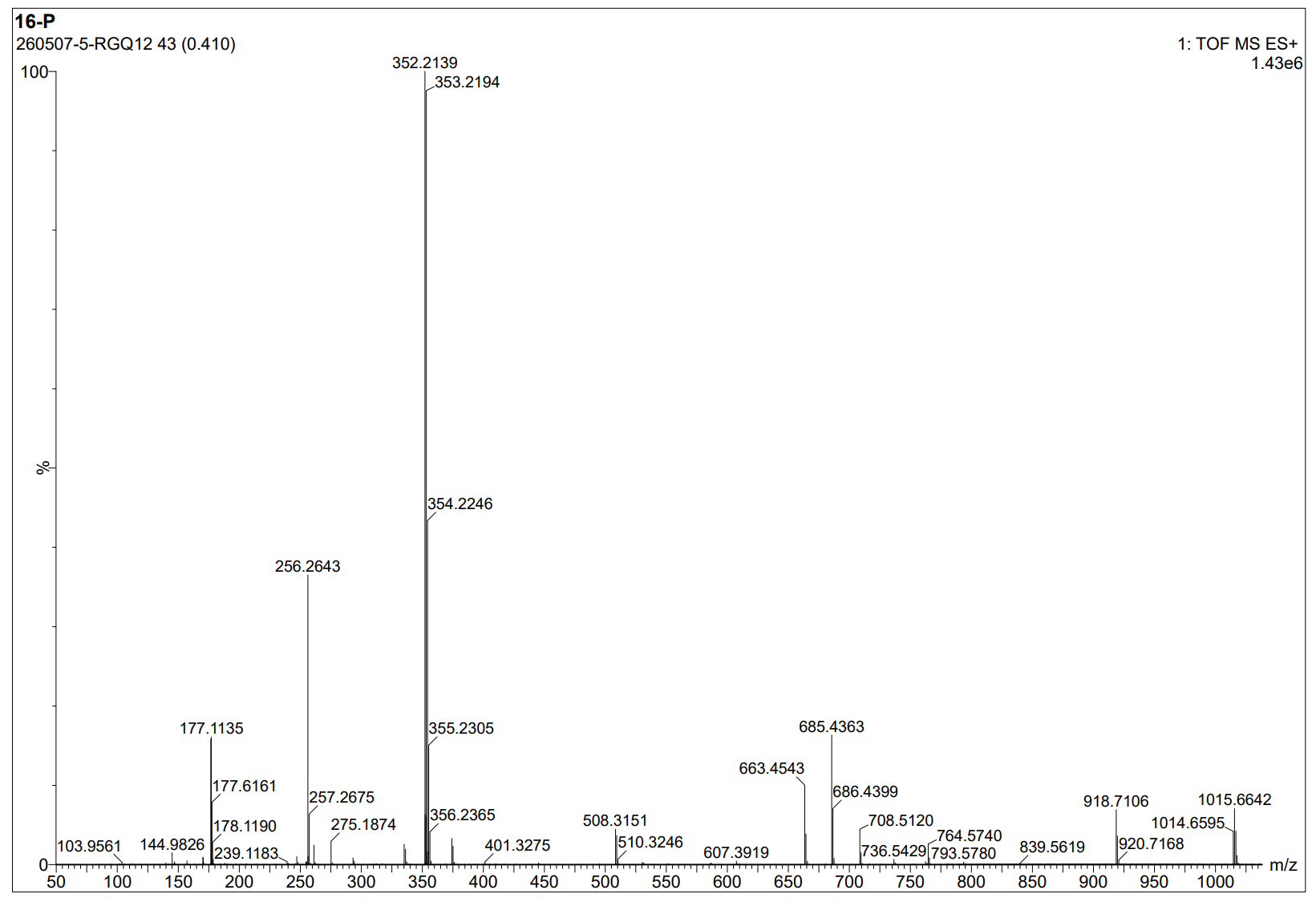


**HRMS spectrum of 10g**


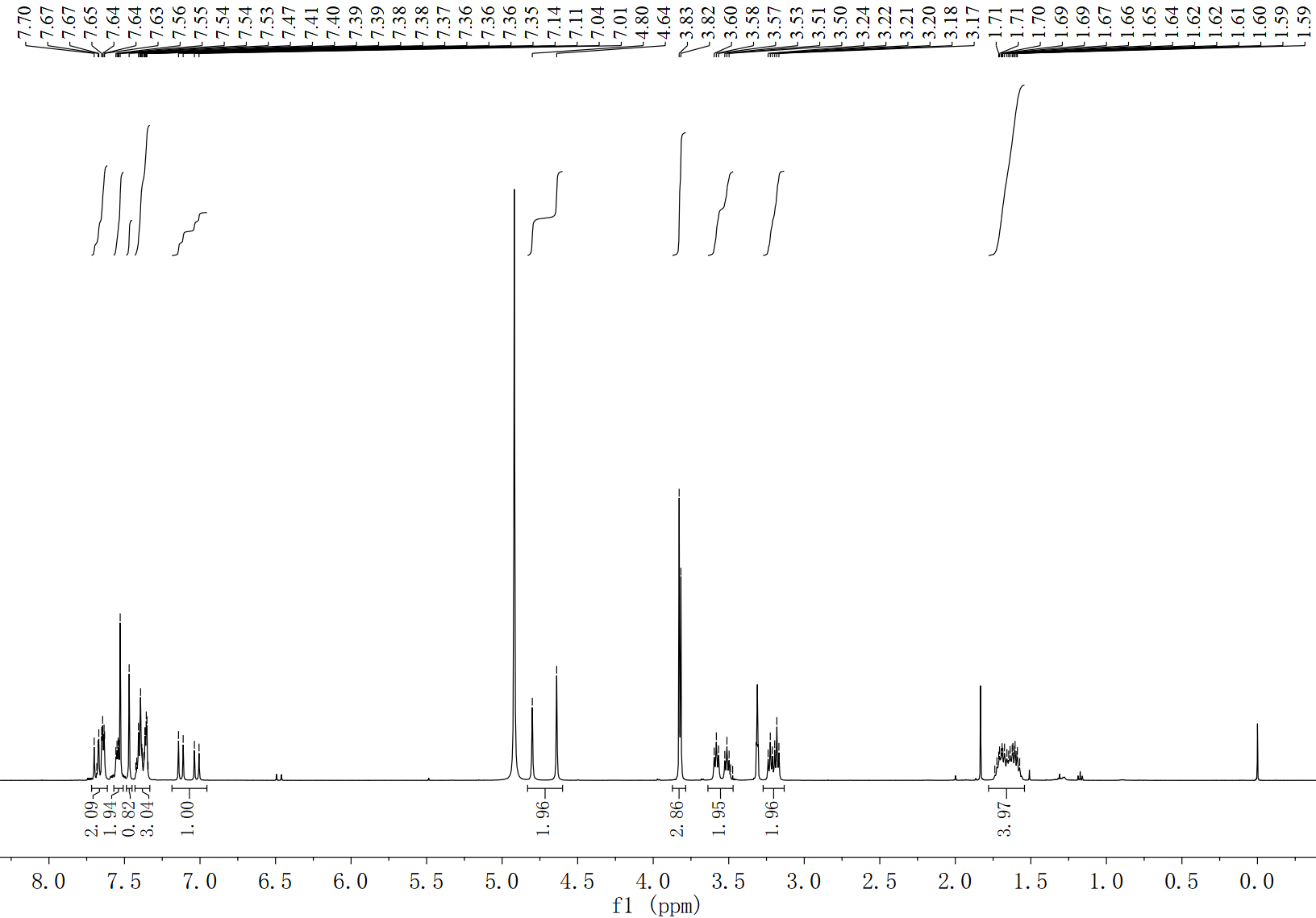

**^1^H NMR spectrum of 10h**


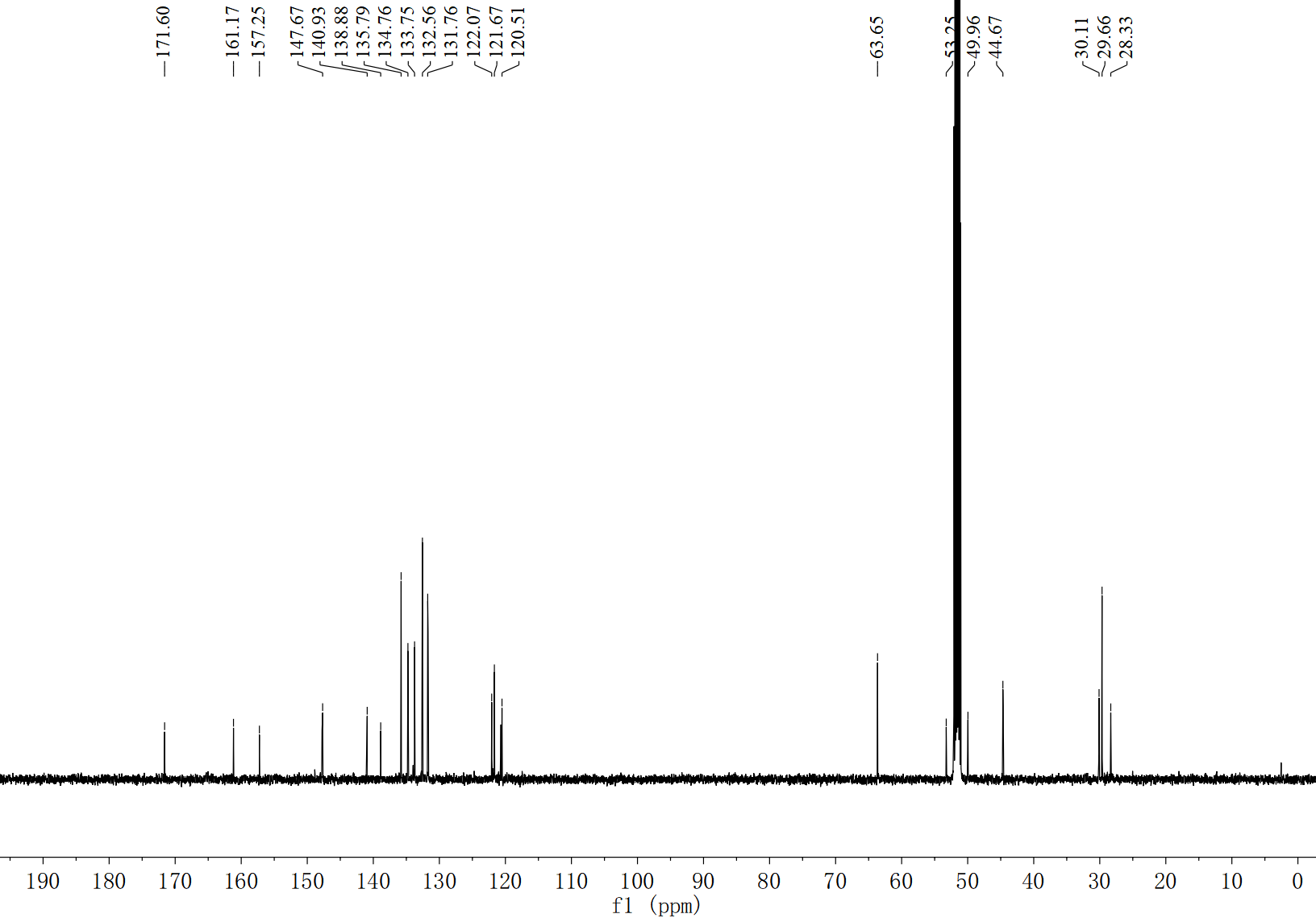


**^13^C NMR spectrum of 10h**


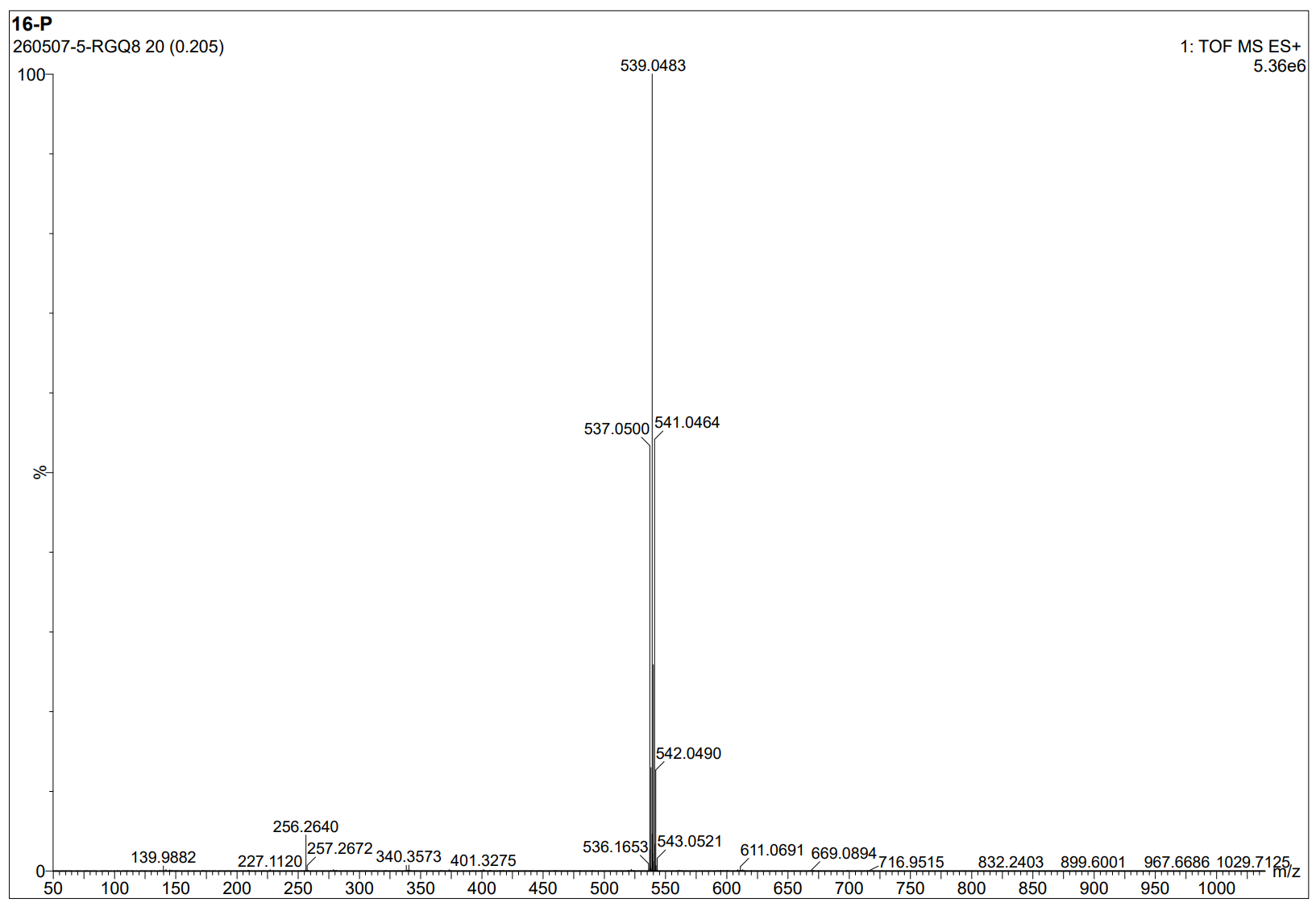


**HRMS spectrum of 10h**
